# Effectiveness of interventions to manage acute malnutrition in children under 5 years of age in low‐ and middle‐income countries: A systematic review

**DOI:** 10.1002/cl2.1082

**Published:** 2020-04-09

**Authors:** Jai K. Das, Rehana A. Salam, Marwah Saeed, Faheem Ali Kazmi, Zulfiqar A. Bhutta

**Affiliations:** ^1^ Division of Women and Child Health Aga Khan University Hospital Karachi Pakistan; ^2^ Division of Women and Child Health, Aga Khan University Karachi Pakistan; ^3^ Centre for Global Child Health, The Hospital for Sick Children Toronto Ontario Canada

## Abstract

**Background:**

Childhood malnutrition is a major public health concern as it is associated with significant short‐ and long‐term morbidity and mortality.

**Objectives:**

To comprehensively review the evidence for the management of severe acute malnutrition (SAM) and moderate acute malnutrition (MAM) according to the current World Health Organization protocol using facility‐ and community‐based approaches as well as the effectiveness of ready‐to‐use therapeutic food (RUTF), ready‐to‐use supplementary food (RUSF), prophylactic antibiotic use and vitamin A supplementation.

**Search methods:**

We searched relevant electronic databases till 11 February 2019. No date or language restrictions were applied.

**Selection criteria:**

We included randomised controlled trials (RCTs) and quasi‐experimental studies including controlled before‐after (CBA) studies and interrupted time series (ITS) studies.

**Data collection and analysis:**

Two review authors independently screened studies for relevance, extracted data, assessed risk of bias and rated the quality of the evidence using the GRADE approach. We carried out statistical analysis using Review Manager software and set out the main findings of the review in “Summary of findings” tables.

**Main results:**

This review summarises findings from a total of 42 studies (48 papers) including 35,017 children. Thirty‐three of the included studies were RCTs; six studies were quasi‐experimental and three studies were cost studies. Majority of the studies were judged to be at high risk of bias for blinding of the participants, personnel and outcome assessment. Majority of the outcomes were rated as either moderate or low quality. Outcomes were downgraded mainly due to study limitations, high heterogeneity, imprecision and small sample size. *Community‐based strategies to screen and manage SAM/MAM versus no community‐based strategies (two studies)*: Integrated community‐based management probably improves recovery rate by 4% [risk ratio (RR): 1.04; 95% confidence interval (CI): 1.00 to 1.09; one study; 1,957 participants; moderate‐quality outcome], and reduces weight gain by 0.8 g·kg^−1^·day^−1^ [mean difference (MD): −0.80 g·kg^−1^·day^−1^; 95% CI: −0.82 to −0.78; one study; 1,957 participants; moderate‐quality outcome] compared with no community‐based strategies, while mortality was similar between the two groups (RR: 0.93; 95% CI: 0.60 to 1.45; one study; 1,957 participants; moderate‐quality outcome). *Facility‐based strategies to screen and manage uncomplicated SAM versus other standard of care (four studies)*: There was no evidence of effect on recovery (RR: 1.00; 95% CI: 0.80, 1.25; one study; 60 participants; very‐low‐quality evidence) and mortality (RR: 1.21; 95% CI: 0.75 to 1.94; two studies; 473 participants; low‐quality outcome). *Facility‐based management with RUTF versus F100 (“catch‐up” formula to rebuild wasted tissues containing 100 kcal and 2.9 g protein per 100 ml) for SAM (three studies)*: There was no evidence of effect on weight gain (MD: 2 g·kg^−1^·day^−1^; 95% CI: −0.23 to 4.23; three studies; 266 participants; very‐low‐quality outcome) and mortality (RR: 1.20; 95% CI: 0.34 to 4.22; two studies; 168 participants; low‐quality outcome). *Community‐based management of SAM with standard RUTF compared with other foods (14 studies)*: There was no evidence of effect on recovery rate when standard RUTF was compared to non‐milk/peanut butter‐based RUTF (RR: 1.03; 95% CI: 0.99 to 1.08; five studies; 5743 participants; I^2^ 50%; moderate quality outcome), energy‐dense, home‐prepared food (RR: 1.14; 95% CI 0.95 to 1.36; four studies; 959 participants; I^2^ 75%; low quality outcome), or high oleic RUTF (RR: 1.06; 95% CI: 0.85 to 1.31; one study; 141 participants; moderate quality outcome). Standard RUTF may improve weight gain by 0.5 g·kg^−1^·day^−1^ (MD: 0.5 g·kg^−1^·day^−1^; 95% CI: 0.02 to 0.99; three studies; 3,069 participants; low‐quality outcome) when compared with non‐milk/peanut butter‐based RUTF and by 5.5 g·kg^−1^·day^−1^ when compared with F100 (MD: 5.50 g·kg^−1^·day^−1^; 95% CI: 2.92 to 8.08; one study; 70 participants; low‐quality outcome). There was no evidence of effect on mortality when standard RUTF was compared with other foods (RR: 0.99; 95% CI: 0.69 to 1.41; nine studies; 7,667 participants; low‐quality outcome). *RUSF for MAM compared with other foods (14 studies)*: There was no evidence of effect on recovery rate when standard RUSF was compared with local/home made food (RR: 0.92; 95% CI: 0.64 to 1.33; three studies; 435 participants; low‐quality outcome) and whey RUSF (RR: 0.96; 95% CI: 0.92 to 1.00; one study; 2230 participants; high‐quality outcome); while standard RUSF may improve recovery by 7% when compared with corn–soy blend (CSB) (RR: 1.07; 95% CI: 1.02 to 1.13; six studies; 5,744 participants; low‐quality outcome). There was no evidence of effect on weight gain when standard RUSF was compared with local home made food (MD: −0.75 g·kg^−1^·day^−1^; 95% CI: −2.03 to 0.43; one study; 73 participants; low‐quality outcome) and whey RUSF (MD: −0.16 g·kg^−1^·day^−1^; 95% CI: −0.33 to 0.01; one study; 2,230 participants; high‐quality outcome); while standard RUSF may improve weight gain by 0.49 g·kg^−1^·day^−1^ when compared with CSB (MD: 0.49 g·kg^−1^·day^−1^; 95% CI: 0.10 to 0.87; five studies; 4,354 participants; low‐quality outcome). There was no evidence of effect on mortality when standard RUSF was compared with other foods (RR: 0.98; 95% CI: 0.57 to 1.68; eight studies; 8,310 participants; moderate‐quality outcome). *Prophylactic antibiotic versus no antibiotic (three studies)*: Prophylactic antibiotic therapy for uncomplicated SAM improves recovery rate by 6% (RR: 1.06; 95% CI: 1.03 to 1.08; two studies; 5,166 participants; high‐quality outcome), probably improves weight gain by 0.67 g·kg^−1^·day^−1^ (MD: 0.67 g·kg^−1^·day^−1^; 95% CI: 0.28, 1.06; two studies; 5,052 participants; moderate‐quality outcome) and probably reduces mortality by 26% (RR: 0.74; 95% CI: 0.55, 0.98; three studies; 6944 participants; moderate quality outcome) compared to no antibiotics group. *High‐dose vitamin A versus low‐dose vitamin A (two studies)*: There was no evidence of effect on weight gain (MD: 0.05 g·kg^−1^·day^−1^; 95% CI: −0.08 to 0.18; one study; 207 participants; moderate‐quality outcome) and mortality (RR: 7.07; 95% CI: 0.37 to 135.13; one study; 207 participants; moderate‐quality outcome).

**Authors’ conclusions:**

Limited data show some benefit of integrated community‐based screening, identification and management of SAM and MAM on improving recovery. Facility‐based screening and management of uncomplicated SAM has no benefit on recovery and mortality, while the effect of F100 for SAM is similar to RUTF for weight gain and mortality. Local food and whey RUSF have similar effects as standard RUSF on recovery rate and weight gain in MAM, while standard RUSF has additional benefits to CSB. Prophylactic antibiotic administration in uncomplicated SAM improves recovery rate, weight gain and reduces mortality, while limited data suggest that high‐dose vitamin A supplementation is comparable with low‐dose vitamin A supplementation for weight gain and mortality among children with SAM.

## 1 PLAIN LANGUAGE SUMMARY

### 1.1 Community‐based approaches can benefit children under five with malnutrition

Many of the interventions to tackle moderate and severe acute malnutrition have similar outcomes. Community and out‐patient‐based approaches are to be preferred on grounds of improved recovery and cost‐effectiveness. Prophylactic antibiotics improve recovery, weight gain and mortality.

#### 1.1.1 What is this review about?

Malnutrition among children under 5 years old is a major public health concern. This review assesses the evidence for the management of severe (SAM) and moderate acute malnutrition (MAM) according to the current World Health Organization (WHO) protocol, using facility‐ and community‐based approaches. It also assesses the effectiveness of ready‐to‐use therapeutic food (RUTF), ready‐to‐use supplementary food (RUSF), prophylactic antibiotic use and vitamin A supplementation.

#### 1.1.2 What is the aim of this review?

This Campbell systematic review summarises findings from 42 studies to inform policy on malnutrition among children under 5 years old.

#### 1.1.3 What studies are included?

A total of 42 studies (48 papers) with 35,017 children were included in this review. All the studies were conducted in either community, hospital, health centre or nutrition rehabilitation centres in developing countries. All the studies targeted children with malnutrition, aged from 6 to 59 months. Thirty‐three of the included studies were randomised controlled trials (RCTs). Six studies were quasi‐experimental and three studies were cost studies.

#### 1.1.4 Do programmes to combat severe and acute malnutrition work?

The included studies are studies with active controls, which means they compare one treatment with another. A finding of no effect means that the treatment does not work any better than the comparison treatment, and not that it does not work at all. The exception is the study of prophylactic antibiotics which are compared with no treatment.

Overall, the evidence shows that none of the interventions studied has any larger effect than the interventions to which they are compared. Prophylactic antibiotics given to children with SAM without complications can affect mortality.

For the other outcomes—recovery and weight gain—the evidence shows the following:

– Community‐based approaches are better than standard care and in‐patient management for recovery, but worse for weight gain and show no effect on mortality.
– Most comparisons of different food preparations find no differences in effects.
– However, for MAM, RUSF is better than corn–soy blend (CSB) for recovery, as is standard RUSF compared with whey RUSF. RUSF is better than CSB for improving weight gain. There are no differences in mortality in these cases.
– Standard dairy/peanut butter RUTF has a positive effect on weight gain compared with non‐/reduced dairy/peanut butter and F100 for uncomplicated SAM.
– There is no effect on weight gain and mortality when high‐dose vitamin A is compared with low‐dose vitamin A supplementation.

The only comparison showing positive effects on all three outcomes (recovery, weight gain and mortality) is prophylactic antibiotic compared with no antibiotic.

The quality of evidence is low, with high risk of bias, partly because of lack of blinding of the participants, personnel and outcome assessment. There is also high heterogeneity between studies, which is partly explained by imprecision on account of small sample size.

#### 1.1.5 What do the findings of this review mean?

The evidence shows the equivalence of many approaches, so that decisions may be made on cost grounds. Existing limited cost data suggest that community or out‐patient management of children with uncomplicated SAM and MAM is the most cost‐effective strategy.

The evidence base remains thin and study quality is a concern. Future studies assessing the effectiveness of interventions should report pertinent nutrition‐specific outcomes, including stunting, wasting, underweight, infections and potential adverse effects. Further studies should assess the relative cost and cost‐effectiveness of various interventions addressing malnutrition in low‐ and middle‐income countries.

#### 1.1.6 How up‐to‐date is this review?

The review authors searched for studies published up to February 2019.

## 2 BACKGROUND

### 2.1 Description of the condition

Childhood undernutrition includes wasting [weight‐for‐height *z*‐score (WHZ) < −2 standard deviation (*SD*)], stunting [height‐for‐age *z*‐score (HAZ) < −2 *SD*], underweight [weight‐for‐age *z*‐score (WAZ) < −2 *SD*] and micronutrient deficiencies or insufficiencies (WHO, 2017b). The current WHO guidelines subsume these entities into the blanket term of childhood malnutrition which is broadly categorised into acute and chronic malnutrition. Acute malnutrition is further classified on the basis of severity into MAM (WHZ between −3 and −2) and SAM [WHZ < −3 and mid‐upper arm circumference (MUAC) < 115 mm] whereas chronic malnutrition occurs due to long‐term insufficient intake of nutrients and a complex interplay of intergenerational and environmental factors and results in stunting (UNICEF, 2009). In 2017, an estimated 155 million children under 5 years of age were stunted and 52 million were wasted (Development Initiatives, 2017). Around 45% of death among children under 5 years of age is associated with undernutrition (WHO, 2017b). Asia and Africa still share the greatest burden of malnutrition with more than half of all stunted children and two‐third of all wasted children under 5 years of age living in Asia and over one‐third stunted children and a quarter of wasted children living in Africa (UNICEF, 2017).

Childhood malnutrition is a major public health concern since it is associated with significant morbidity and mortality (WHO, 2017a). The consequences of malnutrition among infants and children can be short‐term like morbidity, mortality and disability or long‐term including impaired cognitive development, increased risk of disease due to either concurrent infections or metabolic disorders and suboptimal economic productivity (Black et al., 2013). Undernutrition, including stunting, severe wasting, deficiencies of vitamin A and zinc and sub‐optimum breastfeeding, has been an underlying cause of approximately one‐third of the mortality among children under 5 years of age (Black et al., 2013; De Onis, Brown, Blossner, & Borghi, 2012). Childhood malnutrition is a result of a complex interplay of nutrition‐specific and nutrition‐sensitive factors. Nutrition‐specific factors include inadequate food and nutrient intake, poor feeding, care giving and parenting practices, and burden of infectious diseases while nutrition‐sensitive factors include food insecurity; inadequate care giving resources at the maternal, household and community levels and limited access to health services and unhygienic environment (Bhutta et al., 2013). Improving childhood malnutrition requires effective implementation of nutrition‐sensitive as well as nutrition‐specific interventions (Ruel, Alderman, & Maternal Child Nutrition Study Group, 2013).

### 2.2 Description of the intervention

The existing WHO guidelines for the management malnutrition among children suggests the following (WHO, 2013):

1. Early identification of children with SAM in the community through active community screening by trained community health workers (CHWs) and community members. CHWs should measure the MUAC of infants and children under 5 years of age and examine them for bilateral pitting oedema.
2. Assessment of nutrition status in primary health‐care facilities and hospitals through routine health‐facility screening. Health‐care workers should assess the MUAC or the WHZ status of infants and children under 5 years of age and also examine them for bilateral oedema.
3. Children who are identified as having SAM should first be assessed with a full clinical examination to confirm whether they have medical complications and whether they have an appetite. Children who have appetite and are clinically well and alert (uncomplicated SAM) should be treated as outpatients and can be managed with RUTF in amounts adjusted to their weight, to provide recommended energy intakes for recovery while children with complications should be treated inpatient.
4. Children with uncomplicated SAM, not requiring to be admitted and who are managed as outpatients, should be given a course of oral antibiotics such as amoxicillin while children who are undernourished but who do not have SAM should not routinely receive antibiotics unless they show signs of clinical infection. Children admitted with SAM and with no apparent signs of infection and no complications should be given an oral antibiotic.
5. Children who have medical complications, severe oedema (+++), or poor appetite, or present with one or more Integrated Management of Childhood Illness (IMCI) danger signs should be treated as inpatients. Children admitted with SAM and complications such as septic shock, hypoglycaemia, hypothermia, skin infections or respiratory or urinary tract infections, or who appear lethargic or sickly, should be given parenteral antibiotics. Children with SAM who are admitted to hospital can be transferred to outpatient care when their medical complications, including oedema, are resolving and they have good appetite, and are clinically well and alert. The decision to transfer children from inpatient to outpatient care should be determined by their clinical condition and not on the basis of specific anthropometric outcomes. Children with SAM who are discharged from treatment programmes should be periodically monitored to avoid a relapse.
6. F75 and F100 are formula diets used for the management of children with SAM in inpatient care. F75 (75 kcal or 315 kJ/100 ml) is used during the initial phase of treatment, while F100 (100 kcal or 420 kJ/100 ml) is used during the rehabilitation phase. Children with SAM cannot tolerate high amounts of protein and fat and hence they are supplemented with F75 initially; as soon as the child is stabilised on F75, F100 is used as a “catch‐up” formula. Children with SAM who present with either acute or persistent diarrhoea, can be given RUTF in the same way as children without diarrhoea, whether they are being managed as inpatients or outpatients.
7. Children with SAM should receive the daily recommended nutrient intake of vitamin A throughout the treatment period. Children with SAM should be provided with about 5,000 IU vitamin A daily, either as an integral part of therapeutic foods or as part of a multi‐micronutrient formulation.

According to these guidelines, children with complicated SAM are managed as inpatients in three phases; stabilisation phase which includes fluid management for severe dehydration, correction of hypothermia, hypoglycaemia and micronutrient deficiencies and the use of antibiotics for complications; rehabilitation phase which includes increased nutrient and energy intake through therapeutic or fortified foods as well continued electrolyte and micronutrient management. Following recovery, caregivers are given appropriate nutritional training to avoid similar recurrences and instructed on the importance of sensory stimulation in children for continued emotional and physical development (Ashworth, 2003). SAM among children under 6 months of age is increasingly being associated with higher mortality than in older infants and children (WHO, 2013). The WHO guideline suggests that in infants who are under 6 months of age with SAM should receive the same general medical care as infants with SAM who are 6 months of age or older with increased focus on establishing, or re‐establishing, effective exclusive breastfeeding by the mother or other caregiver (WHO, 2013).

In this review, we will assess the effectiveness of various community‐based and facility‐based strategies to identify and manage MAM and SAM; including the community‐based screening, identification management of SAM and MAM, relative effectiveness of RUTF for SAM and RUSF for MAM, effectiveness of prophylactic use of antibiotic to manage uncomplicated SAM and the effectiveness of vitamin A supplementation to manage children with acute malnutrition.

### 2.3 How the intervention might work

Childhood malnutrition results in long‐term disability through cognitive impairment, delayed motor growth, poor physical performance, low‐birth weight of future offspring, behavioural issues and poor academic performance as well as suboptimal productivity in adulthood (Black et al., 2008). The Community Based Management of Malnutrition (CMAM) approach has been introduced for screening and early identification of children with malnutrition to provide timely access to quality care. It enables community volunteers to identify and initiate treatment for children with acute malnutrition before they become seriously ill at home by using RUTF and routine medical care (Ashworth, 2006). The CMAM approach comprises of four components: (a) community outreach and mobilisation; (b) outpatient management of SAM without medical complications; (c) inpatient management of SAM with medical complications and (d) services or programmes to manage MAM, such a supplementary feeding programme (Collins et al., 2006). Early identification of children with SAM in the community is key to prevent complications related to malnutrition and works through early case finding, referral to the management programme and effective follow‐up measures. This requires contextually sensitive approaches through community assessment and mobilisation (Park et al., 2012).

Undernutrition (including all degrees of stunting, wasting, underweight and micronutrient deficiencies) has been associated with infectious diseases and children with SAM may be more susceptible to infection (Black, 2003; Black et al., 2013; Salam, Das, & Bhutta, 2015). Current WHO guidelines suggest that prophylactic administration of antibiotics to children with uncomplicated SAM should be used to treat underlying infections; however the evidence on the current antibiotic recommendation is weak and inconclusive and requires further research considering the side effects, costs, and risks associated with antibiotic administration (Alcoba et al., 2013; Picot et al., 2012).

Supplementary feeding is expected to prevent further deterioration of nutritional status in moderately malnourished children and to restore growth and promote physiological recovery by minimising the nutritional and energy gap (Karakochuk, van den Briel, Stephens, & Zlotkin, 2012). Supplementary foods are considered an effective strategy in the treatment and management of malnutrition either at home, facility or rehabilitation centre (Visser, McLachlan, Fergusson, Volmink, & Garner, 2013). Supplementation promotes recovery by increasing nutrient absorption, thus improving growth and promoting development especially in the first 1,000 days of life which is critical to cognitive function (Imdad, Yakoob, & Bhutta, 2011). A possible adverse effect of supplementary feeding interventions may be excessive and quick weight gain. Studies suggest that rapid weight correction in early childhood to reverse malnutrition can be associated with increased risk of obesity and potentially increased risk of diabetes in adulthood (Adair et al., 2013; Norris et al., 2012).

Micronutrient deficiencies also coexist among malnourished children and supplementation of vital micronutrients including vitamin A and zinc is required to ensure sufficiency and bioavailability within the body (Dairo & Ige, 2009, Mannar, Venkatesh, & Sankar, 2004). Vitamin A and zinc deficiency weakens the immune system of acutely malnourished children and facilitates bacterial invasion thereby increasing the risk of morbidity and mortality due to infectious diseases (Bailey, West, & Black, 2015; Bhutta et al., 1999; Bourke, Berkley, & Prendergast, 2016; Jones & Berkley, 2014; Manary, Iannotti, & Trehan, 2012). However, supplementation should consider the safe upper intake levels and potential toxicology of the specific micronutrient (Renwick, 2006).

### 2.4 Why it is important to do this review

Despite the outlined interventions to manage childhood malnutrition (WHO, 2013), there is uncertainty around the most effective methods to treat malnutrition in young children and lack of clarity in defining comparator groups (Picot et al., 2012).The existing WHO guidelines for the management of malnutrition also highlighted a few priority issues and research gaps (WHO, 2013) that include:

1. Assessing the strategies to improve active community screening and routine health‐facility screening, and investigating barriers to service access and uptake, to enhance treatment coverage.
2. Assessing the clinical effect and cost‐effectiveness of giving oral antibiotics to children and infants with SAM who do not require inpatient management in non‐HIV settings.
3. Assessing the adverse effects of giving broad‐spectrum antibiotics to infants and children with SAM without complications.
4. Assessing the efficacy and effectiveness of different RUSF and RUTF that comply with WHO specifications and are made from different ingredients in different regions of the world (using commercially produced RUTF as the comparison) and the comparative effectiveness of RUTF, RUSF and F100 for recovery of children with MAM and SAM.
5. Assessing the efficacy of daily low‐dose vitamin A supplementation compared with single high‐dose vitamin A in the treatment of children with SAM and the most effective way to improve and sustain the vitamin A status of children with SAM after discharge from treatment.

The above research gaps from the WHO guidelines have not been the topic of a comprehensive systematic review. However, there are a few existing reviews evaluating some interventions separately. Lenters, Wazny, Webb, Ahmed, and Bhutta (2013) undertook a systematic review to evaluate the effectiveness of approaches to managing MAM and SAM according to the WHO protocol, but the results were unclear due to lack of robust trials. Moreover, there are issues related to lack of rigorous estimates due to poor adjustment for confounding variables in observational studies; heterogeneity in participants, recruitment, interventions, settings and units of measurement of outcomes (Lenters et al., 2013). Existing reviews on management of acute malnutrition are either focused on specific population groups; specific interventions (prophylactic use of antibiotics, IV fluid for shock, treatment of diarrhoea, micronutrients deficiencies, etc.); or there is discrepancy in the definition of undernutrition and types of therapeutic or supplementary foods (Alcoba et al., 2013; Gera, 2010; Lazzerini & Tickell, 2011; Picot et al., 2012; Schoonees, Lombard, Musekiwa, Nel, & Volmink, 2019). Moreover, supplementary feeding has been the topic of two reviews (Kristjansson et al., 2015; Visser et al., 2013) and the effectiveness of vitamin A supplementation for the treatment of SAM has also been reviewed (Manary et al., 2012). But there is a need to comprehensively review the evidence for the management of SAM and MAM according to the current WHO protocol using facility‐ and community‐based approaches as well as the effectiveness of RUTF, RUSF, prophylactic antibiotic use and vitamin A supplementation. Therefore, the aim of this systematic review is to analyse and update the evidence on the effectiveness of recommended interventions and to assess the programme and/or guidelines that have been adapted to manage children with acute malnutrition to provide a comprehensive and updated review.

## 3 OBJECTIVES

The objectives of this review are as follows:

1. To evaluate the effectiveness of community‐based strategies such as community‐based mobilisation, screening, follow‐up, counselling and education to improve screening, identification and management of SAM and MAM.
2. To evaluate the effectiveness of facility‐based strategies such as facility‐based screening, management and periodic follow‐up to improve screening and management of SAM and MAM.
3. To evaluate the effectiveness and relative effectiveness of various RUTF and RUSF for the management of SAM and MAM.
4. To evaluate the effectiveness of prophylactic use of antibiotic to manage uncomplicated SAM.
5. To evaluate the effectiveness of various doses of vitamin A supplement to manage children with SAM and MAM.

## 4 METHODS

### 4.1 Criteria for considering studies for this review

#### 4.1.1 Types of studies

We included the following study designs:

- RCTs, where participants were randomly assigned, individually or in clusters, to intervention and comparison groups. Cross‐over designs were also eligible for inclusion.
- Quasi‐experimental designs, which include:

○ Natural experiments: Studies where non‐random assignment was determined by factors that were out of the control of the investigator. One common type includes allocation based on exogenous geographical variation.
○ Controlled before‐after studies (CBA), in which measures were taken of an experimental group and a comparable control group both before and after the intervention. We also require that appropriate methods were used to control for confounding, such as statistical matching (e.g., propensity score matching, or covariate matching) or regression adjustment (e.g., difference‐in‐differences, instrumental variables).
○ Regression discontinuity designs; here, allocation to intervention/control was based upon a cut‐off score.
○ Interrupted time series (ITS) studies, in which outcomes were measured in the intervention group at least three time points before the intervention and after the intervention.

#### 4.1.2 Types of participants

We included studies targeting children under 5 years of age with MAM and SAM in low‐ and middle‐income countries (LMIC). Studies including both eligible and non‐eligible participants were included only if the results for the eligible participant subgroup was separately provided in the study. We used the following definition of MAM and SAM by WHO (WHO. 2013):

- SAM: WHZ < −3 *SD*, WFH < 70% of the median National Center for Health Statistics (NCHS) or WHO reference or MUAC < 115 mm or oedema. Complicated SAM: SAM cases without appetite and/or with medical complications. Uncomplicated SAM:SAM children with successful standard appetite test, without fever, clinical infections or complications.
- MAM: WHZ between −2 and −3 *SD*, WFH equal to 70–80% of the NCHS or WHO reference median or MUAC of 115–125 mm.

We excluded studies conducted on HIV populations specifically.

#### 4.1.3 Types of interventions

The following interventions were considered and compared against the suggested comparison groups separately:

- Community‐based strategies to screen, identify and manage SAM and MAM compared with no community‐based strategies [e.g., active community‐based surveillance by CHWs vs. no active surveillance; training of CHWs for community‐based screening vs. no training; community‐based management with RUTF vs. standard care practices].
- Facility‐based strategies to screen and manage uncomplicated SAM according to the WHO protocol compared with other standards of care (e.g., treatment for uncomplicated SAM in health facilities alone vs. by CHWs and health facilities; training of health‐facility staff to diagnose and treat uncomplicated SAM vs. no training; facility‐based management of SAM according to the WHO protocol vs. other/locally adapted protocols).
- Community‐based management of children with uncomplicated SAM as outpatients with RUTF compared with standard diet, fortified blended flours (FBFs) or other locally produced foods
- RUSF for MAM compared with standard diet, or FBF or other locally produced foods.
- Prophylactic use of antibiotics in children with uncomplicated SAM compared with no antibiotics.
- Vitamin A supplementation in the management of SAM and MAM with various doses and frequency of administration.

#### 4.1.4 Types of outcome measures

We did not use the outcomes listed below as criteria for including studies but rather as a list of the outcomes of interest. We used denominators for the outcomes according to the intention to treat analysis to avoid misleading results.

##### Primary outcomes

- Recovery rate (measured as the number of malnourished children recovered divided by the total number of malnourished children).
- Weight gain (measured as g·kg^−1^·day^−1^).
- Relapse (measured as the proportion of children who re‐enroled after they had recovered).
- Mortality (measured as the proportion of children dying under 5 years of age).
- Case fatality rates (measured as proportion of malnourished children dying divided by the total malnourished children).

##### Secondary outcomes

- Height gain.
- MUAC gain.
- Time to recover (measured as length of time between admission and discharge).
- Stunting (defined as below −2 *SD*s from median height for age of reference population).
- Wasting (defined as below −2 *SD*s from median weight for height of reference population).
- Underweight (defined as below −2 *SD*s from median weight for age of reference population).
- Infection incidence (bacteraemia, sepsis, pneumonia, urinary tract infections, meningitis and diarrhoea).
- Adverse effects (such as side effects associated with antibiotics, drug resistance, rapid weight gain, micronutrient toxicity, etc.).
- Costs and cost‐effectiveness.
- Hospitalisation.

##### Duration of follow‐up

We attempted to standardise the effect sizes from the included studies and reported the outcomes at the longest follow‐up reported.

##### Type of settings

We included studies conducted in community or facility‐based settings in LMICs as defined by the World Bank criteria.

### 4.2 Search methods for identification of studies

#### 4.2.1 Electronic searches

We searched the following databases till 11 February 2019: Cochrane Database of Systematic Reviews (CDSR) and the Cochrane Central Register of Controlled Trials (CENTRAL) in the Cochrane Library; World Health Organization regional databases; The Campbell Library; MEDLINE (PubMed); EMBASE; CINAHL; Web of Science; POPLINE; CAB abstracts and Global Health; PAHO; IndMED (indmed.nic.in/indmed.html and WHO Global Health Index. We also searched the WHO International Clinical Trials Registry Platform (ICTRP; http://www.who.int/ictrp/en/); ClinicalTrials.gov and Epistemonikos (https://www.epistemonikos.org)/. We did not restrict our searches by date, language or publication status.

#### 4.2.2 Searching other resources

We contacted experts in relevant fields for identification of eligible studies for inclusion. We also went through the references of identified studies and relevant reviews. We also ran citation searches of included studies in Google Scholar and Web of Sciences for other potentially relevant papers.

### 4.3 Data collection and analysis

#### 4.3.1 Selection of studies

Two reviewers independently assessed relevant studies by screening the titles and abstracts for inclusion. The selected studies underwent full‐text evaluation and were assessed for eligibility based on predefined eligibility criteria. Disagreements about appropriateness of the inclusion of studies were resolved by discussion between all review authors. Studies that meet the inclusion criteria on full‐text screening but upon further investigation became ineligible were added to the “characteristics of excluded studies” table, along with the reasons for their exclusion. We also planned to contact the study authors regarding eligibility for studies where eligibility was unclear.

#### 4.3.2 Data extraction and management

Two review authors independently extracted data on a predefined and pre‐tested data extraction sheet. We extracted the following information, where available, from relevant studies and any discrepancies were resolved by group discussion.

**Study method:**

- Study dates
- Location (country, urban/rural)
- Study design
- Method of recruitment
- Study context and settings

**Participants:**

- Sample size
- Age
- Gender
- Socioeconomic status
- Inclusion and exclusion criteria

**Intervention:**

- Micronutrients and vitamin A supplementation (doses and timing)
- Antibiotics (type and doses)
- Community‐based screening and management of malnutrition (as outpatients either at home by a health‐care worker, or in a community day‐care centre, residential nutrition centre or at a primary health clinic)
- Facility‐based screening and management of malnutrition
- Type of RUTF
- Type of supplementary feeding

**Comparison group:**

- No intervention or placebo or standard practice or other treatment.
- Type of supplementary food (RUTF, RUSF, fortified blended foods, other)

**Outcomes**

- Primary and secondary outcomes, as outlined in the types of outcome measure section. We used denominators for the outcomes according to the intention to treat analysis to avoid misleading results

**Quality assessment**

- On all Cochrane “Risk of bias” assessment tool indicators

#### 4.3.3 Assessment of risk of bias in included studies

Two review authors independently assessed methodological quality of studies and any disagreements were resolved by discussion among all review authors. The Cochrane “Risk of bias” assessment tool (Higgins & Green, 2011) was used for RCTs and quasi‐experimental (natural experiment) studies. We rated each of the following components as either “low risk”, “high risk” or “unclear risk” and provided justifications for the judgements:

1. Selection bias (due to inadequate generation of a randomised sequence or concealment of allocations prior to assignment)
2. Performance bias (blinding of participants and personnel assessment)
3. Detection bias (blinding of outcome assessment)
4. Attrition bias (incomplete outcome data)
5. Reporting bias (selective reporting)
6. Other bias

For CBA studies, we planned to use the Cochrane Effective Practice and Organisation of Care (EPOC) guidelines based on the following criteria (Cochrane Effective Practice and Organisation of Care [EPOC], 2017). We planned to rate each of the following components as either “low risk”, “high risk” or “unclear risk” and provide justifications for the judgements:

- Baseline outcome measurements similar
- Baseline characteristics similar
- Incomplete outcome data
- Knowledge of the allocated interventions adequately prevented during study (refers to blinding of participants and personnel and blinding of outcome assessment)
- Protection against contamination
- Selective outcome reporting
- Other risks of bias (e.g., bias in measurement: validity and reliability of the measures used)

For ITS studies, the following criteria from EPOC was considered (EPOC, 2017). We planned to rate each of the following components as either “low risk”, “high risk” or “unclear risk” and provide justifications for the judgements:

- Intervention independent of other changes
- Shape of intervention effect pre‐specified
- Intervention unlikely to affect data collection
- Knowledge of the allocated interventions adequately prevented during study (refers to the blinding of outcome assessment)
- Incomplete outcome data
- Selective outcome reporting
- Other risks of bias (e.g., bias in measurement: validity and reliability of the measures used; duration of observation and use of appropriate statistical modelling technique)

Since all the included studies were either RCTs or quasi‐experimental studies; we only used the Cochrane “Risk of bias” assessment tool. For future updates, we will use the aforementioned criteria for CBA and ITS studies.

#### 4.3.4 Measures of treatment effect

We separately analysed the dichotomous and continuous outcomes. For dichotomous outcomes, we presented the results as summary risk ratios (RRs) with 95% confidence intervals (CI). We combined incidence data as RRs (events per child) and rate ratios (events per child year) because of their similar interpretation and scale. We presented continuous outcome data as either a mean difference (MD), if outcomes have been measured on the same scale, or a standardised mean difference, if outcomes have been measured on different scales, with 95% CI. If outcomes were reported at multiple time points in the included studies, we reported the outcomes at the last reported time period, unless other time point points were relevant for the subgroup analysis. For studies reporting outcomes at multiple time points, we reported the last outcome reported at last follow‐up. We planned to conduct subgroup analysis for outcomes reported at different time periods.

#### 4.3.5 Unit of analysis issues

We conducted separate meta‐analysis for different study designs, that is, RCTs (both individual and cluster) and CBA/ITS; and for subcategories of interventions and outcomes. For cluster RCTs, we planned to contact trial authors for an estimate of the intra‐cluster correlation coefficient if the clustering effect was not accounted for in the analysis, If we were unable to contact the trial author we planned to calculate an interclass correlation coefficient based on the other studies in the review and use the variance inflation factor to adjust the standard errors appropriately. Subsequently, effect sizes and standard errors were meta‐analysed by using the generic inverse method in REVMan (RevMan, 2014). If there were multiple papers that describe the same trial, these were combined and coded as a single study. For trials that included multiple intervention arms, we selected one pair (intervention and control) that satisfied the inclusion criteria of the review and excluded the rest. If >2 intervention groups met the eligibility criteria, then these groups were combined into a single pair‐wise comparison group and data were disaggregated into corresponding subgroups, or these arms were separated into different forest plots to ensure that there was no double counting of participants. Multiple outcome estimates within the same study were analysed separately.

#### 4.3.6 Dealing with missing data

We reported the missing data or dropouts along with the reasons. We planned to contact the study authors if the missing data was not accounted for or the reasons for dropping out were unclear. If authors have accounted for missing data (i.e., multiple imputations), we used the adjusted data within our analysis.

#### 4.3.7 Assessment of heterogeneity

Statistical heterogeneity was assessed using *τ* ^2^, *I* ^2^ and significance of the *χ* ^2^ test; we also assessed heterogeneity visually using forest plots. Based on prior theory and clinical knowledge, we expected clinical and methodological heterogeneity in effect sizes in this literature. Therefore, we attempted to explain any observed statistical heterogeneity using subgroup analysis (see Section 4.3.10).

#### 4.3.8 Assessment of reporting biases

If the number of studies were sufficient (>10), we planned to use a funnel plot to visually inspect for publication bias. In addition, we performed Egger's test to determine funnel plot asymmetry.

#### 4.3.9 Data synthesis

Statistical analysis was carried out separately for each intervention using Review Manager 5.3 (RevMan, 2014). Separate meta‐analyses were conducted for each type of intervention and comparison group and study design. Where analysis had not been ideal in the original papers, we attempted to reconstruct if the data presented allowed us to. Considering the expected heterogeneity in interventions, comparisons, outcomes and settings within the included studies, we used random effects meta‐analyses. Where meta‐analysis was deemed inappropriate due to substantial statistical or clinical heterogeneity between studies, the findings of the included studies were summarised in narrative form. In cases where we included multiple groups from one study, we combined all relevant experimental intervention groups of the study into a single group, and combined all relevant control intervention groups into a single control group or included each pair‐wise comparison separately, but with shared intervention groups divided out approximately evenly among the comparisons to avoid double counts (Higgins & Green, 2011). We checked for the accuracy of the numeric data by comparing the magnitude and direction of effects reported by studies with how these were presented in the review,

We set out the main findings of the review for the primary outcomes in “Summary of findings” tables prepared via the GRADE approach (Guyatt et al., 2008) with GRADEpro 2014. We listed the primary outcome for each comparison with estimates of relative effects along with the numbers of participants and studies contributing data for those outcomes. For each primary outcome, we assessed the quality of the evidence using the GRADE approach, which involved consideration of within‐study risk of bias (methodological quality), directness of evidence, heterogeneity, precision of effect estimates and risk of publication bias. We rated the quality of the body of evidence for each key outcome as “high”, “moderate”, “low” or “very low”. Randomised trials without important limitations provide high‐quality evidence, while observational studies without special strengths or important limitations provide low‐quality evidence. Non‐randomised experimental trials (quasi‐RCTs) without important limitations also provide high‐quality evidence, but are automatically downgraded for limitations in design (risk of bias), such as lack of concealment of allocation

There are five criteria that can downgrade evidence for RCTs and quasi‐RCTs (Guyatt et al., 2008):

- Risk of bias in individual studies
- Indirectness of evidence
- Unexplained heterogeneity or inconsistency of results
- Imprecision of results
- High probably of publication bias

There are three criteria that can upgrade the evidence for quasi‐experimental studies with no serious methodological limitations (Guyatt et al., 2008):

- Large magnitude of effect
- Presence of a dose response relationships
- Effect of plausible residual confounding

#### 4.3.10 Subgroup analysis and investigation of heterogeneity

Depending on data availability, we planned conduct exploratory subgroup analyses for the following subgroups:

- Age (1–6 months, 6–59 months)
- Duration of intervention (short‐term (<3 months), medium‐term (3–6 months), and long‐term (6–12 months))
- Various formulations of supplementary foods
- Setting (community management, primary care management and facility management)
- Vitamin A supplementation dosage (different doses)
- Different antibiotics
- Equity (low income and disadvantaged groups vs. relatively high‐income groups)

We planned to use the *χ* ^2^ test to assess subgroup differences.

Due to the limited number of studies, we could not conduct the planned subgroup analysis; however, we have separately analysed the various supplementary foods that were compared with standard RUTF and standard RUSF.

#### 4.3.11 Sensitivity analysis

We conducted sensitivity analysis based on the risk of bias of the included studies by removing studies judged to be at high risk of bias for sequence generation, allocation concealment and blinding of participants from the meta‐analysis to determine whether the removal of studies with high risk of bias impacts the estimates.

## 5 RESULTS

### 5.1 Description of studies

#### 5.1.1 Results of the search

**Figure 1:**
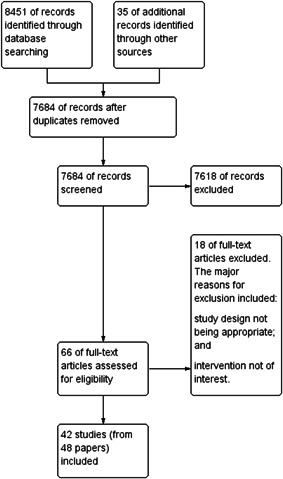
Study flow diagram

Our searches identified a total of 8,451 potentially relevant titles from the electronic searches and 35 records from searching other sources. After removing duplicates, we screened 7,684 records for eligibility and excluded 7,618 on the basis of title and abstract. We obtained the full‐text reports of the remaining 66 records, and of these, excluded 18 studies and included 42 studies (48 papers). Figure 1 depicts the search flow diagram.

#### 5.1.2 Included studies

We included a total of 42 studies (from 48 papers) including 35,017 children (Ackatia‐Armah et al., 2015; Ashworth, Huttly, & Khanum, 1994; Bahwere et al., 2014, 2016, 2017; Berkley et al., 2016; Bhandari et al., 2016; Chapko, Prual, Gamatie, & Maazou, 1994; Ciliberto et al., 2005; Diop, Dossou, Ndour, Briend, & Wade, 2003; Donnen et al., 1998; Fabiansen et al., 2017; Hossain et al., 2009; Hsieh et al., 2015; Irena et al., 2015; Isanaka et al., 2016; Jones et al., 2015; Karakochuk et al., 2012; LaGrone et al., 2012; Manary, Maleta, & Trehan, 2012; Manary, Ndkeha, Ashorn, Maleta, & Briend, 2004; Matilsky, Maleta, Castleman, & Manary, 2009; Maust et al., 2015; Medoua et al., 2015; Mishra, Rai, Swain, & Behera, 2019; Nackers et al., 2010; Nikièma et al., 2014; Oakley et al., 2010; Phuka et al., 2009; Puett et al., 2012; Sandige, Ndekha, Briend, Ashorn, & Manary, 2004; Sattar et al., 2012; Scherbaum et al., 2015; Shewade et al., 2013; Sigh et al., 2018; Singh et al., 2010; Stobaugh et al., 2016; Thakur, Singh, & Patel, 2013; Thakwalakwa et al., 2010; Vanelli et al., 2014; Versloot et al., 2017; Wilford, Golden, & Walker, 2011).

Thirty‐three of the included studies were RCTs (Ackatia‐Armah et al., 2015; Bahwere et al., 2014, 2016, 2017; Berkley et al., 2016; Bhandari et al., 2016; Chapko et al., 1994; Diop, Dossou et al., 2003; Donnen et al., 1998; Fabiansen et al., 2017; Hsieh et al., 2015; Irena et al., 2015; Isanaka et al., 2016; Jones et al., 2015; Karakochuk et al., 2012; LaGrone et al., 2012; Manary et al., 2012; Matilsky et al., 2009; Maust et al., 2015; Medoua et al., 2015; Mishra et al., 2019; Nackers et al., 2010; Nikièma et al., 2014; Oakley et al., 2010; Phuka et al., 2009; Sattar et al., 2012; Shewade et al., 2013; Sigh et al., 2018; Singh et al., 2010; Stobaugh et al., 2016; Thakwalakwa et al., 2010; Vanelli et al., 2014; Versloot et al., 2017); six studies were quasi‐experimental studies (Ciliberto et al., 2005; Hossain et al., 2009; Manary et al., 2004; Sandige et al., 2004; Scherbaum et al., 2015; Thakur et al., 2013) while three of the included studies (Ashworth et al., 1994; Puett et al., 2012; Wilford et al., 2011) were cost‐effectiveness studies.

Four of the included RCTs were cluster RCTs (cRCTs) (Ackatia‐Armah et al., 2015; Irena et al., 2015; Karakochuk et al., 2012; Maust et al., 2015) while others were individually randomised trials. All the cRCTS were adequately adjusted for the effect of clustering.

See “Characteristics of included studies” tables.

##### Setting

All the studies were conducted in either community, hospital, health centre or nutrition rehabilitation centres in LMICs including Bangladesh, Mali, Malawi, Congo, Kenya, India, Niger, Senegal, Sudan, Burkina Faso, Zambia, Ethiopia, Sierra Leonne, Cameroon, Indonesia and Cambodia.

##### Participants

Almost all the included studies targeted children aged 6–60 months; except a few: Thakwalakwa et al. (2010) targeted children 6–15 months of age; Phuka et al. (2009) targeted children 6–18 months of age; Fabiansen et al. (2017), Nikièma et al. (2014) targeted children 6–23 months of age; Chapko et al. (1994) targeted children 5–28 months of age; Berkley et al. (2016) targeted children 2–59 months; while Dossou et al. (2003) and Ackatia‐Armah et al. (2015) targeted children 6–36 months of age.

##### Intervention

Two studies (Maust et al., 2015; Wilford et al., 2011) assessed community‐based strategies. Maust et al. (2015) compared an integrated community‐based protocol to manage MAM and SAM with no community‐based management while Wilford et al. (2011) compared the cost‐effectiveness of existing health services with CMAM to the existing health services without CMAM.

Seven studies (Ashworth et al., 1994; Chapko et al., 1994; Hossain et al., 2009; Mishra et al., 2019; Puett et al., 2012; Thakur et al., 2013; Versloot et al., 2017) assessed facility‐based strategies compared with other standard of care. Ashworth et al. (1994) compared inpatient treatment with day‐care or at‐home care; while Hossain et al. (2009) compared a locally adapted protocol with the WHO protocol for the management of SAM. Three studies (Ashworth et al., 1994; Chapko et al., 1994; Puett et al., 2012) assessed cost‐effectiveness of in‐patient rehabilitation compared with outpatient or community‐based management.

Fourteen studies (Bahwere et al., 2014, 2016, 2017; Bhandari et al., 2016; Ciliberto et al., 2005; Dossou et al., 2003; Hsieh et al., 2015; Irena et al., 2015; Jones et al., 2015; Manary et al., 2004; Oakley et al., 2010; Sandige et al., 2004; Shewade et al., 2013; Sigh et al., 2018) compared community‐based management of children with uncomplicated SAM with RUTF versus other foods. Other foods included non‐dairy/reduced dairy‐based RUTF, non‐peanut butter‐based RUTF, energy dense home made food, CSB and F100.

Fourteen studies (Ackatia‐Armah et al., 2015; Fabiansen et al., 2017; Karakochuk et al., 2012; LaGrone et al., 2012; Matilsky et al., 2009; Medoua et al., 2015; Nackers et al., 2010; Nikièma et al., 2014; Phuka et al., 2009; Scherbaum et al., 2015; Singh et al., 2010; Stobaugh et al., 2016; Thakwalakwa et al., 2010; Vanelli et al., 2014) compared RUSF for MAM with other foods. Other foods included non‐dairy/reduced dairy‐based RUTF, non‐peanut butter‐based RUTF, energy dense home made food, CSB and F100.

Three studies (Berkley et al., 2016; Isanaka et al., 2016; Manary et al., 2012) compared prophylactic use of antibiotics in children with uncomplicated SAM with no antibiotics. The antibiotics used for prophylaxis included co‐trimoxazole (Berkley et al., 2016), amoxicillin (Isanaka et al., 2016; Manary et al., 2012) and cefdinir (Manary et al., 2012).

Two studies (Donnen et al., 1998; Sattar et al., 2012) compared high‐dose vitamin A supplement with low‐dose vitamin A supplement.

##### Outcome

Among primary outcomes, included studies reported recovery rate, weight gain, relapse and mortality. None of the included studies reported case fatality rates.

Among secondary outcome, included studies reported height gain, MUAC gain, time to recover, stunting, wasting, underweight, adverse events, cost‐effectiveness and hospitalisations.

#### 5.1.3 Excluded studies

A total of 18 studies were excluded (Agha 2004; Aguayo et al., 2018; Ahmed et al., 1999; Ashworth et al., 1994; Bachou, Tumwine, Mwadime, Ahmed, & Tylleskar, 2008; Badaloo et al., 1999; Baker, Baker, Margo, & Reuter, 1978; Bhandari et al., 2001; Burza et al., 2016; Donnen et al., 2007; Dubray et al., 2008; Javan, Kooshki, Afzalaghaee, Aldaghi, & Yousefi, 2017; Linneman et al., 2007; Nagar, Nagar, & Gupta, 2016; Roy et al., 2005; Simpore et al., 2006; Zongo, Zoungrana, Savadogo, & Traoré, 2013). The major reasons for exclusion included study design not being appropriate and intervention not of interest. Please see “Characteristics of excluded studies” section.

### 5.2 Risk of bias in included studies

**Figure 2:**
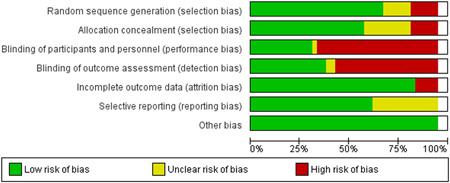
Risk of bias graph: Review authors' judgements about each risk of bias item presented as percentages across all included studies

**Figure 3:**
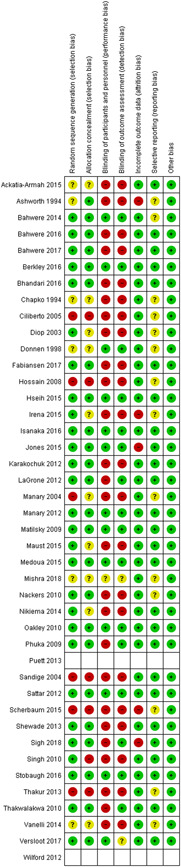
Risk of bias summary: Review authors' judgements about each risk of bias item for each included study

All the studies (except Puett et al., 2012; Wilford et al., 2011), were either RCTs or quasi‐experimental studies and were assessed for risk of bias using the Cochrane risk of bias tool. Puett et al. (2012) and Wilford et al. (2011) were cost‐effectiveness studies. Overall, the studies were judged to be at high risk of bias for blinding of participants and personnel and outcome assessment blinding. For further details, refer to the risk of bias tables under “Characteristics of included studies” section and Figures 2 and 3.

#### 5.2.1 Allocation (selection bias)

Twenty‐eight studies were judged to be at low risk of bias for sequence generation since adequate methods were used to generate random sequence (Bahwere et al., 2014, 2016, 2017; Berkley et al., 2016; Bhandari et al., 2016; Dossou et al., 2003; Fabiansen et al., 2017; Hsieh et al., 2015; Irena et al., 2015; Isanaka et al., 2016; Jones et al., 2015; Karakochuk et al., 2012; LaGrone et al., 2012; Manary et al., 2012; Matilsky et al., 2009; Maust et al., 2015; Medoua et al., 2015; Nackers et al., 2010; Nikièma et al., 2014; Oakley et al., 2010; Phuka et al., 2009; Sattar et al., 2012; Shewade et al., 2013; Sigh et al., 2018; Singh et al., 2010; Stobaugh et al., 2016; Thakwalakwa et al., 2010; Versloot et al., 2017). Six studies were judged to be at unclear risk for sequence generation since they did not provide sufficient information regarding sequence generation methods (Ackatia‐Armah et al., 2015; Ashworth et al., 1994; Chapko et al., 1994; Donnen et al., 1998; Mishra et al., 2019; Vanelli et al., 2014). Six studies were judged to be at high risk of sequence generation; all these studies were quasi‐experimental studies (Ciliberto et al., 2005; Hossain et al., 2009; Manary et al., 2004; Sandige et al., 2004; Scherbaum et al., 2015; Thakur et al., 2013).

Twenty four studies were judged to be at low risk of bias for allocation concealment and used adequate methods to conceal the random assignment (Ashworth et al., 1994; Bahwere et al., 2014, 2016, 2017; Berkley et al., 2016; Bhandari et al., 2016; Fabiansen et al., 2017; Hsieh et al., 2015; Isanaka et al., 2016; Jones et al., 2015; Karakochuk et al., 2012; LaGrone et al., 2012; Manary et al., 2012; Matilsky et al., 2009; Medoua et al., 2015; Nackers et al., 2010; Oakley et al., 2010; Phuka et al., 2009; Sattar et al., 2012; Shewade et al., 2013; Sigh et al., 2018; Stobaugh et al., 2016; Thakwalakwa et al., 2010; Versloot et al., 2017). Ten studies did not provide sufficient information regarding methods used to conceal allocation and were judged to be at unclear risk of bias (Ackatia‐Armah et al., 2015; Chapko et al., 1994; Donnen et al., 1998; Dossou et al., 2003; Irena et al., 2015; Manary et al., 2004; Maust et al., 2015; Mishra et al., 2019; Nikièma et al., 2014; Vanelli et al., 2014). Six studies were judged to be at high risk of bias for allocation concealment due to inadequate methods used to conceal the random allocation (Ciliberto et al., 2005; Hossain et al., 2009; Sandige et al., 2004; Scherbaum et al., 2015; Singh et al., 2010; Thakur et al., 2013).

#### 5.2.2 Blinding (performance bias and detection bias)

Thirteen studies were judged to be at low risk of bias for blinding of participants and personnel (Bahwere et al., 2014; Berkley et al., 2016; Donnen et al., 1998; Hsieh et al., 2015; Isanaka et al., 2016; Jones et al., 2015; Manary et al., 2012; Matilsky et al., 2009; Medoua et al., 2015; Oakley et al., 2010; Sattar et al., 2012; Stobaugh et al., 2016; Versloot et al., 2017). Mishra et al. (2019) provided insufficient information regarding blinding of participants and personnel and was judged to be at unclear risk of bias. Majority of the studies (twenty‐six studies: Ackatia‐Armah et al., 2015; Ashworth et al., 1994; Bahwere et al., 2016, 2017; Bhandari et al., 2016; Chapko et al., 1994; Ciliberto et al., 2005; Dossou et al., 2003; Fabiansen et al., 2017; Hossain et al., 2009; Irena et al., 2015; Karakochuk et al., 2012; LaGrone et al., 2012; Manary et al., 2004; Maust et al., 2015; Nackers et al., 2010; Nikièma et al., 2014; Phuka et al., 2009; Sandige et al., 2004; Scherbaum et al., 2015; Shewade et al., 2013; Sigh et al., 2018; Singh et al., 2010; Thakur et al., 2013; Thakwalakwa et al., 2010; Vanelli et al., 2014) were judged to be at high risk of bias for inadequate blinding of the participants and personnel mainly due to the nature of intervention.

Sixteen studies were judged to be at low risk of bias for blinding of outcome assessment (Bahwere et al., 2014; Berkley et al., 2016; Donnen et al., 1998; Hsieh et al., 2015; Isanaka et al., 2016; Jones et al., 2015; LaGrone et al., 2012; Manary et al., 2012; Matilsky et al., 2009; Medoua et al., 2015; Oakley et al., 2010; Phuka et al., 2009; Sattar et al., 2012; Sigh et al., 2018; Stobaugh et al., 2016; Thakwalakwa et al., 2010). Mishra et al. 2019; and Versloot et al. 2017 provided insufficient information regarding blinding of outcome assessment and were judged to be at unclear risk of bias. Majority of the studies (twenty‐two studies: Ackatia‐Armah et al., 2015; Ashworth et al., 1994; Bahwere et al., 2016, 2017; Bhandari et al., 2016; Chapko et al., 1994; Ciliberto et al., 2005; Dossou et al., 2003; Fabiansen et al., 2017; Hossain et al., 2009; Irena et al., 2015; Karakochuk et al., 2012; Manary et al., 2004; Maust et al., 2015; Nackers et al., 2010; Nikièma et al., 2014; Sandige et al., 2004; Scherbaum et al., 2015; Shewade et al., 2013; Singh et al., 2010; Thakur et al., 2013; Vanelli et al., 2014) were judged to be at high risk of bias for inadequate blinding of the outcome assessment mainly due to the nature of intervention.

#### 5.2.3 Incomplete outcome data (attrition bias)

Thiry five studies were judged to be at low risk of bias for attrition (Ackatia‐Armah et al., 2015; Bahwere et al., 2014, 2016, 2017; Berkley et al., 2016; Bhandari et al., 2016; Chapko et al., 1994; Ciliberto et al., 2005; Donnen et al., 1998; Dossou et al., 2003; Fabiansen et al., 2017; Hossain et al., 2009; Hsieh et al., 2015; Isanaka et al., 2016; Karakochuk et al., 2012; LaGrone et al., 2012; Manary et al., 2004, 2012; Matilsky et al., 2009; Maust et al., 2015; Medoua et al., 2015; Mishra et al., 2019; Nackers et al., 2010; Nikièma et al., 2014; Oakley et al., 2010; Phuka et al., 2009; Sandige et al., 2004; Sattar et al., 2012; Shewade et al., 2013; Singh et al., 2010; Stobaugh et al., 2016; Thakur et al., 2013; Thakwalakwa et al., 2010; Vanelli et al., 2014; Versloot et al., 2017); while five studies (Ashworth et al., 1994; Irena et al., 2015; Jones et al., 2015; Scherbaum et al., 2015; Sigh et al., 2018) were judged to be at high risk of bias since >30% of participants were lost to follow‐up.

#### 5.2.4 Selective reporting (reporting bias)

Fourteen studies (Ashworth et al., 1994; Bahwere et al., 2014; Chapko et al., 1994; Ciliberto et al., 2005; Donnen et al., 1998; Dossou et al., 2003; Hossain et al., 2009; Irena et al., 2015; Manary et al., 2004; Mishra et al., 2019; Nackers et al., 2010; Scherbaum et al., 2015; Thakur et al., 2013; Vanelli et al., 2014) were judged to be at unclear risk of selective reporting since there was no information on trial registration or published protocols while all other studies were judged to be at low risk of bias for selective reporting.

#### 5.2.5 Other potential sources of bias

We did not find any other potential sources of bias in any of the included studies.

### 5.3 Effects of interventions

#### 5.3.1 Comparison 1: Community‐based strategies to screen, identify and manage SAM and MAM compared with no community‐based strategies

Two studies (Maust et al., 2015; Wilford et al., 2011) assessed community‐based strategies. Maust et al. (2015) compared an integrated community‐based protocol to manage MAM and SAM with no community‐based management which comprised of no community‐based surveillance, while Wilford et al. (2011) compared the cost‐effectiveness of existing health services with CMAM to the existing health services without CMAM. We could not conduct meta‐analysis for this comparison.

##### Primary outcomes

Among primary outcomes, included studies in this comparison reported recovery, weight gain and mortality. Inlcuded studies in this comparison did not report relapse and case fatality.

###### Recovery rate: Single study result

One study (Maust et al., 2015) reported recovery rate at 12 weeks suggesting that the integrated community‐based management probably improves recovery by 4% when compared with no community‐based management (RR: 1.04; 95% CI: 1.00 to 1.09; one study; 1,957 participants; moderate‐quality outcome; Analysis 1.1).

###### Weight gain: Single study result

One study (Maust et al., 2015) reported weight gain at 4 weeks suggesting that the integrated community‐based management probably decreases weight gain by 0.8 g·kg^−1^·day^−1^ compared with no community‐based management (MD: −0.80 g·kg^−1^·day^−1^; 95% CI: −0.82 to −0.78; one study; 1,957 participants; moderate‐quality outcome; Analysis 1.2).

###### Mortality: Single study result

One study (Maust et al., 2015) reported mortality at 12 weeks suggesting that mortality was similar between the integrated community‐based management group and no community‐based management group (RR: 0.93; 95% CI: 0.60 to 1.45; one study; 1957 participants; moderate outcome quality; Analysis 1.3).

##### Secondary outcomes

Among secondary outcomes, included studies in this comparison reported length gain, MUAC gain, adverse events and cost. None of the included studies in this comparison reported time to recover, stunting, wasting and underweight.

###### Length gain: Single study result

One study (Maust et al., 2015) reported length gain suggesting that the integrated community‐based management probably decreases length gain by 0.1 mm/day compared with no community‐based management (MD: −0.10 mm/day; 95% CI: −0.10 to −0.10; one study; 1,957 participants; moderate‐quality outcome; Analysis 1.4).

###### MUAC gain: Single study result

One study (Maust et al., 2015) reported MUAC gain suggesting that the integrated community‐based management probably improves MUAC by 0.27 mm/day compared with no community‐based management (MD: 0.27 mm/day; 95% CI: 0.27 to 0.27; one study; 1,957 participants; moderate‐quality outcome; Analysis 1.5).

###### Adverse events: Single study result

One study (Maust et al., 2015) reported diarrhoea and fever as adverse events suggesting that the integrated community‐based management probably reduces diarrhoea by 29% (RR: 0.71; 95% CI: 0.60 to 0.85; one study; 1,957 participants; moderate‐quality outcome; Analysis 1.6) and fever by 15% (RR: 0.85; 95% CI: 0.77 to 0.93; one study; 1,957 participants; moderate‐quality outcome; Analysis 1.6) compared with no community‐based management during the first 2 weeks of feeding.

###### Cost and cost‐effectiveness: Single study result

Two studies (Maust et al., 2015; Wilford et al., 2011) reported cost and cost‐effectiveness. Maust et al. (2015) reported that the cost of RUTF used to treat a SAM case in integrated community‐based management was $36, whereas for the no community‐based management group was $68; while the cost of supplementary food used to treat a case of MAM in either of the groups was $12. The study did not report a comparison of the cost‐effectiveness of the two management strategies because the costs of care were not documented. Wilford et al. (2011) assessed the cost‐effectiveness of the existing health services with CMAM compared with the existing health services without CMAM. The study reported that the CMAM was highly cost‐effective in Malawi; however, the study recommended that several contextual and programmatic factors should be considered when generalising to diverse contexts.

#### 5.3.2 Comparison 2: Facility‐based strategies to screen and manage uncomplicated SAM according to the WHO protocol compared with other standards of care

Three studies (Ashworth et al., 1994; Chapko et al., 1994; Puett et al., 2012) assessed facility‐based strategies compared with other standards of care which was out‐patient and community‐based management for uncomplicated SAM while one study Hossain et al. (2009) compared the facility‐based management with a locally adapted Institute of Child and Mother Health (ICMH) protocol. We could not pool findings from Hossain et al. 2009 since the comparison group was different from other studies; however, we have reported the findings from this study under each outcome reported.

Findings from Ashworth et al. (1994) and Chapko et al. (1994) should be interpreted with a consideration that these studies were conducted before the current differentiation of complicated and uncomplicated SAM.

##### Primary outcomes

Among primary outcomes, included studies in this comparison reported recovery and mortality. None of the included studies reported weight gain, relapse and case fatality.

###### Recovery rate: Single study result

One study (Hossain et al., 2009) reported recovery at 4–6 weeks suggesting no evidence of effect on recovery (RR: 1.00; 95% CI: 0.80, 1.25; one study; 60 participants; very‐low‐quality evidence; Analysis 2.1).

###### Mortality: Pooled study results

Two studies reported mortality at 4–6 weeks and found no evidence of effect on mortality (RR: 1.21; 95% CI: 0.75, 1.94; two studies; 473 participants; *I* ^2^: 0%; low‐quality evidence; Analysis 2.2).

Hossain et al. (2009) reported similar mortality in the WHO protocol group (2 out of 30) and ICMH protocol group (2 out of 30).

##### Secondary outcome

Among secondary outcomes, included studies reported cost‐effectiveness. None of the included studies reported any of the other pre‐specified secondary outcomes including height gain, MUAC gain, time to recover, stunting, wasting, underweight and adverse events.

###### Cost‐effectiveness: Single study results

Ashworth et al. (1994) reported the cost‐effectiveness of three approaches (inpatient, daycare or domiciliary care after 1 week of day care) for the management of severely malnourished children. Findings suggest that the average institutional costs to achieve 80% WFH was $156 for the inpatient; $59 for daycare and $29 for domiciliary care. The study reported that domiciliary care after 1 week of day care was the most cost‐effective treatment option.

Chapko et al. (1994) compared costs between patients assigned to hospital rehabilitation with ambulatory care and findings suggest that children assigned to in‐patient rehabilitation received significantly more days of hospital care and fewer days of ambulatory care when compared with patients assigned to ambulatory rehabilitation. Moreover, the study reported that the total cost of rehabilitation was significantly higher for hospital rehabilitation.

Puett et al. (2012) assessed the cost‐effectiveness of adding CMAM to a community‐based health and nutrition programme delivered by CHWs in southern Bangladesh. The cost‐effectiveness of this model of treatment for SAM was compared with the cost‐effectiveness of the “standard of care” for SAM (i.e., inpatient treatment), augmented with community surveillance by CHWs to detect cases, in a neighbouring area. Findings suggest that CMAM delivered by CHWs is a cost‐effective strategy compared with inpatient treatment, and compares well with the cost‐effectiveness of other common child survival interventions.

#### 5.3.3 Comparison 3: Facility‐based strategies to screen and manage uncomplicated SAM according to the WHO protocol compared with other standards of care (in‐patient treatment with RUTF compared with F100)

Three studies (Mishra et al., 2019; Thakur et al., 2013; Versloot et al., 2017) assessed in‐patient management of SAM with RUTF compared with F100.

##### Primary outcomes

Among primary outcomes, included studies reported weight gain and mortality. None of the included studies reported recovery rate, relapse and case fatality rates.

###### Weight gain: Pooled study results

Three studies (Mishra et al., 2019; Thakur et al., 2013; Versloot et al., 2017) reported weight gain at discharge from in‐patient treatment suggesting no evidence of effect on weight gain (MD: 2 g·kg^−1^·day^−1^; 95% CI: −0.23 to 4.23; three studies; 266 participants; *I* ^2^: 95%; very‐low‐quality outcome; Analysis 3.1) in facility‐based treatment with RUTF compared with F100.

We conducted sensitivity analysis by removing one study (Thakur et al., 2013) judged to be at high risk of bias for sequence generation, allocation concealment and blinding. The findings from sensitivity analysis also suggest no evidence of effect on weight gain (MD: 0.91 g·kg^−1^·day^−1^; 95% CI: −2.15, 3.97; two studies; 168 participants; *I* ^2^: 95%; very‐low‐quality outcome; Analysis 3.2).

###### Mortality: Pooled study results

Two studies (Mishra et al., 2019; Versloot et al., 2017) reported mortality till discharge from in‐patient treatment suggesting no difference between RUTF and F100 (RR: 1.20; 95% CI: 0.34 to 4.22; two studies; 168 participants; *I* ^2^: 16%; low‐quality outcome; Analysis 3.3).

##### Secondary outcomes

Among secondary outcomes, included studies reported height, MUAC and wasting. None of the included studies in this comparison reported other secondary outcomes including time to recover, stunting, underweight, adverse events and cost‐effectiveness.

###### Height: Single study result

One study (Mishra et al., 2019) reported height showing no difference between RUTF and F100 (MD: −0.59 mm/day; 95% CI: −3.91 to 2.73; one study; 120 participants; low‐quality outcome; Analysis 3.4).

###### MUAC: Single study result

One study (Mishra et al., 2019) reported MUAC showing no difference between RUTF and F100 (MD: −0.66 mm/day; 95% CI: −4.78 to 3.46; one study; 120 participants; low‐quality outcome; Analysis 3.5).

###### Wasting: Single study result

One study (Mishra et al., 2019) reported wasting suggesting no difference between RUTF and F100 (RR: 1.47; 95% CI: 0.85 to 2.54; one study; 120 participants; low‐quality outcome; Analysis 3.6).

#### 5.3.4 Comparison 4: Community‐based management of children with uncomplicated SAM as outpatients with RUTF compared with standard diet, FBFs or other locally produced foods

Fourteen studies (Bahwere et al., 2014, 2016, 2017; Bhandari et al., 2016; Ciliberto et al., 2005; Dossou et al., 2003; Hsieh et al., 2015; Irena et al., 2015; Jones et al., 2015; Manary et al., 2004; Oakley et al., 2010; Sandige et al., 2004; Shewade et al., 2013; Sigh et al., 2018) compared community‐based management of children with uncomplicated SAM with RUTF versus other foods. Standard milk/peanut butter‐based RUTF was compared with non‐milk/peanut butter‐based RUTF, reduced milk/peanut butter RUTF, F100, energy dense home made food, high oleic RUTF elevated *n*3 PUFA RUTF.

##### Primary outcomes

Among primary outcome, included studies reported recovery, weight gain and mortality. None of the included studies reported any of the other primary outcomes including relapse and case fatality rates.

###### Recovery rate: Pooled study results

Ten studies (Bahwere et al., 2014, 2016, 2017; Bhandari et al., 2016; Hsieh et al., 2015; Irena et al., 2015; Manary et al., 2004; Oakley et al., 2010; Sandige et al., 2004; Shewade et al., 2013) reported recovery rate at 8–16 weeks. There was no evidence of difference on recovery rate when standard RUTF was compared with non‐milk/peanut butter‐based RUTF (RR: 1.03; 95% CI: 0.99 to 1.08; five studies; 5,743 participants; *I* ^2^ 50%; moderate‐quality outcome; Analysis 4.1); energy dense home prepared food (RR: 1.14; 95% CI 0.95 to 1.36; four studies; 959 participants; *I* ^2^ 75%; low‐quality outcome; Analysis 4.1) or high oleic RUTF (RR: 1.06; 95% CI: 0.85 to 1.31; one study; 141 participants; moderate‐quality outcome; Analysis 4.1).

We conducted sensitivity analysis by removing one study (Sandige et al., 2004) judged to be at high risk for sequence generation, allocation concealment and blinding. Findings from sensitivity analysis suggest no evidence of effect on recovery rate when standard RUTF was compared with energy dense home prepared food (RR: 1.23; 95% CI: 0.99, 1.52; three studies; 777 participants; *I* ^2^ 51%; low‐quality outcome; Analysis 4.2)

###### Weight gain: Pooled study results

**Figure 4:**
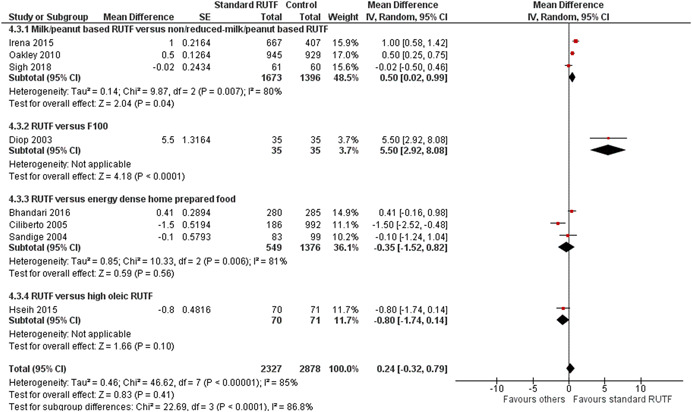
(Analysis 4.3) Forest plot of comparison 4: Community‐based management with standard RUTF compared with other foods, outcome: 4.2 Weight gain. CI, confidence interval; RUTF, ready‐to‐use therapeutic food; *SE*, standard error

Eight studies (Bhandari et al., 2016; Ciliberto et al., 2005; Dossou et al., 2003; Hsieh et al., 2015; Irena et al., 2015; Oakley et al., 2010; Sandige et al., 2004; Sigh et al., 2018) reported weight gain at 8–16 weeks (Figure 4). Standard RUTF may improve weight gain by 0.5 g·kg^−1^·day^−1^ when compared with non‐milk/peanut butter‐based RUTF (MD: 0.5 g·kg^−1^·day^−1^; 95% CI: 0.02 to 0.99; three studies; 3069 participants; *I* ^2^ 80%; low‐quality outcome; Analysis 4.3) and by 5.5 g·kg^−1^·day^−1^ when compared with F100 (MD: 5.50 g·kg^−1^·day^−1^; 95% CI: 2.92 to 8.08; one study; 70 participants; low‐quality outcome; Analysis 4.3). There was no evidence of effect on weight gain when standard RUTF was compared with energy dense home prepared food (MD: −0.35 g·kg^−1^·day^−1^; 95% CI: −1.52 to 0.82; three studies; 1,925 participants; I^2^ 81%; low‐quality outcome; Analysis 4.3) and high oleic RUTF (MD: −0.8 g·kg^−1^·day^−1^; 95% CI: −1.74 to 0.14; one study; 141 participants; moderate‐quality outcome; Analysis 4.3).

We conducted sensitivity analysis by removing two studies (Ciliberto et al., 2005; Sandige et al., 2004) judged to be at high risk of bias for sequence, generation, allocation concealment and blinding. Findings from sensitivity analysis suggest that there was no evidence of effect on weight gain when standard RUTF was compared with energy dense home prepared food (MD: 0.41 g·kg^−1^·day^−1^; 95% CI: −0.16, 0.98; one study; 565 participants; low‐quality outcome; Analysis 4.4).

###### Mortality: Pooled study results

Nine studies (Bahwere et al., 2014, 2016, 2017; Bhandari et al., 2016; Ciliberto et al., 2005; Hsieh et al., 2015; Irena et al., 2015; Jones et al., 2015; Oakley et al., 2010) reported mortality at 8–16 weeks suggesting no evidence of effect on mortality when standard RUTF was compared with non‐milk/peanut butter‐based RUTF (RR: 0.90; 95% CI: 0.72 to 1.12; five studies; 5,743 participants; *I* ^2^ 3%; moderate‐quality outcome; Analysis 4.5); energy dense home prepared food (RR: 1.87; 95% CI: 0.95 to 3.7; two studies; 1,743 participants; *I* ^2^ 0%; moderate‐quality outcome; Analysis 4.5); high oleic RUTF (RR: 5.07; 95% CI: 0.61 to 42.31; one study; 141 participants; low‐quality outcome; Analysis 4.5) and elevated *n*3 PUFA RUTF (RR: 0.33; 95% CI: 0.04 to 2.94; one study; 40 participants; low‐quality outcome; Analysis 4.5).

We conducted sensitivity analysis by removing one study (Ciliberto et al., 2005) judged to be at high risk of bias for sequence generation, allocation concealment and blinding. There was no evidence of effect in mortality when standard RUTF was compared with energy dense home prepared food (RR: 5.09; 95% CI: 0.25, 105.53; one study; 565 participants; Analysis 4.6).

##### Secondary outcomes

Among secondary outcomes, included studies reported height/length gain, MUAC, time to recovery and adverse events. None of the included studies reported any of the other secondary outcomes including stunting, wasting, underweight, infection and costs.

###### Height/length gain: Pooled study results

Five studies (Bahwere et al., 2016; Ciliberto et al., 2005; Hsieh et al., 2015; Oakley et al., 2010; Sandige et al., 2004) reported height gain suggesting no evidence of effect on height gain when standard RUTF was compared with non‐milk/peanut butter‐based RUTF (MD: −0.56 mm/day; 95% CI: −2.29 to 1.17; two studies; 1,037 participants; *I* ^2^ 63%; low‐quality outcome; Analysis 4.7) and high oleic RUTF (MD: −0.09 mm/day; 95% CI: −0.21 to 0.03; one study; 141 participants; moderate‐quality outcome; Analysis 4.7). Standard RUTF probably improves height gain by 0.07 mm/day when compared with energy dense home food (−0.07 mm/day; 95% CI: −0.11 to −0.02; two studies; 1,360 participants; *I* ^2^ 0%; moderate‐quality outcome; Analysis 4.7). However, both the studies in this comparison (Ciliberto et al., 2005; Sandige et al., 2004) were judged to be at high risk of bias for sequence generation, allocation concealment and blinding.

###### MUAC gain: Pooled study results

Six studies (Bahwere et al., 2016; Ciliberto et al., 2005; Hsieh et al., 2015; Oakley et al., 2010; Sandige et al., 2004; Sigh et al., 2018) reported MUAC gain suggesting no evidence of effect on MUAC gain when standard RUTF was compared with non‐milk/peanut butter‐based RUTF (MD: 0.68 mm/day; 95% CI: 0.00 to 1.36; three studies; 2111 participants; I^2^ 97%; low‐quality outcome; Analysis 4.8); energy dense home prepared food (MD: −0.03 mm/day; 95% CI: −0.15 to 0.08; two studies; 1360 participants; I^2^ 81%; low‐quality outcome; Analysis 4.8) and high oleic RUTF (MD: −0.07 mm/day; 95% CI: −0.17 to 0.03; one study; 141 participants; moderate‐quality outcome; Analysis 4.8).

###### Time to recovery: pooled study results

Two studies (Bhandari et al., 2016; Dossou et al., 2003) reported time to recovery suggesting that RUTF might reduce the time to recovery by 3.9 days when compared with F100 (MD: −3.9 days; 95% CI: −6.04 to −1.76; one study; 70 participants; low‐quality outcome; Analysis 4.9) and by 1.2 days when compared with energy dense home prepared food (MD: −1.21 days; 95% CI: −1.92 to −0.5; one study; 565 participants; low‐quality outcome; Analysis 4.9).

###### Adverse events: Pooled study results

There was no difference between standard RUTF and other foods for any of adverse events including cough (RR: 0.97; 95% CI: 0.44 to 2.16; two studies; 1,093 participants; *I* ^2^ 84%; low‐quality outcome; (Analysis 4.10); diarrhoea (RR: 1.01; 95% CI: 0.83 to 1.22; three studies; 1,154 participants; *I* ^2^ 0%; moderate‐quality outcome; Analysis 4.10) and fever (RR: 1.21; 95% CI: 0.61 to 2.39; two studies; 1,154 participants; *I* ^2^ 88%; low‐quality outcome; Analysis 4.10).

###### Hospitalisation: Pooled study results

There was no difference between standard RUTF and other foods for hospitalisation (RR: 0.80; 95% CI: 0.46, 1.39; three studies; 2,479 participants; *I* ^2^ 55%; low‐quality outcome; Analysis 4.11).

#### 5.3.5 Comparison 5: RUSF for MAM compared with standard diet, or FBF or other locally produced foods

Fourteen studies (Ackatia‐Armah et al., 2015; Fabiansen et al., 2017; Karakochuk et al., 2012; LaGrone et al., 2012; Matilsky et al., 2009; Medoua et al., 2015; Nackers et al., 2010; Nikièma et al., 2014; Phuka et al., 2009; Scherbaum et al., 2015; Singh et al., 2010; Stobaugh et al., 2016; Thakwalakwa et al., 2010; Vanelli et al., 2014) compared RUSF for MAM with other foods. Other foods included whey RUSF, energy dense home prepared food, CSB and food supplement.

##### Primary outcome

Among primary outcomes, included studies reported recovery rate, weight gain and mortality. None one of the included studies reported relapse and case fatality rate.

###### Recovery Rate: Pooled study results

Ten studies (Karakochuk et al., 2012; LaGrone et al., 2012; Matilsky et al., 2009; Medoua et al., 2015; Nackers et al., 2010; Nikièma et al., 2014; Phuka et al., 2009; Scherbaum et al., 2015; Stobaugh et al., 2016; Vanelli et al., 2014) reported recovery rate at 6–12 weeks. There was no evidence of effect on recovery rate when standard RUSF was compared with local/home made food (RR: 0.92; 95% CI: 0.64 to 0.33; three studies; 435 participants; *I* ^2^: 82%; low‐quality outcome; Analysis 5.1) while RUSF reduces recovery rate when compared with whey RUSF by 4% (RR: 0.96; 95% CI: 0.92 to 1.00; one study; 2,230 participants; high‐quality outcome; Analysis 5.1). RUSF may improve recovery rate by 7% when compared with CSB (RR: 1.07; 95% CI: 1.02 to 1.13; six studies; 5,744 participants; *I* ^2^: 66%; low‐quality outcome; Analysis 5.1).

We conducted sensitivity analysis by removing one study (Scherbaum et al., 2015) judged to be at high risk of bias for sequence generation, allocation concealment and blinding. There was no evidence of effect on recovery rate when standard RUTF was compared with local/home made food (RR: 1.03; 95% CI: 0.69, 1.54; two studies; 362 participants; *I* ^2^: 78%; low‐quality outcome; Analysis 5.2).

###### Weight gain: Pooled study results

Seven studies (Ackatia‐Armah et al., 2015; LaGrone et al., 2012; Medoua et al., 2015; Nackers et al., 2010; Nikièma et al., 2014; Scherbaum et al., 2015; Stobaugh et al., 2016) reported weight gain at 6–12 weeks. There was no evidence of effect on weight gain when RUSF was compared with local home made food (MD: −0.75 g·kg^−1^·day^−1^; 95% CI: −2.03 to 0.43; one study; 73 participants; low‐quality outcome; Analysis 5.3) and whey RUSF (MD: −0.16 g·kg^−1^·day^−1^; 95% CI: −0.33 to 0.01; one study; 2,230 participants; high‐quality outcome; Analysis 5.3). When compared with CSB, RUSF may improve weight gain (MD: 0.49 g·kg^−1^·day^−1^; 95% CI: 0.10 to 0.87; five studies; 4,354 participants; *I* ^2^: 87%; low‐quality outcome; Analysis 5.3).

One study (Singh et al., 2010) reported overall weight gain in 3 months suggesting that children in RUSF group gained 0.168 kg (95% CI: 0.002 to 0.333; *p *= .046) more weight than the locally made fortified cereal milk supplement group.

###### Mortality: Polled study results

Eight studies (Karakochuk et al., 2012; LaGrone et al., 2012; Matilsky et al., 2009; Medoua et al., 2015; Nackers et al., 2010; Nikièma et al., 2014; Stobaugh et al., 2016; Vanelli et al., 2014) reported mortality at 6–12 weeks. There was no evidence of effect on mortality when RUSF was compared with whey RUSF (RR: 2.11; 95% CI: 0.39 to 11.48; one study; 2,230 participants; high‐quality outcome; Analysis 5.4), CSB (RR: 0.92; 95% CI: 0.51 to 1.67; six studies; 5,744 participants; moderate‐quality outcome; Analysis 5.4) and food supplement (RR: 0.56; 95% CI: 0.05; 6.08; one study; 336 participants; low‐quality outcome; Analysis 5.4).

##### Secondary outcomes

Among secondary outcomes, included studies reported height, MUAC, time to recovery, stunting, wasting, underweight and adverse events. None of the included studies reported costs.

###### Height/length gain: Pooled study results

Nine studies (Ackatia‐Armah et al., 2015; Fabiansen et al., 2017; LaGrone et al., 2012; Nackers et al., 2010; Nikièma et al., 2014; Phuka et al., 2009; Scherbaum et al., 2015; Stobaugh et al., 2016; Thakwalakwa et al., 2010) reported height/length. There was no evidence of effect on height/length gain when RUSF was compared with local/home made food (MD: −0.11; 95% CI: −0.50 to 0.28; three studies; 890 participants; *I* ^2^: 72%; low‐quality outcome; Analysis 5.5), whey RUSF (MD: −0.01; 95% CI: −0.03 to 0.01; one study; 2,230 participants; high‐quality outcome; Analysis 5.5) and CSB (MD: −0.00; 95% CI: −0.02 to 0.01; six studies; 5,794 participants; Analysis 5.5).

We conducted sensitivity analysis by removing one study (Scherbaum et al., 2015) judged to be at high risk of bias for sequence generation, allocation concealment and blinding. There was no evidence of effect on height/length gain when RUSF was compared with local/home made food (MD: 0.05; 95% CI: −0.29, 0.39; two studies; 817 participants; *I* ^2^: 60%; low‐quality outcome; Analysis 5.6).

###### MUAC gain: Pooled study results

Eight studies (Ackatia‐Armah et al., 2015; LaGrone et al., 2012; Medoua et al., 2015; Nackers et al., 2010; Nikièma et al., 2014; Phuka et al., 2009; Stobaugh et al., 2016; Thakwalakwa et al., 2010) reported MUAC gain. RUSF may improve MUAC gain when compared with local/home made food (MD: 0.22; 95% CI: 0.03 to 0.41; two studies; 817 participants; *I* ^2^: 51%; low‐quality outcome; Analysis 5.7), whey RUSF (MD: 0.04; 95% CI: 0.02 to 0.06; one study; 2,230 participants; high‐quality outcome; Analysis 5.7) and CSB (MD: 0.09; 95% CI: 0.04 to 0.13; seven studies; 5,698 participants; *I* ^2^: 53%; low‐quality outcome; Analysis 5.7).

###### Time to recovery: Pooled study results

Five studies (LaGrone et al., 2012; Medoua et al., 2015; Nikièma et al., 2014; Scherbaum et al., 2015; Stobaugh et al., 2016) reported time to recovery. RUSF may reduce time to recovery when compared with local/home made food by 14 days (MD: −14.20 days; 95% CI: −26.08 to −2.32; one study; 55 participants; low‐quality outcome; Analysis 5.8). There was no evidence of effect on time to recovery when RUSF was compared with whey RUSF (MD: −1.10 days; 95% CI: −2.73 to 0.53; one study; 2,230 participants; high‐quality outcome; Analysis 5.8) and CSB (MD: −2.77 days; 95% CI: −8.39 to 2.86; three studies; 3,256 participants; *I* ^2^: 99%; low‐quality outcome; Analysis 5.8).

###### Stunting: Single study results

One study (Phuka et al., 2009) reported moderate stunting suggesting that there was no evidence of effect on moderate stunting when RUSF was compared with local/home made food (MD: 0.85; 95% CI: 0.69 to 1.05; one study; 170 participants; low‐quality outcome; Analysis 5.9).

###### Wasting: Pooled study results

Four studies (LaGrone et al., 2012; Medoua et al., 2015; Nikièma et al., 2014; Phuka et al., 2009) reported wasting. There was no evidence of effect on moderate wasting when RUSF was compared with whey RUSF (RR: 1.22; 95% CI: 0.34 to 4.39; one study; 170 participants; low‐quality outcome; Analysis 5.10) and CSB (RR: 0.93; 0.69 to 1.27; one study; 1,369 participants; low‐quality outcome; Analysis 5.10).

RUSF probably reduces severe wasting by 26% (RR: 0.74; 95% CI: 0.57 to 0.95; three studies; 3256 participants; *I* ^2^: 0%; moderate‐quality outcome; Analysis 5.11) when compared with CSB.

###### Underweight: Single study result

One study (Phuka et al., 2009) reported moderate underweight suggesting no evidence of effect on underweight when RUSF was compared with local/home made food (RR: 1.06; 95% CI: 0.93 to 1.22; one study; 170 participants; low‐quality outcome; Analysis 5.12).

###### Adverse events: Pooled study results

Three studies (LaGrone et al., 2012; Phuka et al., 2009; Thakwalakwa et al., 2010) reported adverse events. There was no difference between RUSF and other foods for fever (RR: 1.44; 95% CI: 0.95 to 2.18; one study; 2083 participants; moderate‐quality outcome; Analysis 5.13), diarrhoea (RR: 1.08; 95% CI: 0.96 to 1.22; three studies; 4,022 participants; *I* ^2^: 0%; moderate‐quality outcome; Analysis 5.13), ALRI (RR: 0.98; 95% CI: 0.75 to 1.29; one study; 2,083 participants; moderate‐quality outcome; Analysis 5.13), other illnesses (RR: 0.78; 95% CI: 0.56 to 1.07; one study; 2,083 participants; moderate‐quality outcome; Analysis 5.13), any adverse events (RR: 1.17; 95% CI: 0.61 to 2.27; one study; 133 participants; low‐quality outcome; Analysis 5.13) and severe adverse events (RR: 2.03; 95% CI: 0.53 to 7.78; one study; 133 participants; low‐quality outcome; Analysis 5.13). RUSF may increase vomiting compared with other foods (RR: 1.39; 95% CI: 1.03 to 1.86; two studies; 1939 participants; low‐quality outcome; Analysis 5.13).

###### Hospitalisation: Pooled study results

Five studies reported hospitalisation suggesting no evidence of effect on hospitalisation between RUSF and other foods (RR: 0.76; 95% CI: 0.34, 1.70; five studies; 4,140 participants; *I* ^2^ 35%; low‐quality outcome; Analysis 5.14).

#### 5.3.6 Comparison 6: Prophylactic use of antibiotics in children with uncomplicated SAM compared with no antibiotics

Three studies (Berkley et al., 2016; Isanaka et al., 2016; Manary et al., 2012) compared prophylactic use of antibiotics in children with uncomplicated SAM with no antibiotics. The antibiotics used for prophylaxis included co‐trimoxazole (Berkley et al., 2016), amoxicillin (Isanaka et al., 2016; Manary et al., 2012) and cefdinir (Manary et al., 2012).

##### Primary outcomes

Among the primary outcomes, included studies reported recovery, weight gain and mortality. None of the included studies reported relapse and case fatality rates.

###### Recovery rate: Pooled study results

**Figure 5:**
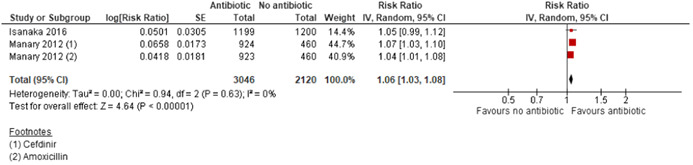
(Analysis 6.1) Forest plot of comparison 6: Prophylactic antibiotics versus no antibiotics, outcome: 6.1 Recovery

Two studies reported recovery rate at 12 weeks suggesting that the recovery in the antibiotic group was 6% better than no antibiotic group (RR: 1.06; 95% CI: 1.03, 1.08; two studies; 5,166 participants; high‐quality outcome; *I* ^2^ = 0%; Analysis 6.1; Figure 5).

###### Weight gain: Pooled study results

Two studies reported weight gain at 12 weeks suggesting that prophylactic antibiotic probably improves weight gain compared with no antibiotic by 0.67 g·kg^−1^·day^−1^ (MD: 0.67; 95% CI: 0.28, 1.08; two studies; 5,052 participants; *I* ^2^ = 49%; moderate‐quality outcome; Analysis 6.2).

###### Mortality: Pooled study results

**Figure 6:**
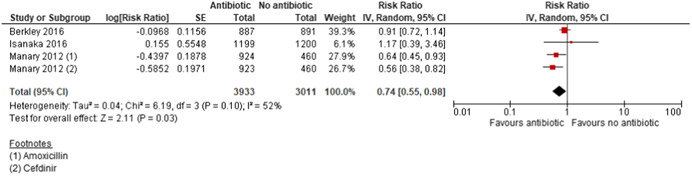
(Analysis 6.3) Forest plot of comparison 6: Prophylactic antibiotics versus no antibiotics, outcome: 6.3 Mortality

Three studies reported mortality at 12 weeks suggesting that prophylactic antibiotic administration probably reduces mortality by 26% compared to the no antibiotics group (RR: 0.74; 95% CI: 0.55, 0.98; three studies; 6944 participants; *I* ^2^ = 52%; moderate quality outcome; Analysis 6.3; Figure 6).

##### Secondary outcomes

Among the secondary outcomes, included studies reported length gain, MUAC gain, time to recovery and adverse events. None of the included studies reported any other secondary outcomes including stunting, wasting, underweight, infection and costs.

###### MUAC gain: Pooled study results

Two studies reported MUAC gain suggesting that the prophylactic antibiotic administration probably improves MUAC by 0.06 mm/day compared with the control group (MD: 0.06 mm/day; 95% CI: 0.04, 0.08; two studies; 5,031 participants; *I* ^2^ = 0%; high‐quality outcome; Analysis 6.4).

###### Length gain: Pooled study results

There was no evidence of difference on length gain when antibiotic was compared with no antibiotic (MD: 0.01; 95% CI: −0.01, 0.04; two studies; 5,052 participants; moderate‐quality outcome; *I* ^2^ = 59%; Analysis 6.5).

###### Time to recovery: Pooled study results

There was no evidence of difference on time to recovery when antibiotic was compared with no antibiotic (MD: −0.25; 95% CI: −1.55, 1.05; one study; 2,442 participants; moderate‐quality outcome; Analysis 6.6).

###### Adverse events: Pooled study results

Three studies reported adverse events suggesting no evidence of difference on diarrhoea (RR: 0.96; 95% CI: 0.80, 1.16; three studies; 6,707 participants; *I* ^2^ = 89%; moderate‐quality outcome; Analysis 6.7) and fever (RR: 0.95; 95% CI: 0.88, 1.04; two studies; 4,926 participants; *I* ^2^ = 0%; high‐quality outcome; Analysis 6.7) between the antibiotic and no antibiotic groups. Prophylactic antibiotic probably decreases ARI symptoms compared with the no antibiotics by 11% (RR: 0.89; 95% CI: 0.82, 0.98; three studies; 6,703 participants; high‐quality outcome; *I* ^2^ = 36%; Analysis 6.7).

###### Hospitalisation: Pooled study results

Three studies reported hospitalisation suggesting that prophylactic antibiotic administration reduces hospitalisation by 11% compared with no antibiotic (RR: 0.89; 95% CI: 0.82, 0.98; three studies; 6,944 participants; *I* ^2^ = 0%; high‐quality outcome; Analysis 6.8).

#### 5.3.7 Comparison 7: Vitamin A supplementation in the management of SAM and MAM with various doses and frequency of administration

Two studies (Donnen et al., 1998; Sattar et al., 2012) compared high‐dose vitamin A supplement with low‐dose vitamin A supplement.

##### Primary outcomes

Among primary outcomes, included studies reported weight gain and mortality. None of the included studies reported other primary outcomes including recovery, relapse and case fatality rates.

###### Weight gain: Single study result

There was no evidence of effect on weight gain at 2 weeks when high dose was compared with low‐dose vitamin A supplementation (MD: 0.05 g·kg^−1^·day^−1^; 95% CI: −0.08, 0.18; one study; 207 participants; moderate‐quality outcome; Analysis 7.1).

###### Mortality: Single study result

There was no evidence of effect on mortality at 15 days when high dose was compared with low‐dose vitamin A supplementation (RR: 7.07; 95% CI: 0.37, 135.13; one study; 207 participants; moderate‐quality outcome; Analysis 7.2).

##### Secondary outcomes

Among the secondary outcomes, included studies reported height change, MUAC change and adverse events. None of the included studies reported other secondary outcome including time to recover, stunting, wasting, underweight, infection and costs.

###### Height gain: Single study result

One study reported height gain suggesting that the high‐dose vitamin A supplementation probably increases height by 0.1 cm compared with the low‐dose group (MD: 0.10; 95% CI: 0.02, 0.18; one study; 207 participants; moderate‐quality outcome; Analysis 7.3).

###### MUAC gain: Single study result

There was no evidence of effect on MUAC gain when high‐dose vitamin A supplementation was compared with low‐dose supplementation (MD: 0.80; 95% CI: −0.46, 2.06; one study; 207 participants; moderate‐quality outcome; Analysis 7.4).

###### Adverse events: Single study result

There was no difference between the high‐dose vitamin A supplementation and low‐dose supplementation groups for adverse events including fever (RR: 1.50; 95% CI: 0.45, 5.05; one study; 122 participants; moderate‐quality outcome; Analysis 7.5) and ALRI (RR: 1.00; 95% CI: 0.07, 13.87; one study; 20 participants; moderate‐quality outcome; Analysis 7.5).

## 6 DISCUSSION

### 6.1 Summary of main results

This review summarises findings from a total of 42 studies (from 48 papers) including 35,017 children. Thirty‐three of the included studies were RCTs; six studies were quasi‐experimental studies and three studies were cost studies. Among primary outcomes, included studies reported recovery rate, weight gain, relapse and mortality; while among the secondary outcomes, studies reported height gain, MUAC gain, time to recover, stunting, wasting, underweight, adverse events and cost‐effectiveness.

Two studies assessed integrated community‐based strategies to screen, identify and manage MAM and SAM compared with no community‐based strategies. Among primary outcomes, integrated community‐based management probably improves recovery rate by 4%, probably decreases weight gain by 0.8 g·kg^−1^·day^−1^ compared with no community‐based management; mortality was similar between the integrated community‐based management group and the no community‐based management group. Among secondary outcomes, the integrated community management probably decreases length gain by 0.1 mm/day, improves MUAC by 0.27 mm/day, reduces diarrhoea by 29% and fever by 15% during the first 2 weeks of feeding compared with the no community‐based management. CMAM was reported to be the most cost‐effective strategy in Malawi.

Four studies assessed facility‐based strategies to screen and manage uncomplicated SAM compared with other standard of care. Findings suggest that among primary outcomes, there was no evidence of difference between WHO protocol and other protocol on recovery. There was no evidence of difference on mortality between the facility‐based management and out‐patient management. Among secondary outcomes only cost and cost‐effectiveness was reported. Findings suggest that the cost for facility‐based care and rehabilitation was significantly higher compared with daycare or ambulatory care services.

Three studies assessed facility‐based management of SAM with RUTF compared with F100. Among primary outcomes, there was no evidence of difference on weight gain and mortality when facility‐based RUTF was compared with F100. Among secondary outcomes, there was no evidence of difference on height gain, MUAC and wasting.

Fourteen studies compared community‐based management of children with uncomplicated SAM with RUTF versus other foods. Among primary outcomes, there was no evidence of difference on recovery rate when standard RUTF was compared with other foods. Standard RUTF probably improves weight gain by 0.5 g·kg^−1^·day^−1^ when compared with non‐milk/peanut butter‐based RUTF and by 5.5 g·kg^−1^·day^−1^ when compared with F100 with no evidence of difference on weight gain when standard RUTF was compared with energy dense home prepared food and high oleic RUTF. There was no evidence of difference on mortality when standard RUTF was compared with other foods. Among secondary outcomes, there was no evidence of difference on height gain when standard RUTF was compared with non‐milk/peanut butter‐based RUTF and high oleic RUTF. Standard RUTF may improve height gain by 0.07 mm/day when compared with energy dense home food. There was no evidence of difference on MUAC gain when standard RUTF was compared with other foods. RUTF might reduce the time to recovery by 3.9 days when compared with F100 and by 1.2 days when compared with energy dense home prepared food. Adverse events and hospitalisation were similar in both the RUTF and other foods groups.

Fourteen studies compared RUSF for MAM with other foods. There was no evidence of difference on recovery when RUSF was compared with local/home made food while RUSF probably reduces recovery rate when compared with whey RUSF by 4%. RUSF probably improves recovery rate by 7% when compared with CSB. There was no evidence of difference on weight gain when RUSF was compared with local home made food and whey RUSF; while RUSF may improve weight gain by 0.49 g·kg^−1^·day^−1^ when compared with CSB. There was no evidence of difference on mortality when RUSF was compared with other foods. Among secondary outcomes, there was no evidence of difference on height gain when RUSF was compared with local/home made food, whey RUSF and CSB. RUSF may improve MUAC gain by 0.22 mm/day when compared with local/home made food, by 0.04 mm/day when compared with whey RUSF and by 0.09 mm/day when compared with CSB. RUSF may reduce time to recovery by 14 days when compared with local/home made food. There was no evidence of difference on time to recovery when RUSF was compared with whey RUSF and CSB. There is no evidence of effect on moderate stunting, moderate wasting and moderate underweight when RUSF was compared with other food. RUSF probably reduces severe wasting by 26% when compared with CSB. Adverse events including fever, diarrhoea, ALRI, other illnesses, any adverse events, severe adverse events and hospitalisations were similar in RUSF and other foods groups while RUSF may increase vomiting compared with other foods.

Three studies compared prophylactic use of antibiotics in children with uncomplicated SAM with no antibiotics. Among the primary outcomes, prophylactic antibiotic therapy improves recovery by 6%, probably improves weight gain by 0.67 g·kg^−1^·day^−1^ and probably reduces mortality by 26% compared to the no antibiotic therapy. Among secondary outcomes, prophylactic antibiotic administration probably improves MUAC gain by 0.06 mm/day and reduces hospitalisation by 11%; while there was no evidence of difference on length gain and time to recovery. There was no difference in diarrhoea and fever between the prophylactic antibiotic and no antibiotic groups while decreased ARI symptoms were reported in the prophylactic antibiotic group compared with no antibiotic group.

Two studies compared high‐dose vitamin A supplementation with low‐dose vitamin A supplementation in children with SAM. Among primary outcomes, there was no evidence of difference on weight gain and mortality when high dose was compared with low‐dose vitamin A supplementation. Among secondary outcomes, high‐dose vitamin A supplementation probably increases height by 0.1 cm compared with the low‐dose group while there was no evidence of difference on MUAC gain. The adverse events including fever and ALRI were similar in both the high‐dose and low‐dose groups.

### 6.2 Overall completeness and applicability of evidence

All the included studies targeted children aged six months to 60 months of age with SAM and MAM; both male and female children were included. We found studies evaluating interventions under all the pre‐specified comparisons. Two studies assessed integrated community‐based strategies to manage MAM and SAM compared with no community‐based strategies; four studies assessed facility‐based strategies to manage uncomplicated SAM compared with other standard of care; three studies assessed facility‐based management of SAM with RUTF compared with F100; fourteen studies compared community‐based management of children with uncomplicated SAM with RUTF versus other foods; fourteen studies compared RUSF for MAM with other foods; three studies compared prophylactic use of antibiotics in children with uncomplicated SAM with no antibiotics; and two studies compared high‐dose vitamin A supplementation with low‐dose vitamin A supplementation in children with SAM. We found very limited number of studies assessing community‐based management (only two studies) and facility‐based management (only five studies) compared with other standards of care. Moreover, there are very few studies assessing the effect of variable dose of vitamin A supplementation (two studies).

Due to the limited number of studies in each comparison, we could not conduct the planned subgroup analysis.

All the studies were conducted in either community, hospital, health centre or nutrition rehabilitation centres in LMICs including Bangladesh, Mali, Malawi, Congo, Kenya, India, Niger, Senegal, Sudan, Burkina Faso, Zambia, Ethiopia, Sierra Leonne, Cameroon, Indonesia and Cambodia.

### 6.3 Quality of the evidence

Majority of the included studies were judged to be at low risk of bias for sequence generation, allocation concealment, incomplete outcome data and selective reporting. Majority of the studies were judged to be at high risk of bias for blinding of participants and personnel and outcome assessment blinding. Majority of the outcomes were rated as either moderate or low‐quality outcomes. Outcomes were downgraded mainly due to study limitations, high heterogeneity, imprecision and small sample size.

Furthermore, heterogeneity in risk of bias could disproportionately affect different outcomes which has studies with small sample sizes. Also for attrition bias, the results could be disproportionately affected, for outcomes where the number of events are small. Although these considerations have been made while grading the outcomes however the findings should be interpreted with caution in this regard.

### 6.4 Potential biases in the review process

We were aware of the possibility of introducing bias at every stage of the reviewing process. A comprehensive search strategy was developed for a list of pre‐identified databases to capture the eligible studies. We tried to minimise bias in a number of ways; two review authors assessed eligibility for inclusion, carried out data extraction and assessed risk of bias. Nevertheless, the process of assessing risk of bias, for example, is not an exact science and includes many personal judgements. While we attempted to be as inclusive as possible in the search strategy, the literature identified was predominantly written in English and published in North American and European journals. Although we did attempt to assess reporting bias, this assessment largely relied on information available in the published trial reports and thus, reporting bias was not usually apparent. We would encourage readers to examine the Characteristics of included studies tables to assist in the interpretation of results.

### 6.5 Agreements and disagreements with other studies or reviews

To the best of our knowledge, this is the only comprehensive systematic review evaluating the interventions to manage acute malnutrition in children under 5 years of age in LMICs. Various systematic reviews have assessed the effectiveness of individual interventions for managing malnutrition in children.

A previous systematic review by Lenters et al. 2013 evaluated the effectiveness of interventions for SAM including the WHO protocol for inpatient management and community‐based management with RUTF, as well as interventions for MAM in children under 5 years in LMIC. This review included 14 studies and suggested that there are still gaps to estimate effectiveness of overall treatment approaches for SAM and MAM. The findings from our review is based on a total of 43 studies (from 49 papers).

Roberfroid et al. (2013) assessed outpatient care of children with nutritional oedema compared with treatment in inpatient care or to treatment of marasmus in outpatient care suggesting that oedematous malnutrition could plausibly be treated effectively in outpatient service. However, the quality of evidence was low and further good‐quality studies in various settings are required before conclusive guidance can be generated. Findings from our review suggests that the outpatient management probably improves recovery compared with the in‐patient group while there was no evidence of impact on mortality. Findings from the included studies on cost‐effectiveness concluded that the cost for in‐patient care and rehabilitation was significantly higher compared with daycare or ambulatory care services.

A recent Cochrane review (Schoonees et al., 2019) assessed the effects of home‐based RUTF used during the rehabilitation phase of SAM in children on recovery, relapse, mortality and rate of weight gain suggesting that compared with alternative dietary approaches, standard RUTF probably improves recovery and may increase rate of weight gain slightly, but the effects on relapse and mortality are unknown. A review by Gera (2010) assessed the efficacy and safety of home‐based management of SAM using RUTF and compared it to F100 and home‐based diet. Findings from this review suggested that the use of RUTF for home‐based management of uncomplicated SAM was safe and efficacious. These findings are similar to the conclusions of our review.

Our findings are in concordance with the review by Williams and Berkley (2018) suggesting the current evidence supports the continued use of broad‐spectrum oral amoxicillin for treating children with uncomplicated SAM as outpatients. Our findings also suggest beneficial effect of prophylactic antibiotic administration on recovery, weight gain, morality and MUAC gain.

## 7 AUTHORS’ CONCLUSIONS

### 7.1 Implications for practice

Findings from this review suggest that there is limited data comparing community‐based management and facility‐based management with other standard of care for SAM/MAM suggesting some benefit of integrated community‐based and outpatient management on improving recovery when compared with standard care and in‐patient management. Existing cost data also suggests that community or out‐patient management of children with uncomplicated SAM is the cost‐effective strategy. Evidence also suggests that facility‐based management of SAM with RUTF is similar to F100 on outcomes of weight gain and mortality.

Existing evidence on RUTF suggests that standard RUTF is comparable with other foods for recovery and mortality for SAM; however standard RUTF may improve weight gain when compared with non‐milk/peanut butter‐based RUTF and F100. Standard RUTF might also reduce recovery time when compared with F100 and energy dense home prepared food.

Existing data on RUSF for the management of MAM suggests that RUSF may improve recovery and weight gain when compared with CSB for MAM.

Data on prophylactic antibiotic administration in children with uncomplicated SAM suggests improved recovery rate, weight gain and mortality when compared with no antibiotic administration.

Limited data suggest that high‐dose vitamin A supplementation is comparable with low‐dose vitamin A supplementation for weight gain and mortality among children with SAM.

### 7.2 Implications for research

Findings from our review provide a number of implications for future research. Existing evidence assessing community and facility‐based strategies to screen, identify and manage SAM and MAM is scarce. We found only two studies assessing community‐based management compared with standard care while five studies assessing hospital‐based management compared with out‐patient care. Future studies are needed to compare the effectiveness of various community and facility‐based strategies including active community‐based surveillance; training of CHWs for community‐based screening; and training of health‐facility staff to diagnose and treat children uncomplicated SAM.

Existing data on the effectiveness of vitamin A supplementation is also limited. We found only two studies assessing the effect of vitamin A supplementation; hence future data are needed to evaluate the role of vitamin A supplementation with various doses and frequency of administration among children with SAM and MAM.

Future studies assessing the effectiveness of interventions to prevent and manage malnutrition among children in LMIC should report pertinent nutrition‐specific outcomes including stunting, wasting, underweight, infections and potential adverse effects. Further studies should assess the relative cost and cost‐effectiveness of various interventions addressing malnutrition in LMICs.

Our review findings are limited in terms of the subgroup analysis since we could not assess if the effect varies by subgroups like age, duration of intervention, various formulations of supplementary foods, various settings, vitamin A dosage, various antibiotics and equity.

Any future studies should be rigorous and powered enough to capture the nutrition outcomes. There are also major research gaps in areas including baseline population‐level micronutrient deficiencies and bioavailability in food preparations in order to enable optimal trials.

## AUTHOR CONTRIBUTIONS

All review authors contributed to the development of the review. J. K. D., R. A. S., M. S. and F. A. K. selected which studies to include, obtained copies of the studies and extracted data from the studies. R. A. S. and J. K. D. entered data into RevMan, carried out the analysis and interpreted the results. J. K. D., R. A. S. and Z. A. B. drafted the final review.

## CONFLICT OF INTERESTS

The authors declare that there are no conflict of interests.

## DIFFERENCES BETWEEN PROTOCOL AND REVIEW

1. We planned to conduct analysis based on six comparisons only in our protocol, however, based on the yield of studies included, we added one separate comparison (Facility management with RUTF vs. F100).
2. Due to the limited number of studies, we could not conduct the planned subgroup analysis.
3. Based on one of the reviewer's comments, we have added one secondary outcome to the review (hospitalisation) which was not included in the outcomes pre‐specified in the protocol.

## PUBLISHED NOTES

**Characteristics of studies**

**Characteristics of included studies**

Ackatia‐Armah et al. (2015)

**Table:** 

Methods	Design: Cluster RCT
	Unit: Cluster
Participants	Location/Setting: Study was carried out in twelve community health centres in rural setting in Diola Health District, Bamako, Mali
	Sample size: 1,264 children aged 6–35 months
	Dropouts/Withdrawals: 20 lost to follow‐up
	Sex: Both male and female children included
	Mean age: 14.9 (±6.2) months
	Inclusion criteria: Children were identified as having MAM by using 2 sets of criteria. The first set of criteria was based on the 2006 WHO Growth Standards while the second set of entry criteria was based on the national norms that were being used in Mali at the time of the study. All children meeting one of these sets of criteria and without edema were referred to the community health centres for possible study enrolment
	Exclusion criteria: Children were excluded if they had severe anaemia; severe acute malnutrition or MUAC; other acute illnesses requiring inpatient treatment; congenital abnormalities or underlying chronic diseases, including known HIV infection that might interfere with nutritional recovery; or a history of allergy to peanuts or previous serious allergic reactions to any substance and requiring emergency medical care
Interventions	Intervention: Each supplement was supplied in an amount to provide 500 kcal/day, which was to be consumed in addition to the usual home diet
	Group 1: Ready‐to‐use supplementary food (RUSF) (*n *= 344)
	Supplements contained RUSF; supplied by Nutriset: a lipid‐based RUSF (Supplementary Plumpy) containing peanut paste, sugar, vegetable oil, whey and soy protein isolates, maltodextrin and cocoa flavouring, and a vitamin‐mineral complex. The RUSF was supplied in 92‐g sachets for daily use; 7 sachets/week were provided
	Group 2: Corn–soy blend (CSB++) (*n *= 349)
	1 kg bag of supplements/week on a weekly basis was given for 12 weeks. Supplements contained corn‐soy blend “plus plus” (CSB++; supplied by the World Food Program), a specially formulated refined cereal‐legume‐milk blend for children with MAM, which contains dehulled soybean flour, maize flour, dried skimmed milk, soy, sugar, soya oil, and a micronutrient premix
	Group 3: Misoloa (MI) (*n *= 307)
	1 kg bag of supplements/week on a weekly basis was given for 12 weeks. Supplements contained Misola (supplied by Misola, Mali), a less‐refined micronutrient‐fortified cereal‐legume blend, containing 60% millet or maize flour, 20% soy flour, 10% peanut flour, micronutrient premix, and amylase powder
	Group 4: Locally milled flours + micronutrient powder (LMF) (*n *= 284)
	1 kg bag of supplements/week on a weekly basis was given for 12 weeks. Supplements contained a less‐refined cereal‐legume milled flour mix (LMF), a mixture of home‐available foods, including millet (605 g/kg food mixture), beans (273 g/kg), sugar (32 g/kg) and oil (90 g). In addition, multiple micronutrient powder sachets (Mixme, supplied by DSM) were given to the LMF group in accordance with the Malian national treatment protocol
	Intervention duration was 12 weeks
Outcomes	Primary outcomes: Adherence to treatment, MUAC, body weight, length, WLZ, LAZ, anaemia, iron deficiency, iron deficiency anaemia, haemoglobin, plasma ferritin, retinol‐binding protein, transferrin receptor, body iron stores, plasma zinc
	Timing of outcome assessment: 1, 2, 3, 4, 6, 8, 10 and 12 weeks
Notes	Study start date: May 2010
	Study end date: May 2011
	Funding source: Supported by UNICEF Mali and UNICEF West and Central Africa (WACARO), the World Food Program (WFP), the Goldman Fund, and Helen Keller International (HKI). The CSB++ was donated by the World Food Program, Rome, Italy; the ready‐to‐use supplementary food was donated by Nutriset, Malaunay, France
	Conflicts of interest: None declared

Risk of bias table

**Table:** 

Bias	Authors’ judgement	Support for judgement
Random sequence generation (selection bias)	Unclear risk	Quote: “CSComs were then randomly assigned to one of 4 dietary interventions within each stratum at the beginning of the study, and they were randomly reassigned, within stratum, to a different dietary group after the first 3 rounds of screening”
		Comment: Insufficient information to permit judgement
Allocation concealment (selection bias)	Unclear risk	Quote: “CSComs were then randomly assigned to one of 4 dietary interventions within each stratum at the beginning of the study, and they were randomly reassigned, within stratum, to a different dietary group after the first 3 rounds of screening”
		Comment: Insufficient information to permit judgement
Blinding of participants and personnel (performance bias)	High risk	Comment: Probably not done
Blinding of outcome assessment (detection bias)	High risk	Comment: Probably not done
Incomplete outcome data (attrition bias)	Low risk	Comment:
		Group 1 (RUSF): 9/344
		Group 2 (CSB++): 7/349
		Group 3 (MI): 1/307
		Group 4 (LMF): 3/284
Selective reporting (reporting bias)	Low risk	Comment: This trial was registered at clinicaltrials.gov as NCT01015950
Other bias	Low risk	Comment: No other biases identified

Ashworth et al. (1994)

**Table:** 

Methods	Design: RCT
	Unit: Individual
Participants	Location/Setting: Study was carried out in The Children's Nutrition Unit in central Dhaka, Bangladesh. Most children (90%) come from urban slums, either brought by their families (60%) or referred from other hospitals
	Sample size: 573 children aged 12‐60 months
	Dropouts/Withdrawals: 136 lost to follow‐up
	Sex: Both male and female participants
	Mean age: 12–60 months
	Inclusion criteria: Children with severe malnutrition anticipated not to require daily treatment after 7 days
	Exclusion criteria: Children aged less than 12 months or more than 60 months, children with tuberculosis, congenital or metabolic transformation or home more than 10 km from unit. Children with conditions which might require more than 7 days’ medical supervision
Interventions	Intervention:
	Group 1: Inpatient (*n *= 200)
	Children were admitted with their mothers and were resident until they reached 80% wt/ht. 80–100 ml·kg^−1^·day^−1^ of modified milk (75 kcal and 1.5 g protein per 100 ml) was given every 2 hr and 4 rice‐based, salt‐free meals in week 1. Week 2 onwards high energy milk (100 kcal and 3 g protein per 100 ml) and 2 rice‐based, salt‐free meals and 2 snacks were given.This was continued until 80% weight per height was achieved
	Group 2: Day care (*n *= 200)
	Children came with their mothers from 0800 to 1700 hr every day except Friday, until 80% wt/ht was reached. If mothers wished, they were permitted to bring with them another young sibling. Milk feeds were given every 2 hr and 3 rice‐based, salt‐free meals between 0800 and 1700 and 2 milk feeds and 1 meal at home in week 1. Week 2 onwards 3 milk feeds and 3 rice‐based, salt‐free meals and 2 snacks were given between 0800 and 1700 and 1 milk feed and 2 snacks were given at home
	Group 3: Care at home (*n *= 173)
	Children were treated in the day‐care facility for 7 days (or up to 9 days if poor appetite or poor clinical condition persisted). Thereafter they were visited at home weekly for one month, then twice monthly until they reached 80% wt/ht. Weekly visits continued if children were not oedema‐free at one month. Only multivitamins and ferrous sulphate were provided for home use, in contrast to the other groups which received potassium chloride, magnesium sulphate, riboflavin, and folic acid. No food supplements were distributed. Milk feeds were given every 2 hr and 3 rice‐based, salt‐free meals between 0800 and 1700 and 2 milk feeds and 1 meal at home in week 1. Week 2 onwards 3 milk feeds, 2 rice‐based, salt‐free meals and 2 snacks were given. This was given weekly for the first month and every 2 weeks afterwards
	All meals contained rice pudding, rice with dhal, rice with pumpkin, potato, fish or meat
	Duration of intervention: Till recovery
Outcomes	Primary outcomes: Cost‐effectiveness
	Secondary outcomes: Mortality, rate of edema loss, weight gain and days taken to achieve 80% oedema‐free weight/height
	Timing of outcome assessment: Weight was recorded daily and height weekly for inpatient and daycare assessment. For interventions administered at home, assessment was done weekly for a month and then fortnightly until they reached 80% wt/ht
Notes	Study start date: December 1990
	Study end date: November 1991
	Funding source: Funding was provided by Save the Children Fund, UK
	Conflicts of interest: One of the authors, S. R. A. H. is supported by the UK Overseas Development Administration

Risk of bias table

**Table:** 

Bias	Authors’ judgement	Support for judgement
Random sequence generation (selection bias)	Unclear risk	Quote: “Children were allocated to three groups by daily rotation. The initial sequence was randomly determined”
		Comment: Insufficient information to permit judgement
Allocation concealment (selection bias)	Low risk	Quote: “Allocation to treatment groups was made by daily rotation such that recruitment to each group occurred every third day. Neither mothers nor admission officers were aware of which treatment was available on a particular day”
		Comment: Adequately done
Blinding of participants and personnel (performance bias)	High risk	Comment: Not done
Blinding of outcome assessment (detection bias)	High risk	Comment: Not done
Incomplete outcome data (attrition bias)	High risk	Comment:
		Group 1 (Inpatient): 27/200
		Group 2 (day‐care): 66/200
		Group 3 (at‐home): 43/173
		Comments: Reasons provided for loss to follow‐up
Selective reporting (reporting bias)	Unclear risk	Comment: Trial registration number not provided. Outcomes described in methodology section were reported in results section
Other bias	Low risk	Comment: No other biases identified

Bahwere et al. (2014)

**Table:** 

Methods	Design: RCT
	Unit: Individual
Participants	Location/Setting: Study was carried out in Lilong Health District, Central Malawi
	Sample size: 600 children aged 6–59 months of age
	Dropouts/Withdrawals: 5 lost to follow‐up
	Sex: Both male and female children were included
	Mean age: 26.3 (±13) months
	Inclusion Criteria: Children with uncomplicated SAM
	Exclusion Criteria: Children with SAM who presented with complications were referred to one of the three hospitals serving as inpatient stabilisation units and were eligible for inclusion in the study only when referred back to the OTP. Children previously discharged from the study with a recovered outcome that later relapsed and who then presented again at one of the participating OTPs with a new episode of SAM were not eligible for enrolment in the study for a second time. Children with any neurological or gastrointestinal chronic disability were also not eligible
Interventions	Intervention:
	Group 1: (*n *= 308)
	Whey‐Protein Concentrate (WPC)—34% used by replacing dried skimmed milk (DSM) was given weekly. 1 week ration = 175 kcal/kg
	Group 2: (*n *= 292)
	P‐RUTF Peanut‐based RUTF was given weekly
Outcomes	Primary outcomes: Average weight gain and recovery rate
	Secondary outcomes: Length of stay (LOS)
	Timing of outcome assessment: Children were followed‐up once every week till discharge
Notes	Study start date: March 2010
	Study end date: March 2011
	Funding source: USDEC through Clinton Foundation provided funding for the study
	Conflicts of interest: One of the authors (V. O.) was an employee of Valid Nutrition. S. C. is the unpaid director of Valid Nutrition.Valid International is the sister company of Valid Nutrition that promotes the development and promotion of RUTF

Risk of bias table

**Table:** 

Bias	Authors’ judgement	Support for judgement
Random sequence generation (selection bias)	Low risk	Quote: “A computer generated sequentially numbered randomisation list (with variable block sizes) that contained both allocations and codes for 700 children was pre‐prepared by the trial statistician based outside Malawi and sent to the national study coordinator who then prepared 700 opaque, sealed and consecutively numbered randomisation envelopes”
		Comment: Adequately done
Allocation concealment (selection bias)	Low risk	Quote: “A computer generated sequentially numbered randomisation list (with variable block sizes) that contained both allocations and codes for 700 children was pre‐prepared by the trial statistician based outside Malawi and sent to the national study coordinator who then prepared 700 opaque, sealed and consecutively numbered randomisation envelopes”
		Comment: Adequately done
Blinding of participants and personnel (performance bias)	Low risk	Quote: “…investigators and non‐participating staff and caregivers were blinded to the letter codes and the identity of RUTF”
		Comment: Adequately done
Blinding of outcome assessment (detection bias)	Low risk	Quote: “The investigators directly involved in supervision, child recruitment, and management and outcome assessment were blinded to the identity of the letter codes”
		Comment: Adequately done
Incomplete outcome data (attrition bias)	Low risk	Comment: 5/600 lost to follow‐up
Selective reporting (reporting bias)	Unclear risk	Comment: Trial registration not specified. Outcomes described in methodology section reported in results section
Other bias	Low risk	Comment: No other biases identified

Bahwere et al. (2016)

**Table:** 

Methods	Design: RCT
	Unit: Individual
Participants	Location/Setting: Study was carried out in a rural setting in Kabare Adminstrative Zone of South Kivu Province, Democratic Republic of Congo
	Sample size: 886 children; 6–23 months (*n *= 414), 24–59 months (*n *= 472)
	Dropouts/Withdrawals: 11 lost to follow‐up
	Sex: Both male and female children were included
	Mean age: 29.4 months
	Inclusion criteria: Children aged 6–59 months diagnosed with SAM. Those with bilateral pitting edema assessed as +++ or with any medical complications, and with any medical or nutritional complication during follow‐up were referred to the participating inpatient facility for appropriate treatment of the complication, after which they were readmitted into the day care program and remained in their original study group
	Exclusion criteria: Children without the presence of edema, with congenital or acquired disorders, any history of food allergies, a history of being treated for SAM within the previous 3 months were excluded
Interventions	Intervention:
	Group 1: (*n *= 445)
	Soya–Maize–Sorghum RUTF
	Group 2: (*n *= 441)
	Standard peanut paste‐based RUTF
Outcomes	Primary outcomes: Recovery rate, mean daily weight gain and mean length of stay
	Secondary outcomes: Hemoglobin change and differences in fat mass, body fat, percentage and fat mass index, fat free mass and fat‐free mass index), bio‐electrical impedance analysis, illness marker and plasma concentrations of 8 key amino acids
	Timing of outcome assessment: Children were followed till discharge
Notes	Study start date: March 2013
	Study end date: Februray 2014
	Funding source: Supported by the PRANA Foundation and Irish Aid
	Conflicts of interest: None declared

Risk of bias table

**Table:** 

Bias	Authors’ judgement	Support for judgement
Random sequence generation (selection bias)	Low risk	Quote: “A computer‐generated sequentially numbered randomisation list (with variable block sizes) that contained both allocations and codes for 900 children was pre‐prepared by the trial statistician, who was based outside the DRC”
		Comment: Adequately done
Allocation concealment (selection bias)	Low risk	Quote: “After confirming eligibility for inclusion, children were randomly assigned by a closed‐envelope method to receive either SMSRUTF or P‐RUTF”
		Comment: Adequately done
Blinding of participants and personnel (performance bias)	High risk	Quote: “Differences in the colour and taste between the SMS‐ and P‐RUTF precluded blinding the study”
		Comment: Not done
Blinding of outcome assessment (detection bias)	High risk	Quote: “Differences in the colour and taste between the SMS‐ and P‐RUTF precluded blinding the study”
		Comment: Not done
Incomplete outcome data (attrition bias)	Low risk	Comment:
		Group 1: 6/445
		Group 2: 5/441
Selective reporting (reporting bias)	Low risk	Comment: Trial registered as PACTR201303000475166 and pre‐specified outcomes were reported
Other bias	Low risk	Comment: No other biases identified

Bahwere et al. (2017)

**Table:** 

Methods	Design: RCT
	Unit: Individual
Participants	Location/Setting: Study was carried out in 3 districts: Lilongwe, Dedza, Mchinji of Malawi; 21 clusters in each district
	Sample size: 1,347 children; *n *= 823 (6–23 months), *n *= 524 (24–59 months)
	Dropouts/Withdrawals: 48 lost to follow‐up
	Sex: Both male and female children were included
	Mean age: 24 months
	Inclusion criteria: Children aged 6–59 months, with SAM [defined as a mid‐upper arm circumference (MUAC), 115 mm or bilateral pitting edema of any degree]. Children with any medical or nutritional complications during follow‐up were referred first to inpatient facility for appropriate treatment, after which they were readmitted into the daycare program and remained in their original study group
	Exclusion criteria: Children excluded from the study if there is no sign of edema. Children with congenital or acquired disorders affecting growth, any history of any food allergy or intolerance, or a history of treatment of SAM in the previous 3 months and children from visiting families were also excluded
Interventions	Intervention:
	Group 1: (*n *= 454)
	PM‐RUTF: Peanutbutter, milk powder, sugar, veg oil, vit/min was given daily (0800–1600) till discharge or gained weight
	Group 2: (*n *= 458)
	FSMS: Amino‐acid enriched milk‐free, soya, maize, sorghum was given daily (0800‐1600 hr) till discharged or gained weight
	Group 3: (*n *= 435)
	MSMS: Amino acid‐enriched low cow milk (9.3%), soya, maize, sorghum given daily (0800–1600 hr) till discharged or desired weight
	NO RUTF at home
Outcomes	Primary outcomes: Recovery rate, mean length of stay and mean daily weight gain.
	Secondary outcomes: Hemoglobin, body iron stores, RUTF intake and morbidity
	Timing of outcome assessment: Children were followed up till discharge
Notes	Study start date: March 2013
	Study end date: Feb 2014
	Funding source: Supported by Ajinomoto Co. Inc., the Japan International Cooperation Agency and the Global Innovation Fund
	Conflicts of interest: None declared

Risk of bias table

**Table:** 

Bias	Authors’ judgement	Support for judgement
Random sequence generation (selection bias)	Low risk	Quote: “The trial statistician, who was based outside Malawi, prepared a computer‐generated sequentially numbered randomisation list (with variable block sizes) that contained the allocations and codes for each site”
		Comment: Adequately done
Allocation concealment (selection bias)	Low risk	Quote: “…we used a closed envelope method to randomly assign children to receive the closed‐envelope…”
		Comment: Adequately done
Blinding of participants and personnel (performance bias)	High risk	Quote: “This non‐blinded, 3‐arm, parallel‐group simple randomised…”
		Comment: Not done
Blinding of outcome assessment (detection bias)	High risk	Quote: “This non‐blinded, 3‐arm, parallel‐group simple randomised…”
		Comment: Not done
Incomplete outcome data (attrition bias)	Low risk	Comment:
		Group 1: 8/454
		Group 2: 25/458
		Group 3: 15/435
Selective reporting (reporting bias)	Low risk	Comment: This trial was registered at www.pactr.org as PACTR201505001101224 and all pre‐specified outcomes were reported
Other bias	Low risk	Comment: No other biases identified

Berkley et al. (2016)

**Table:** 

Methods	Design: RCT
	Unit: Individual
Participants	Location/Setting: Study was carried out in four hospitals in Kenya (two rural hospitals in Kilifi and Malindi, and two urban hospitals in Mombasa and Nairobi
	Sample Size: 1781 children aged 60 days to 59 months
	Dropouts/Withdrawals: 352 lost to follow‐up
	Sex: Both male and female children were included
	Mean age: 11 months
	Inclusion criteria: Children were eligible for inclusion if they were aged between 60 days and 59 months old, and had a diagnosis of SAM on the basis of mid‐upper‐arm circumference or presence of kwashiorkor; had a negative HIV rapid‐antibody test; and had completed the stabilisation phase of treatment as defined in WHO guidelines
	Exclusion criteria: Exclusion criteria were a known allergy to co‐trimoxazole; if co‐trimoxazole was specifically contraindicated; serious comorbidity likely to be associated with mortality unrelated to infection such as severe heart disease or malignancy; and residence outside the attending hospital's catchment area
Interventions	Intervention:
	Group 1: (*n *= 887)
	Daily treatment with water‐dispersible co‐trimoxazole tablets for 6 months
	For children under 6 months: 120 mg/dl
	For children over 6 months: 240 mg/dl
	Group 2: (*n *= 891)
	Placebo given daily for 6 months
	Children were followed for 12 months; they were initially monitored monthly for 6 months and then every 2 months
Outcomes	Primary outcomes: Mortality
	Secondary outcomes: Frequency of non‐fatal illness episodes resulting in readmission to hospital or outpatient attendance; the clinical syndromes associated with death or illness; pathogens detected from blood culture, urine culture, and malaria testing; suspected toxic effects during the period that investigational products were received; and changes in MUAC, weight‐for‐height, weight‐for‐length, weight‐for‐age, height‐for‐age, length‐for‐age, head circumference‐for‐age, and haematological indices
	Timing of outcome assessment: Outcomes assessed at 6 months and at 12 months
Notes	Study start date: November 2009
	Study end date: March 2013
	Funding source: Wellcome Trust, UK
	Conflicts of interest: None declared

Risk of bias table

**Table:** 

Bias	Authors’ judgement	Support for judgement
Random sequence generation (selection bias)	Low risk	Quote: “The assignment was undertaken by the trial statistician (GF) with computer‐generated randomisation of study numbers in permuted blocks of 20, stratified according to clinical centre and age older or younger than 6 months”
		Comments: Adequately done
Allocation concealment (selection bias)	Low risk	Quote: “Allocation was concealed in opaque sealed envelopes that were externally labelled with study numbers and used sequentially at each site. Treatment packs were likewise pre‐labelled with the study numbers according to the randomisation schedule”
		Comments: Adequately done
Blinding of participants and personnel (performance bias)	Low risk	Quote: “All patients, their families, and trial staff were masked to the treatment assignment. Active and placebo blister‐packed water‐dispersible tablets were identical in appearance and dissolution”
		Comments: Adequately done
Blinding of outcome assessment (detection bias)	Low risk	Quote: “All patients, their families, and trial staff were masked to the treatment assignment. Active and placebo blister‐packed water‐dispersible tablets were identical in appearance and dissolution”
		Comments: Adequately done
Incomplete outcome data (attrition bias)	Low risk	Comment:
		Group 1: 170/888
		Group 2: 182/893
Selective reporting (reporting bias)	Low risk	Comment: This trial was registered at ClinicalTrials.gov, number NCT0093449 and all pre‐specified outcomes were reported
Other bias	Low risk	Comment: No other biases identified

Bhandari et al. (2016)

**Table:** 

Methods	Design: RCT
	Unit: Individual
Participants	Location/Setting: Study was carried out in a mixed setting of Rajasthan, Delhi and Tamil Nadu areas of India, with low‐income households with a mix of rural and urban areas
	Sample size: 906 children aged 6–59 months
	Dropouts/Withdrawals: 68 lost to follow‐up
	Sex: Both male and female children were included
	Mean age: 25.3 months
	Inclusion criteria: Children aged 6–59 months with uncomplicated SAM (Children with WHZ < −3 *SD* or oedema of feet, or both were identified as SAM). Children with uncomplicated SAM, whose families were likely to remain in the study area over the next 4 months, and whose parents gave written informed consent were enrolled
	Exclusion criteria: Children with severe illness and a sibling previously enrolled in the study were excluded
Interventions	Intervention:
	Group 1: RUTF‐C (*n *= 298)
	Commercial—peanut paste, sugar, milk solids, veg oil, min/vit mix given weekly for 16 weeks
	Group 2: RUTF‐L (*n *= 307)
	Local—peanut paste, sugar, milk solids, veg oil, min/vit mix given weekly for 16 weeks
	Control: A‐HPF (*n *= 301)
	Cereals/pulses/sugar/oil/milk/eggs/MIN‐Vit mix given weekly for 16 weeks
	Intervention duration: 16 weeks
Outcomes	Primary outcomes: Recovery (defined as WHZ ≥ −2 *SD* of the WHO standards and absence of oedema of feet) by 16 weeks after enrolment
	Secondary outcomes: Weight gain, time to recovery, prevalence of diarrhoea, acute lower respiratory tract infection (ALRI) and fever, mortality and hospitalisations during the treatment phase (until recovery or 16 weeks after enrolment, whichever was earlier). Also, the proportion of children with WHZ ≥ −2 *SD* at the end of the sustenance phase (16 weeks after completion of the treatment phase)
	Timing of outcome assessment: Outcomes were assessed after the treatment phase (16 weeks of treatment) and after the sustenance phase (16 weeks after the end of the treatment phase)
Notes	Study start date: October 2012
	Study end date: April 2015
	Funding source: The trial was funded by the Bill & Melinda Gates Foundation (grant number OPP1033634)
	Conflicts of interest: Two authors R. B. and S. Y. are staff members of the WHO while others declare no conflict of interest

Risk of bias table

**Table:** 

Bias	Authors’ judgement	Support for judgement
Random sequence generation (selection bias)	Low risk	Quote: “A WHO statistician, not otherwise involved with the study, prepared randomisation lists”
		Comment: Adequately done
Allocation concealment (selection bias)	Low risk	Quote: “Allocation into study groups was concealed using Serially Numbered Opaque Sealed Envelopes (SNOSE) prepared by the WHO. The SNOSE next in sequence was opened only after completing an enrolment”
		Comment: Adequately done
Blinding of participants and personnel (performance bias)	High risk	Quote: “Blinding could have further reduced the risk of bias but this was not feasible as the three interventions had visibly different characteristics—one was provided in packets, the other in jars and the third comprised of food ingredients”
		Comment: Not done
Blinding of outcome assessment (detection bias)	High risk	Quote: “Blinding could have further reduced the risk of bias but this was not feasible as the three interventions had visibly different characteristics—one was provided in packets, the other in jars and the third comprised of food ingredients”
		Comment: Not done
Incomplete outcome data (attrition bias)	Low risk	Comment:
		Group 1: ROTF‐C: 25/298
		Group 2: RUTF‐L: 23/307
		Group 3: A‐HPF: 2/301
Selective reporting (reporting bias)	Low risk	Comment: The trial is registered as NCT01705769 and the outcomes pre‐specified have been reported
Other bias	Low risk	Comment: No other biases identified

Chapko et al. (1994)

**Table:** 

Methods	Design: RCT
	Unit: individual
Participants	Location/Setting: Study was carried out in Niger's National Hospital, Niamey, Niger
	Sample size: 100 malnourished children
	Dropouts/Withdrawals: 14 children lost to follow‐up
	Sex: Both male and female children were included
	Mean age: 5–28 months children (mean age not provided)
	Inclusion criteria: Children about to be discharged from the Pediatrics Service at the National Hospital, weight‐for‐height below −2 *SD* or a diagnosis of kwashiorkor, residing within Niamey, and mother's willingness to have her child randomised to receive either hospital or ambulatory rehabilitation
	Exclusion criteria: Not specified
Interventions	Intervention:
	Group 1 (*n *= 53): Hospital‐based rehabilitation. Food was prepared by the hospital kitchen and 3 meals per day were given. All supplies were provided by hospital
	Group 2 (*n *= 47): Ambulatory‐based rehabilitation. Food was prepared at the centre by mothers;supplies were provided partially by the mothers and the centre
	Duration of intervention: Till discharge
Outcomes	Primary outcomes: Cost of care
	Secondary outcomes: Mortality and anthropometric measures
	Timing of Outcome Assessment: Follow‐up anthropometric assessments of the child and brief interviews of the mother were obtained at 15, 30, 60, 90 and 180 days post‐discharge from the Pediatric Service. If the child was living in the hospital rehabilitation unit, the follow‐up assessments were obtained in the hospital
Notes	Study start date: March 1990
	Study end date: April 1991
	Funding source: This study was supported in part by a Fulbright Fellowship to Dr. Chapko
	Conflicts of interest: Not specified

Risk of bias table

**Table:** 

Bias	Authors’ judgement	Support for judgement
Random sequence generation (selection bias)	Unclear risk	Quote: “Between March 1990 and April 1991, 100 malnourished children at Niger's National Hospital were randomised to receive either hospital or ambulatory nutritional rehabilitation and then followed for a period of 6 months”
		Comment: Not clearly specified
Allocation concealment (selection bias)	Unclear risk	Quote: “Between March 1990 and April 1991, 100 malnourished children at Niger's National Hospital were randomised to receive either hospital or ambulatory nutritional rehabilitation and then followed for a period of 6 months”
		Comment: Not clearly specified
Blinding of participants and personnel (performance bias)	High risk	Comment: Probably not done
Blinding of outcome assessment (detection bias)	High risk	Comment: Probably not done
Incomplete outcome data (attrition bias)	Low risk	Comment:
		Group 1: 7/53
		Group 2: 7/47
Selective reporting (reporting bias)	Unclear risk	Comment: Trial registration number not specified. Outcomes specified in the methodology were reported in the results section
Other bias	Low risk	Comment: No other biases identified

Ciliberto et al. (2005)

**Table:** 

Methods	Design: Quasi‐experimental study (allocation is not truly random)
	Unit: Not applicable
Participants	Location/Setting: Study was carried out in a rural setting in South Malawi
	Sample size: 1,178 children 10–60 months of age
	Dropouts/Withdrawals: 113 lost to follow‐up. (98 in RUTF; 13 in STD therapy)
	Sex: Both male and female children were included
	Mean age: 23.5 months
	Inclusion criteria: Children with wasting, mild edema, or both and a good appetite were eligible for participation in the study
	Exclusion criteria: Children aged less than 10 months were excluded. Also, children with severe edema, evidence of systemic infection, or anorexia were excluded from the study
Interventions	Intervention:
	Group 1: (*n *= 992)
	Home‐based therapy with RUTF (HBT‐RUTF): Energy dense lipid paste containing peanut butter,milk, sugar,oil, vit, mineral (CMV; Nutriset) given daily for 8 weeks
	Group 2: (*n *= 186)
	F100‐Standard Inpatient Therapy: F100 + maize/soy blended flour + supplemented with vit. + min given daily (7 times/day) for 8 weeks
	Duration of intervention: 8 weeks
Outcomes	Primary outcomes: Case fatality rate, successful recovery and relapse or death
	Secondary outcomes: Rates of growth in body weight, MUAC, and length. Number of days of fever, cough and diarrhoea during the first 2 weeks of treatment were also recorded
	Timing of outcome assessment: At 8 weeks and then 6 months after recovery
Notes	Study start date: December 2002
	Study end date: June 2003
	Funding source: Supported by the Doris Duke Clinical Scholars Program, the St Louis Children's Hospital Foundation, the World Food Programme, and Valid International. This publication was made possible through support provided to the Food and Nutrition Technical Assistance (FANTA) Project by the Office of Foreign Disaster Assistance of the Bureau for Democracy, Conflict and Humanitarian Assistance, and the Office of Health, Infectious Diseases and Nutrition of the Bureau for Global Health at the U.S. Agency for International Development, under terms of Cooperative Agreement no. HRN‐A‐00‐98‐00046‐00 awarded to the Academy for Educational Development (AED)
	Conflicts of interest: None declared

Risk of bias table

**Table:** 

Bias	Authors’ judgement	Support for judgement
Random sequence generation (selection bias)	High risk	Quote: “The major limitation of the study design was that children were not randomly assigned to either standard therapy or home‐based therapy with RUTF because of the operational nature of this investigation”
		Comment: Not done
Allocation concealment (selection bias)	High risk	Quote: “The major limitation of the study design was that children were not randomly assigned to either standard therapy or home‐based therapy with RUTF because of the operational nature of this investigation”
		Comment: Not done
Blinding of participants and personnel (performance bias)	High risk	Comment: Not done
Blinding of outcome assessment (detection bias)	High risk	Comment: Not done
Incomplete outcome data (attrition bias)	Low risk	Comment:
		Group 1: 98/992
		Group 2: 15/186
Selective reporting (reporting bias)	Unclear risk	Comment: Trial registration not found. Outcomes pre‐specified in the methods section were reported in the results section
Other bias	Low risk	Comment: No other biases identified

Dossou et al. (2003)

**Table:** 

Methods	Design: RCT
	Unit: Individual
Participants	Location/Setting: Study was carried out in an urban setting in Rebuss, Dakar, Senegal
	Sample size: 70 children aged 6–36 months
	Dropouts/Withdrawals: 10 children lost to follow up
	Sex: Both male and female children were included
	Mean age: 16.8 months
	Inclusion criteria: Severely malnourished children defined on admission, or after edema resolved, by a weight‐for‐height (WHZ) *z‐*score <−2 were eligible for inclusion in the study
	Exclusion criteria: Not specified
Interventions	Intervention:
	Group 1: RUTF (*n *= 35)
	Peanut butter‐based (Nutriset) given 3 times/day till discharge
	Control (sample size): F100 (*n *= 35)
	Skim milk‐based (NUTRISET) given 3 times/day till discharge
	Duration of intervention: Till discharge
Outcomes	Primary outcomes: Weight gain
	Secondary outcomes: Food intake (daily energy and macronutrient intakes)
	Timing of outcome assessment: At the time of discharge
Notes	Study start date: March 2001
	Study end date: September 2001
	Funding source: Supported by the University Cheikh Anta Diop, Dakar, and a grant from the Nestlé Foundation. The milk‐based diet (F100) and the ready‐to‐use food (RTUF) were provided by Nutriset (76770 Malaunay, France)
	Conflicts of interest: None declared

Risk of bias table

**Table:** 

Bias	Authors’ judgement	Support for judgement
Random sequence generation (selection bias)	Low risk	Quote: “Group allocation was made from a computer‐generated random number list”
		Comment: Adequately done
Allocation concealment (selection bias)	Unclear risk	Quote: “Group allocation was made from a computer‐generated random number list”
		Comment: Insufficient information
Blinding of participants and personnel (performance bias)	High risk	Quote: “F100 and RTUF looked different; therefore, the trial was not blind”
		Comment: Not done
Blinding of outcome assessment (detection bias)	High risk	Quote: “F100 and RTUF looked different; therefore, the trial was not blind”
		Comment: Not done
Incomplete outcome data (attrition bias)	Low risk	Comment:
		Group 1/5/35
		Group 2: 5/35
Selective reporting (reporting bias)	Unclear risk	Comment: Trial registration not found. Outcomes pre‐specified in the methods section were reported in the results section
Other bias	Low risk	Comment: No other biases identified

Donnen et al. (1998)

**Table:** 

Methods	Design: RCT
	Unit: Individual
Participants	Location/Setting: Study was carried out in a rural setting in Katana health district, South Kivu, Democratic Republic of Congo
	Sample size: 900 hospitalised pre‐school children; 0–72 months of age
	Dropouts/Withdrawals: No loss to follow‐up
	Sex: Both male and female children were included
	Mean age: 55.5 months
	Inclusion criteria: Children aged 0–72 months hospitalised consecutively in the Lwiro Pediatric Hospital
	Exclusion criteria: Children were not eligible for inclusion in the study if they had been admitted to the hospital in a coma, if their parents or legal guardians had refused their participation, or if they had taken vitamin A capsules within the previous 4 months
Interventions	Intervention:
	Group 1: (*n *= 300)
	High dose Vitamin A, 200,000 IU or 100,000 IU (age < 12 months), on day of admission followed by placebo for every subsequent day until discharge
	Group 2: (*n *= 298)
	Low dose Vitamin A, 5,000 IU, on day of admission followed by placebo for every subsequent day until discharge
	Control: (*n *= 302)
	Placebo administered until discharge
	Duration of intervention: Until discharge
Outcomes	Primary outcomes: Morbidity and mortality
	Secondary outcomes: Duration of hospitalisation
	Timing of outcome assessment: Morbidity and weight data were gathered every day during hospitalisation, whereas height, mid‐upper arm circumference (MUAC), and serum retinol data were collected at baseline and after 7 and 30 days of hospitalisation
Notes	Study start date: March 1994
	Study end date: March 1996
	Funding source: Supported in part by a grant from the Fonds de la Recherche Scientifique et Médicale (contract 3.4505.94) and the David and Alice Van Buuren Foundation
	Conflicts of interest: Not specified

Risk of bias table

**Table:** 

Bias	Authors’ judgement	Support for judgement
Random sequence generation (selection bias)	Unclear risk	Quote: “…the paediatrician responsible for the study randomly assigned groups of 3 children to 1 of 3 treatment groups… This paediatrician was the only one with access to the allocation list”
		Comment: Insufficient information to permit judgement
Allocation concealment (selection bias)	Unclear risk	Quote: “…the paediatrician responsible for the study randomly assigned groups of 3 children to 1 of 3 treatment groups… This paediatrician was the only one with access to the allocation list”
		Comment: Insufficient information to permit judgement
Blinding of participants and personnel (performance bias)	Low risk	Quote: “The vitamin A and placebo solutions were identical in appearance… A person not affiliated with the study was the only one entrusted with the identification codes for the individually wrapped bottles of solution”
		Comment: Adequately done
Blinding of outcome assessment (detection bias)	Low risk	Quote: “The vitamin A and placebo solutions were identical in appearance… A person not affiliated with the study was the only one entrusted with the identification codes for the individually wrapped bottles of solution”
		Comment: Adequately done
Incomplete outcome data (attrition bias)	Low risk	Comment: No loss to follow‐up
Selective reporting (reporting bias)	Unclear risk	Comment: Trial registration not reported. Outcomes described in methodology section reported in results section
Other bias	Low risk	Comment: No other sources of bias identified

Fabiansen et al. (2017)

**Table:** 

Methods	Design: RCT
	Unit: Individual
Participants	Location/Setting: Study was carried out in a mixed setting in Province de Passore, Burkino Faso
	Sample Size: 1,609 children 6–23 months
	Dropouts/Withdrawals: 4 died, 61 were lost to follow‐up, and 119 were transferred out due to supplementation being switched to non‐experimental products
	Sex: Both male and female children were included
	Mean age: 11.5 months
	Inclusion criteria: Children were recruited if (a) they were resident in the catchment area at the time of inclusion, (b) a diagnosis of MAM was confirmed and (c) they were age 6–23 months
	Exclusion criteria: Children were not included if they were (a) treated for SAM or hospitalised within the past 2 months, (b) already in a nutritional programme or (c) requiring hospitalisation, for example, haemoglobin < 50 g/l. Children with a severe disability, limiting the feasibility of investigations, or with suspected allergy to milk, peanuts, CSB, or LNS were also excluded
Interventions	Intervention:
	Group 1: LNS (*n *= 809)
	Lipid Base Nutrient Supplement was given every 2 weeks for 12 weeks. Test diet provided 92 g/serving = 500 kcal·day^−1^·serving^−1^. LNSs were provided in sachets, each containing a daily serving ready for consumption
	Group 2: CSB (*n *= 800)
	Corn Soy Blend was given every 2 weeks for 12 weeks. Control diet provided 120 g/serving = 500 kcal·day^−1^·serving^−1^. CSBs were provided in bags of 1.7 kg containing a fortnightly ration (120 g·child^−1^·day^−1^ recommended to be divided in 3 meals, 40 g/meal) to be cooked with water and consumed as a porridge
	Intervention duration: 12 weeks
Outcomes	Primary outcomes: Fat‐free mass index accretion over 12 weeks
	Secondary outcomes: Recovery rate and additional anthropometric measures
	Timing of outcome assessment: After 12 weeks
Notes	Study start date: September 2013
	Study end date: August 2014
	Funding source: The study was funded by Danish International Development Assistance (09‐097LIFE) (KFM); MeÂdecins Sans Frontières (Denmark, Norway); Arvid Nilsson's Foundation; The World Food Program, which was part of a donation to the World Food Program from the American people through the support of the U.S. Agency for International Development's Office of Food for Peace; the Alliance for International Medical Action; and the European Union's humanitarian aid funds, in partnership with Action Contre la Faim
	Conflicts of interest: One author (K. F. M.) has received research grants from U.S. Dairy Export Council and the Danish Dairy Research Foundation, and also has research collaboration with Nutriset, a producer of LNS products, and patent owner; one author (H. F.) has received research grants from ARLA Food for Health Centre, and also has research collaboration with Nutriset, a producer of LNS products, and patent owner; one author (A. B.) was the inventor of LNS, for which Nutriset has the patent, but abandoned claims to royalties in 2003. Other authors declare no financial relationships with any organisations

Risk of bias table

**Table:** 

Bias	Authors’ judgement	Support for judgement
Random sequence generation (selection bias)	Low risk	Quote: “Random sequences, in blocks of 12 or 24 and stratified by site, were created by a person not involved in the trial using Randomization.com”
		Comment: Adequately done
Allocation concealment (selection bias)	Low risk	Quote: “Supplements were designated by a 1‐letter code by the manufacturer, and a code‐key was kept in a sealed envelope in a safe until completion of data analysis”
		Comment: Adequately done
Blinding of participants and personnel (performance bias)	High risk	Quote: “The trial was double‐blinded with respect to soy quality and milk content, but not matrix”
		Comment: Not done
Blinding of outcome assessment (detection bias)	High risk	Quote: “The trial was double‐blinded with respect to soy quality and milk content, but not matrix”
		Comment: Not done
Incomplete outcome data (attrition bias)	Low risk	Comment:
		Group 1: 58/809
		Group 2: 64/800
Selective reporting (reporting bias)	Low risk	Comment: The trial was registered at ISRCTN registry as ISRCTN42569496 and pre‐specified outcomes have been reported
Other bias	Low risk	Comment: No other biases identified

Hossain et al. (2009)

**Table:** 

Methods	Design: Quasi‐experimental design (natural experiment)
	Unit: Not applicable
Participants	Location/Setting: Study was carried out in an urban setting in Dhaka, Bangladesh
	Sample size: 60 children; 2–59 months of age
	Dropouts/Withdrawals: No loss to follow‐up
	Sex: Both male and female children included
	Mean age: 18.11 months
	Inclusion criteria: Severely malnourished children, aged 2–59 months, whose weight for height was below 70% of the expected (NCHS/WHO references) with or without bilateral pitting edema were included in the study
	Exclusion criteria: Children with major congenital abnormalities or disabilities and having feeding difficulty were excluded
Interventions	Intervention:
	Group 1: (*n *= 30)
	Children were managed as per the WHO protocol in which the management of children with severe malnutrition was divided into 2 phases; initial, and rehabilitation phase and managed. Whole cow milk, soy oil and sugar (100 kcal·kg^−1^·day^−1^) was given 2 hourly
	Group 2: (*n *= 30)
	Children were managed as per the Institute of Child and Mother Health (ICMH) protocol with no phasing in the management. Whole cow milk, soy oil and sugar (100 kcal·kg^−1^·day^−1^) was given 2 hourly
	Duration of intervention: Till recovery
Outcomes	Primary outcomes: Clinical: improved appetite, disappearance of edema, improvement of other associated medical conditions. Catch‐up growth: weight gain in gram per kg per day. Time taken for gaining target weight: [weight for height reaching 1 *SD* (90%) of NCHS/WHO median reference values] calculated from admission weight using NCHS/WHO reference growth chart
	Secondary outcomes: Mortality rate
	Timing of outcome assessment: At recovery
Notes	Study start date: June 2003
	Study end date: December 2003
	Funding source: Institute of Child and Mother Health
	Conflicts of interest: None declared

Risk of bias table

**Table:** 

Bias	Authors’ judgement	Support for judgement
Random sequence generation (selection bias)	High risk	Quote: “Quasi‐experimental non‐randomized clinical trial”
		Comment: Not done
Allocation concealment (selection bias)	High risk	Quote: “Quasi‐experimental non‐randomized clinical trial”
		Comment: Not done
Blinding of participants and personnel (performance bias)	High risk	Comment: Not done
Blinding of outcome assessment (detection bias)	High risk	Comment: Not done
Incomplete outcome data (attrition bias)	Low risk	Comment: No loss to follow‐up
Selective reporting (reporting bias)	Unclear risk	Comment: Trial registration not specified. Outcomes specified in the methodology section were reported in the results section
Other bias	Low risk	Comment: No other biases identified

Hsieh et al. (2015)

**Table:** 

Methods	Design: RCT
	Unit: Individual
Participants	Location/Setting: Study was carried out in a rural setting in Katana health district, South Kivu, Democratic Republic of Congo
	Sample size: 141 children aged 6–59 months
	Dropouts/Withdrawals: Lost to follow 9, died 6
	Sex: Both male and female children were included
	Mean age: 19.5 months
	Inclusion criteria: Having a mid‐upper arm circumference <11.5 cm and/or bilateral pitting edema, who qualify for community‐based management of SAM. Appetite was assessed by giving the child 30 g of RUTF and requiring him/her to consume it within 20 min. Children with or without HIV
	Exclusion criteria: Children treated for SAM in the previous 6 months, the presence of a chronic, debilitating condition such as cerebral palsy or congenital heart disease, or peanut allergy. HIV infection was not an exclusion criterion
Interventions	Intervention:
	Group 1: HO‐RUTF (*n *= 71)
	High oleic peanut, palm oil and linseed oil given every 2 week for 12 weeks
	Group 2: RUTF (*n *= 70)
	Peanuts, palm oil + soy oil given every 2 week for 12 weeks
	Duration of intervention: 12 weeks or till recovery
Outcomes	Primary outcomes: Change in plasma DHA and EPA content after 4 weeks.
	Secondary outcomes: Rates of recovery, length and weight gain, and the change in plasma content of arachidonic acid. Recovery from SAM was defined as having a mid‐upper arm circumference > 12.4 cm without edema within 12 weeks of enrolment
	Timing of outcome assessment: after 12 weeks
Notes	Study start date: January 2014
	Study end date: May 2014
	Funding source: This study was supported by NIH grant R01 AT007003 from the National Center for Complementary and Integrative Health (NCCIH) and the Office of Dietary Supplements (ODS). The therapeutic foods were donated by Nutriset and Project Peanut Butter
	Conflicts of interest: None declared

Risk of bias table

**Table:** 

Bias	Authors’ judgement	Support for judgement
Random sequence generation (selection bias)	Low risk	Quote: “Subjects were randomised to either RUTF or HO‐RUTF by choosing a treatment designation in a sealed envelope, prepared by a study assistant who did not participate in the data collection or analysis”
		Comment: Adequately done
Allocation concealment (selection bias)	Low risk	Quote: “Subjects were randomised to either RUTF or HO‐RUTF by choosing a treatment designation in a sealed envelope, prepared by a study assistant who did not participate in the data collection or analysis”
		Comment: Adequately done
Blinding of participants and personnel (performance bias)	Low risk	Quote: “The children, caretakers, and clinic workers were blinded to the assigned intervention”
		Comment: Adequately done
Blinding of outcome assessment (detection bias)	Low risk	Quote: “The children, caretakers, and clinic workers were blinded to the assigned intervention”
		Comment: Adequately done
Incomplete outcome data (attrition bias)	Low risk	Comment: No loss to follow‐up
Selective reporting (reporting bias)	Low risk	Comment: The study was registered at ClinicalTrials.gov as NCT02053857 and pre‐specified outcomes reported
Other bias	Low risk	Comment: No other biases identified

Irena et al. (2015)

**Table:** 

Methods	Design: RCT
	Unit: Cluster
Participants	Location/Setting: Study was carried out in health care clinics run by the Lusaka District Health Management Team in Lusaka, Zambia
	Sample size: 1,927 children aged 6–59 months
	Dropouts/Withdrawals: 543 children lost to follow‐up
	Sex: Both male and female children were included
	Mean age: 17 months
	Inclusion criteria: Children aged between 6 and 59 months and had been diagnosed as suffering from SAM without complications. The diagnostic criteria for SAM was a mid‐upper arm circumference (MUAC) < 11.0 cmorpittingoedemaofgrade1(+)or 2(++). Complications were defined as either medical or the absence of appetite
	Exclusion criteria: Children with SAM who presented with complication were not eligible for this study. Children previously discharge from the study with a recovered outcome that later relapsed and presented again at one of the participating HCs with a new episode of SAM were also not eligible for enrolment in the study a second time
Interventions	Intervention:
	Group 1: P‐RUTF (*n *= 1103)
	Standard peanut‐based RUTF given daily till discharged
	Group 2: SMS‐RUTF (*n *= 824)
	Soybean/maize/sorghum grains given weekly till discharged
	Duration of intervention: Till discharge
Outcomes	Primary outcomes: Recovery (cure), death, default, transfer out of the catchment area and non‐recovery
	Secondary outcomes: Not specified
	Timing of outcome assessment: Till discharge
Notes	Study start date: June 2009
	Study end date: August 2010
	Funding source: Irish Aid (IA) provided funding for the study
	Conflicts of interest: Valid Nutrition designed and produced the SMSRUTF. One of the authors (V. O. O.) is an employee of Valid Nutrition. One of the authors (S. C.) is the unpaid director of Valid Nutrition.Valid International is the sister company of Valid Nutrition

Risk of bias table

**Table:** 

Bias	Authors’ judgement	Support for judgement
Random sequence generation (selection bias)	Low risk	Quote: “Using the sampling frame prepared by AHI, the epidemiologist (MBO) with no prior knowledge of the Lusaka programme, randomly allocated intervention arms to HCs in block of four using randomisation software”
		Comment: Adequately done
Allocation concealment (selection bias)	Unclear risk	Quote: “Using the sampling frame prepared by AHI, the epidemiologist (MBO) with no prior knowledge of the Lusaka programme, randomly allocated intervention arms to HCs in block of four using randomisation software”
		Comment: Insufficient information
Blinding of participants and personnel (performance bias)	High risk	Quote: “The study could not be blind because of the differences in packaging and taste between the SMS‐RUTF and the P‐RUTF”
		Comment: Not done
Blinding of outcome assessment (detection bias)	High risk	Quote: “The study could not be blind because of the differences in packaging and taste between the SMS‐RUTF and the P‐RUTF”
		Comment: Not done
Incomplete outcome data (attrition bias)	High risk	Comment:
		Group 1: 282/1103
		Group 2: 261/824
Selective reporting (reporting bias)	Unclear risk	Comment: Trial registration information not provided. Outcomes pre‐specified reported in the results section
Other bias	Low risk	Comment: No other biases identified

Isanaka et al. (2016)

**Table:** 

Methods	Design: RCT
	Unit: Individual
Participants	Location/Setting: Study was carried out in a rural setting in Madarounfa, Niger
	Sample Size: 2,412 children 6–59 months of age
	Dropouts/Withdrawals: 13 lost to follow‐up
	Sex: Both male and female children included
	Mean age: 16.7 months
	Inclusion criteria: All children presenting to the study centre who were candidates for outpatient treatment of severe acute malnutrition were eligible for inclusion if they lived within 15 km of the centre, were available for the 12‐week study period, had not been admitted to a nutritional program within the previous 3 months or received any antibiotic within the previous 7 days, had no clinical complications requiring antibiotic treatment, and had no congenital abnormalities
	Exclusion criteria: Not specified
Interventions	Intervention: (*n *= 1210)
	Twice daily treatment with a split‐dose of 80 mg/kg of body weight with amoxicillin. Duration of treatment was 1 week
	Control: (*n *= 1,202)
	Placebo administered two times per day for 1 week
	Duration of intervention: 12 week
Outcomes	Primary outcomes: Nutritional recovery by 8 weeks
	Secondary outcomes: Nonresponse at 8 weeks, death from any cause, default (defined as three or more consecutive missed weekly visits), and transfer to inpatient care
	Timing of outcome assessment: At 4, 8 and 12 weeks after study enrolment
Notes	Study start date: October 2012
	Study end date: November 2013
	Funding source: Supported by Médecins sans Frontières Operational Center Paris
	Conflicts of interest: None declared

Risk of bias table

**Table:** 

Bias	Authors’ judgement	Support for judgement
Random sequence generation (selection bias)	Low risk	Quote: “The randomisation codes were created with a computerized random‐number generator according to site”
		Comment: Adequately done
Allocation concealment (selection bias)	Low risk	Quote: “The codes were kept inside opaque, sealed, consecutively numbered envelopes; and opened by a study physician in numerical order”
		Comment: Adequately done
Blinding of participants and personnel (performance bias)	Low risk	Quote: “Amoxicillin and placebo were indistinguishable in colour and packaging. All clinical and research staff members were unaware of the treatment assignments”
		Comment: Adequately done
Blinding of outcome assessment (detection bias)	Low risk	Quote: “Amoxicillin and placebo were indistinguishable in colour and packaging. All clinical and research staff members were unaware of the treatment assignments”
		Comment: Adequately done
Incomplete outcome data (attrition bias)	Low risk	Comment:
		Intervention: 11/1210
		Control: 2/1202
Selective reporting (reporting bias)	Low risk	Comment: Trial registered at ClinicalTrials.gov number, NCT01613547 and outcomes described in methodology section were reported in results section
Other bias	Low risk	Comment: No other biases reported

Jones et al. (2015)

**Table:** 

Methods	Design: RCT
	Unit: Individual
Participants	Location/Setting: Study was carried out in a rural setting in Kilifi county, Kenya
	Sample Size: 60 children aged 6–50 months
	Dropouts/Withdrawals: 14 lost to follow‐up
	Sex: Both male and female children were included
	Mean age: 16 months
	Inclusion criteria: Participants were aged 6–60 months with SAM; had been medically and nutritionally stabilized, and were eligible to receive RUTF according to national guidelines
	Exclusion criteria: Children were excluded if they were HIV‐infected, undergoing treatment for tuberculosis, had other recognized or suspected major chronic inflammatory conditions (e.g., malignancy), or reported allergy or hypersensitivity to any of the product ingredients
Interventions	Intervention:
	Group 1: (*n *= 21)
	Standard peanut‐based RUTF (S‐RUTF)
	Group 2: (*n* = 20)
	A flax seed oil‐containing RUTF (F‐RUTF); Flax seed oil‐based RUTF given weekly; except oil was given for 2 weeks only later RUTF alone
	Group 3: (*n *= 20)
	Flax seed oil‐containing RUTF with additional fish oil capsules (FFO‐RUTF); given weekly
Outcomes	Primary outcomes: Erythrocyte *n* − 3 PUFA content
	Secondary outcomes: Safety and acceptability of the intervention; recovery and growth
	Timing of outcome assessment: At 3 months. Scheduled study follow‐up took place at days 7, 14, 21, 28, 56 and 84 after enrolment
Notes	Study start date: June 2012
	Study end date: July 2013
	Funding source: This study was funded by a grant from The Bill and Melinda Gates Foundation through the Grand Challenges Explorations initiative (OPP1046183) and by The Wellcome Trust via Fellowships to KDJJ (092088) and JAB (083579), which include salary support
	Conflicts of interest: One author (S. C.) is the non‐executive chairman of Valid Nutrition, a charity that is a commercial manufacturer of ready‐to‐use foods and manufactured the investigational RUTF products in this study. The other authors declared no competing interests

Risk of bias table

**Table:** 

Bias	Authors’ judgement	Support for judgement
Random sequence generation (selection bias)	Low risk	Quote: “A randomisation list was generated in STATA (version 12.0) with variable block sizes using the following code”
		Comment: Adequately done
Allocation concealment (selection bias)	Low risk	Quote: “The trial statistician prepared 60 opaque envelopes labelled with study numbers, inside each of which was a card identifying a four‐digit RUTF code and specifying ‘with fish oil’ or ‘without fish oil’“
		Comment: Adequately done
Blinding of participants and personnel (performance bias)	Low risk	Quote: “The trial was conducted double‐blind between the S‐RUTF and F‐RUTF arms and open label with respect to FFO‐RUTF“
		Comment: Adequately done
Blinding of outcome assessment (detection bias)	Low risk	Quote: “The trial was conducted double‐blind between the S‐RUTF and F‐RUTF arms and open label with respect to FFO‐RUTF“
		Comment: Adequately done
Incomplete outcome data (attrition bias)	High risk	Comment:
		Group 1: S‐RUTF: 3/21
		Group 2: F‐RUTF: 8/20
		Group 3: FFO‐RUTF: 3/20
Selective reporting (reporting bias)	Low risk	Comment: The trial is registered at Clinicaltrials.gov NCT01593969. and all pre‐specified outcomes were reported
Other bias	Low risk	Comment: No other biases

Karakochuk et al. (2012)

**Table:** 

Methods	Design: RCT
	Unit: Cluster
Participants	Location/Setting: Study was carried out in 10 health centres and health posts in the northern region of the Sidama zone, Ethiopia
	Sample size: 1,125 children aged 6–60 moths
	Dropouts/Withdrawals: 76 lost to follow‐up
	Sex: Both male and female children were enrolled
	Mean age: 35.3 months
	Inclusion criteria: Children with MUAC < 135 mm were referred for second‐stage assessment
	Exclusion criteria: (a) children with MUAC < 110 mm, bilateral pitting edema, or other complications; (b) children transferred from therapeutic feeding programs; and (c) children with any condition preventing safe ingestion of either food (i.e., peanut allergy)
Interventions	Intervention:
	Group 1: RUSF (*n *= 375)
	RUSF: Supplementary plumpy; Nutriset was given biweekly for 16 weeks
	Group 2: CSB (*n *= 750)
	CSB: Corn–Soy blend + veg oil (premix) was given biweekly for 16 weeks
	Duration of intervention: 16 weeks
Outcomes	Primary outcomes: Recovery
	Secondary outcomes: Defaulted, transferred, non‐response, mortality
	Timing of outcome assessment: 16 weeks
Notes	Study start date: April 2009
	Study end date: October 2009
	Funding source: Supported by the United Nations World Food Programme and Action Contre la Faim–France
	Conflicts of interest: None declared

Risk of bias table

**Table:** 

Bias	Authors’ judgement	Support for judgement
Random sequence generation (selection bias)	Low risk	Quote: “2 districts were randomly assigned to receive either CSB or RUSF by using a blinded draw from an opaque bag”
		Comment: Adequately done
Allocation concealment (selection bias)	Low risk	Quote: “2 districts were randomly assigned to receive either CSB or RUSF by using a blinded draw from an opaque bag”
		Comment: Adequately done
Blinding of participants and personnel (performance bias)	High risk	Comment: Not done
Blinding of outcome assessment (detection bias)	High risk	Comment: Not done
Incomplete outcome data (attrition bias)	Low risk	Comment:
		RUSF group: 24/375
		CSB group: 52/750
Selective reporting (reporting bias)	Low risk	Comment: This protocol was registered on the clinicaltrials.gov (NCT 01097889) and pre‐specified outcomes were reported
Other bias	Low risk	Comment: No other biases identified

LaGrone et al. (2012)

**Table:** 

Methods	Design: RCT
	Unit: Individual
Participants	Location/Setting: Study was carried out in a rural setting in South TFC, Malawi
	Sample size: 2,890 children aged 6–59 month
	Dropouts/Withdrawals: 178 children lost to follow‐up
	Sex: Both female and male children were enrolled
	Mean age: 19.4 months
	Inclusion criteria: Children aged 6–59 months with MAM were recruited at 18 rural therapeutic feeding clinics in southern Malawi
	Exclusion criteria: Children were excluded if they were simultaneously involved in another research trial or supplementary feeding program, had a chronic debilitating illness (not including HIV or tuberculosis), or had a history of peanut allergy. Children were also excluded if they had received therapy for acute malnutrition within 1 month before presentation so as to focus the study primarily on the initial treatment of MAM
Interventions	Intervention:
	Group 1: CSB++ (*n *= 948)
	Corn–soy blend++: Corn flour, soy flour, DSM, soy oil, min/vit was given every 2 weeks for 12 weeks
	Group 2: Soy RUSF (*n *= 964)
	Soy‐RUSF: Soy–peanut paste, soy oil, Min/Vit, Ca_2_PO_4_/CaCO_3_ was given every 2 weeks for 12 weeks
	Group 3: Soy/Whey RUSF (*n *= 978)
	Soy/Whey RUSF: Soy protein, peanut paste, whey, veg fat, malto‐dextrin, cocoa, Min/Vit (PlumpySup; NUTRISET) was given every 2 weeks for 12 weeks
	Duration of intervention: 12 weeks
Outcomes	Primary outcomes: Recovered, developed SAM, remained MAM, died, defaulted
	Secondary outcomes: Time to recovery, rate of adverse events (allergic reactions, vomiting and diarrhoea) and rates of gain in weight, length and MUAC
	Timing of outcome assessment: At 12 weeks and 1 year after the intervention.
Notes	Study start date: October 2009
	Study end date: December 2010
	Funding source: Supported by the Academy for Educational Development (AED) through the Office of Health, Infectious Disease, Nutrition, Bureau of Global Health, and the Office of Food for Peace, U.S. Agency for International Development, under the terms of Cooperative Agreement GHN‐A‐00‐08‐00001‐00 through the FANTA‐2 project operated by AED. IT was supported by NIH training grant T32‐HD049338. The micronutrients for the CSB++ were donated by the World Food Programme, Rome, Italy; the milk for CSB++ was donated by Arla Foods, Arhus, Denmark; soy RUSF was donated by Project Peanut Butter, Blantyre, Malawi; and soy/whey RUSF was donated by Nutriset, Malaunay, France
	Conflicts of interest: None declared

Risk of bias table

**Table:** 

Bias	Authors’ judgement	Support for judgement
Random sequence generation (selection bias)	Low risk	Quote: “A block randomisation list was created by using a computer random number generator”
		Comment: Adequately done
Allocation concealment (selection bias)	Low risk	Quote: “Allocation was performed by caregivers drawing opaque envelopes containing 1 of 9 coded letters corresponding to 1 of the 3 supplementary foods. This code was accessible only to the food distribution personnel, who did not assess participant outcomes or eligibility”
		Comment: Adequately done
Blinding of participants and personnel (performance bias)	High risk	Quote: “The investigators who performed the clinical assessments were blinded to the child's assigned food group. The children and caregivers could not be blinded because the 3 supplementary foods differed in taste, appearance, and preparation required”
		Comment: Not done
Blinding of outcome assessment (detection bias)	Low risk	Quote: “The investigators who performed the clinical assessments were blinded to the child's assigned food group. The children and caregivers could not be blinded because the 3 supplementary foods differed in taste, appearance, and preparation required”
		Comment: Adequately done
Incomplete outcome data (attrition bias)	Low risk	Comment:
		CSB: 60/948
		Soy RUSF: 58/964
		Soy/Whey RUSF: 60/978
Selective reporting (reporting bias)	Low risk	Comment: This trial is registered at clinicaltrials.gov as NCT00998517 and pre‐specified outcomes reported
Other bias	Low risk	Comment: No other biases found

Manary et al. (2004)

**Table:** 

Methods	Design: Quasi‐experimental study (allocation is not truly random)
	Unit: Not applicable
Participants	Location/Setting: Study was carried out in a nutrition unit in Blantyre, Malawi
	Sample size: 282 children aged 12–59 months
	Dropouts/Withdrawals: 47 children were lost to follow‐up
	Sex: Both male and female children were included
	Mean age: 28.6 months
	Inclusion criteria: All children (HIV Negative) discharged from the nutrition rehabilitation unit (NRU) from 25 January to 15 October 2001 at the Queen Elizabeth Central Hospital in Blantyre, Malawi aged greater than 12 months were eligible and included
	Exclusion criteria: HIV positive children were not included
Interventions	Intervention:
	Group 1: RTUF‐SUP (*n *= 96)
	High energy diet + supplement (peanut butter, milk powder, oil, sugar + min/vit) + MIN/VIT fort was given every 2 weeks for 16 weeks
	Group 2: MS (maize–soy) (*n* = 117)
	Maize (80%), Soy (20%) flour porridge was given every 2 weeks for 16 weeks
	Group 3: RTUF (*n *= 69)
	High energy diet (peanut butter, milk powder, sugar, oil) was given every 2 weeks for 16 weeks
	Duration of intervention: 16 weeks
Outcomes	Primary outcomes: Recovery rate, dropout, mortality, relapse
	Secondary outcomes: Weight gain, height gain, MUAC gain
	Timing of outcome assessment: At 16 weeks
Notes	Study start date: January 2001
	Study end date: October 2001
	Funding source: This work was supported by a grant from the Allen Foundation, and Craig and Benith MacPherson. The RTUF and RTUF supplement was donated by Nutriset, Malaunay, France
	Conflicts of interest: Not specified

Risk of bias table

**Table:** 

Bias	Authors’ judgement	Support for judgement
Random sequence generation (selection bias)	High risk	Quote: “Children discharged on days 1, 2, 7 or 8 were given maize/soy, children discharged on days 3, 4, 9 or 10 were given RTUF supplement, and those discharged on day 5 or 6 were given RTUF. The systematic allocation was devised by one of the investigators prior to the initiation of the study, and communicated prospectively to the study nurses who enrolled the children”
		Comment: Inadequately done
Allocation concealment (selection bias)	Unclear risk	Quote: “Children discharged on days 1, 2, 7 or 8 were given maize/soy, children discharged on days 3, 4, 9 or 10 were given RTUF supplement, and those discharged on day 5 or 6 were given RTUF. The systematic allocation was devised by one of the investigators prior to the initiation of the study, and communicated prospectively to the study nurses who enrolled the children”
		Comment: Insufficient information
Blinding of participants and personnel (performance bias)	High risk	Comment: The food formulations were different. Blinding not done
Blinding of outcome assessment (detection bias)	High risk	Comment: The food formulations were different. Blinding not done
Incomplete outcome data (attrition bias)	Low risk	Comment:
		Maize/soy group: 15/117
		RUTF group: 7/69
		RUTF supplement group: 25/96
Selective reporting (reporting bias)	Unclear risk	Comment: Trial registration not specified. but pre‐specified outcomes described in methodology section reported in the results section
Other bias	Low risk	Comment: No other biases identified

Manary et al. (2012)

**Table:** 

Methods	Design: RCT
	Unit: Individual
Participants	Location/Setting: Study was carried out in 18 feeding clinics in rural Malawi
	Sample size: 2767 children aged 6–59 months
	Dropouts/Withdrawals: 107 children lost to follow‐up
	Sex: Both male and female children were included
	Mean age: 21 months
	Inclusion criteria: Children who were 6 to 59 months of age, with edema (indicative of kwashiorkor), a weight‐for‐height *z*‐score of <−3 (indicative of marasmus), or both (marasmic kwashiorkor), were eligible for enrolment
	Exclusion criteria: Children who were too ill to consume the test dose
Interventions	Intervention:
	Group 1: (*n *= 924)
	Daily treatment with amoxicillin suspension of 80–90 mg/kg for initial 7 days of the therapy. Children were followed once every 2 weeks for a period of 12 weeks
	Group 2: (*n *= 923)
	Daily treatment with 14 mg/kg cefdinir suspension for initial 7 days of the therapy. Children were followed once every 2 weeks for a period of 12 weeks
	Group 3: (*n *= 920)
	Placebo administered daily for initial 7 days of the therapy. Children were followed once every 2 weeks for a period of 12 weeks
	Duration of intervention: Initial 7 days of the therapy
Outcomes	Primary outcomes: Recoevray rate; mortality
	Secondary outcomes: Weight gain, length gain, antibiotics rates of adverse events, and time to recovery
	Timing of outcome assessment: Every 2 weeks till 12 weeks
Notes	Study start date: December 2009
	Study end date: January 2011
	Funding source: Supported by a grant from the Hickey Family Foundation, a cooperative agreement (GHN‐A‐00‐08‐00001‐00) with the Academy for Educational Development Food and Nutrition Technical Assistance 2 project (through the Office of Health, Infectious Diseases, and Nutrition, Bureau of Global Health, and Food for Peace, United States Agency for International Development), and grants (T32‐HD049338, to Dr. Trehan; and UL1‐RR024992, for statistical consulting) from the National Institutes of Health
	Conflicts of interest: Not specified

Risk of bias table

**Table:** 

Bias	Authors’ judgement	Support for judgement
Random sequence generation (selection bias)	Low risk	Quote: “Block randomisation lists were created using a computerized random number generator in permuted blocks of 54”
		Comment: Adequately done
Allocation concealment (selection bias)	Low risk	Quote: “Participating children were allocated to their study arm when their caregivers drew an opaque envelope containing one of nine coded letters corresponding to one of the three medication groups. The code was accessible only to specific pharmacy personnel at each clinic dedicated to the preparation and distribution of the medications. The medications and placebo were distributed in opaque plastic bottles with plastic syringes marked to indicate the dose of medication each child was to receive”
		Comment: Adequately done
Blinding of participants and personnel (performance bias)	Low risk	Quote: “The medications and placebo were distributed in opaque plastic bottles with plastic syringes marked to indicate the dose of medication each child was to receive…Caregivers, study nurses, and all study personnel involved in clinical assessments and data analysis were kept blinded to the intervention each child received”
		Comment: Adequately done
Blinding of outcome assessment (detection bias)	Low risk	Quote: “Caregivers, study nurses, and all study personnel involved in clinical assessments and data analysis were kept blinded to the intervention each child received”
		Comment: Adequately done
Incomplete outcome data (attrition bias)	Low risk	Comment:
		Amoxicillin group: 28/952
		Cefdinir group: 40/963
		Placebo: 39/959
Selective reporting (reporting bias)	Low risk	Comment: Trial registered at ClinicalTrials.gov number, NCT01000298 and pre‐specified outcomes were reported
Other bias	Low risk	Comment: No other biases identified

Matilsky et al. (2009)

**Table:** 

Methods	Design: RCT
	Unit: Individual
Participants	Location/Setting: Study was carried out in a rural setting in southern region of Malawi
	Sample size: 1,362 children aged 6–60 months
	Dropouts/Withdrawals: 40 lost to follow‐up
	Sex: Both male and female children were included
	Mean age: 19.7 months
	Inclusion criteria: Children with moderate wasting according to the WHO's current standards and with a good appetite were eligible for the study
	Exclusion criteria: Children who had signs of severe malnutrition, chronic illness, cardiac disease, congenital abnormalities, cancer or those who had been discharged from the nutritional rehabilitation unit, were not eligible for the study
Interventions	Intervention:
	Group 1: (*n *= 465)
	Milk/peanut fortified spread (Nutriset) given every 2 weeks for 8 weeks
	Group 2: (*n* = 450)
	Soy/peanut fortified spread (Nutriset) given every 2 weeks for 8 weeks
	Group 3: (*n *= 447)
	Corn–soy blend given every 2 weeks for 8 weeks
	Intervention duration: For 8 weeks
Outcomes	Primary outcomes: Recovery (defined as having a WHZ < −2)
	Secondary outcomes: Rates of gain in weight, stature and mid‐upper arm circumference (MUAC), and the development of adverse outcomes such as severe malnutrition or death
	Timing of outcome assessment: At 8 weeks
Notes	Study start date: July 2007
	Study end date: February 2008
	Funding Source: Support provided to the Food and Nutrition Technical Assistance (FANTA) Project by the Office of HIV/AIDS (OHA) and the Office of Health, Infectious Diseases and Nutrition (HIDN) of the Bureau of Global Health at the Agency for International development, under terms of Cooperative Agreement No. HRN‐A‐00‐98‐00046‐00 awarded to the Academy for Educational Development (AED)
	Conflicts of interest: None declared

Risk of bias table

**Table:** 

Bias	Authors’ judgement	Support for judgement
Random sequence generation (selection bias)	Low risk	Quote: “Caretakers chose an envelope that contained 1 of 6 letters and this letter was recorded separately from the child's clinical measurements”
		Comment: Adequately done
Allocation concealment (selection bias)	Low risk	Quote: “Each of the 6 letters corresponded to 1 of the 3 diets. A research assistant not involved in the study implemented the randomisation process”
		Comment: Adequately done
Blinding of participants and personnel (performance bias)	Low risk	Quote: “…investigators were unaware of the type of food each child was receiving during the study”
		Comment: Adequately done
Blinding of outcome assessment (detection bias)	Low risk	Quote: “Field workers and investigators remained unaware of the type of food each child received for the duration of the study”
		Comment: Adequately done
Incomplete outcome data (attrition bias)	Low risk	Comment:
		Group 1: 19/465
		Group 2: 24/450
		Group 3: 17/447
Selective reporting (reporting bias)	Low risk	Comment: The trial was registered with Current Controlled Trials Ltd and pre‐specified outcomes were reported
Other bias	Low risk	Comment: No other biases reported

Maust et al. (2015)

**Table:** 

Methods	Design: RCT
	Unit: Cluster
Participants	Location/Setting: Study was carried out in Sierra Leone conducted in 10 centres treating global acute malnutrition in children
	Sample size: 1,957 children aged 6–59 months
	Dropouts/Withdrawals: 159 children lost to follow‐up
	Sex: Both male and female children were included
	Mean age: 14.1 months
	Inclusion criteria: Children aged 6–59 months with a mid‐upper arm circumference (MUAC) < 12.5 cm or bipedal edema and an adequate appetite who presented to 1 of 10 clinics in Port Loko District of Sierra Leone.When more than one child from the same household was malnourished, only the youngest child was enrolled in the study
	Exclusion criteria: Children with known chronic health conditions such as cerebral palsy or congenital deformities, children who had participated in a supplementary or therapeutic feeding program within the previous month, children with a history of peanut allergy, children without an adequate appetite
Interventions	Intervention:
	Group 1—Integrated: (*n *= 1,100)
	Children with SAM were given RUTF (175 kcal·kg^−1^·day^−1^) and amoxicillin every 2 weeks for 12 weeks. Children with MAM were given RITF (75 kcal·kg^−1^·day^−1^) every 2 weeks for 12 weeks
	Group 2 ‐Standard: (*n *= 857)
	Children with SAM were given RUTF (200 kcal·kg^−1^·day^−1^) and amoxicillin every 2 weeks for 12 weeks. Children with MAM were given super cereal plus—a fortified flour of CSB with oil and milk powder (1,250 kcal/day) every 2 weeks for 12 weeks
	Children in both groups managed for sickness per WHO protocol: electrolytes, multivitamins, nutrients and antibiotics administered
	Duration of intervention: 12 weeks
Outcomes	Primary outcomes: Coverage and recovery rate
	Secondary outcomes: Duration of treatment, rates of weight and MUAC gain, clinical status 6 months after recovery and cost of foodstuffs used
	Timing of outcome assessment: At 12 weeks
Notes	Study start date: February 2013
	Study end date: November 2013
	Funding source: Supported by the CDC (grant 1U01GH000647‐01)
	Conflicts of interest: None declared

Risk of bias table

**Table:** 

Bias	Authors’ judgement	Support for judgement
Random sequence generation (selection bias)	Low risk	Quote: “The sites were randomly assigned to deliver either integrated or standard management of acute malnutrition with the use of a random number generator by a study aid without knowledge of the characteristics of study sites”
		Comment: Adequately done
Allocation concealment (selection bias)	Unclear risk	Comment: Insufficient information
Blinding of participants and personnel (performance bias)	High risk	Quote: “This was a cluster‐randomised, unblinded, controlled clinical trial comparing the integrated management of GAM with standard management of MAMand SAM”
		Comment: Not done
Blinding of outcome assessment (detection bias)	High risk	Quote: “This was a cluster‐randomised, unblinded, controlled clinical trial comparing the integrated management of GAM with standard management of MAMand SAM”
		Comment: Not done
Incomplete outcome data (attrition bias)	Low risk	Comments:
		Integrated group: 90/110
		Standard group: 69/857
Selective reporting (reporting bias)	Low risk	Comment: This trial was registered at clinicaltrials.gov as NCT01785680 and pre‐specified outcomes were reported
Other bias	Low risk	Comment: No other biases identified

Medoua et al. (2015)

**Table:** 

Methods	Design: RCT
	Unit: Individual
Participants	Location/Setting: Study was carried out in Health districts of Mvog‐Beti and Evodoula in the Centre region of Cameroon
	Sample size: 81 children aged 6–59 months
	Dropouts/Withdrawals: No loss to follow‐up
	Sex: Both male and female children were included
	Mean age: 24.6 months
	Inclusion criteria: Malnourished children (weight‐for‐height *z*‐score between −3 and −2) were selected
	Exclusion criteria: Children were excluded if they did not have appetite, had a chronic debilitating
	illness, or had a history of peanut allergy
Interventions	Intervention:
	Group 1: CSB+ (*n *= 41)
	Improved corn–soy blend: corn, soya, sugar, min/vit + soy oil was given every 2 weeks for 16 weeks. Treatment diet provided 40 kcal·kg^−1^·day^−1^
	Group 2: RUSF (*n *= 40)
	Ready‐to‐use supplementary food: Soya, corn flour, peanut paste, sugar, soy oil, min/vit was given every 2 weeks for 16 weeks. Control diet provided 40 kcal·kg^−1^·day^−1^
	Duration of intervention: 56 days
Outcomes	Primary outcomes: Recovery rate
	Secondary outcomes: Time to recovery and rates of gain in weight and mid‐upper arm circumference
	Timing of outcome assessment: Every 14 days till 56 days
Notes	Study start date: February 2012
	Study end date: July 2012
	Funding source: Supported by the International Atomic Energy Agency (Technical Cooperation project CMR/6/010)
	Conflicts of interest: None declared

Risk of bias table

**Table:** 

Bias	Authors’ judgement	Support for judgement
Random sequence generation (selection bias)	Low risk	Quote: “A randomisation list was created using a random number generator (Stat Trek)”
		Comment: Adequately done
Allocation concealment (selection bias)	Low risk	Quote: “Allocation to either CSB+ or RUSF was performed by caregivers drawing from an opaque bag containing coded numbers corresponding to one of the two supplementary foods”
		Comment: Adequately done
Blinding of participants and personnel (performance bias)	Low risk	Quote: “Investigators performing the clinical assessment and nutrition education were blinded to the child's assigned food group”
		Comment: Adequately done
Blinding of outcome assessment (detection bias)	Low risk	Quote: “Investigators performing the clinical assessment and nutrition education were blinded to the child's assigned food group”
		Comment: Adequately done
Incomplete outcome data (attrition bias)	Low risk	Comment: No loss to follow‐up
Selective reporting (reporting bias)	Low risk	Comment: Trial was registered at www.clinicaltrials.gov as NCT01898871 and pre‐specified outcomes were reported
Other bias	Low risk	Comment: No other biases identified

Mishra et al. (2019)

**Table:** 

Methods	Design: RCT
	Unit: Individual
Participants	Location/Setting: Study was carried out at paediatrics ward of SCB Medical College, Cuttack, India
	Sample Size: 120 children aged 6–60 months
	Dropouts/Withdrawals: No loss to follow‐up
	Sex: Both male and female children were included
	Mean age: Not specified
	Inclusion Criteria: SAM children aged 6–60 months. Severe acute malnutrition or severe wasting as defined by WHO criteria includes (a) very low weight for height (<70% of expected or below −3 *SD* scores for the median WHO standards) and/or (b) Visible wasting and/or (c) by the presence of nutritional edema and/or (d) mid‐upper arm circumference < 115 mm
	Exclusion criteria: Not specified
Interventions	Intervention:
	Group 1: Locally prepared ready‐to‐use therapeutic food (LRUTF) (*n *= 60)
	The study cohort received LRUTF diet. Subjects received a total of 6 feeds per day which included 3 feeds of LRUTF and 3 feeds from family pot amounting to an intake of approximately 150 kcal/kg/day and 1.5–2 g/kg of protein to both groups. Local ready to use therapeutic food (LRUTF) was prepared from groundnut, milk powder, sugar and vegetable oil
	Group 2: F100 (*n *= 60)
	The control cohort received F100 diet. Children received a total of 6 feeds per day which included 3 feeds of F100 and 3 feeds from family pot amounting to an intake of approximately 150 kcal/kg/day and 1.5–2 g/kg of protein. Diet was prepared locally using cow milk, sugar, vegetable oil and water
	Duration of intervention: Till recovery
Outcomes	Primary outcomes: Wt gain, recovery rate and length of stay
	Secondary outcomes: Anthropometric determinants (weight, height, MUAC), clinical determinants (wasting, oedema, death)
	Timing of Outcome Assessment: Followed up every 15 days till they reach weight of 1 *SD* below mean for height
Notes	Study start date: October 2015
	Study End Date: September 2017
	Funding source: Not specified
	Conflicts of interest: Not specified

Risk of bias table

**Table:** 

Bias	Authors’ judgement	Support for judgement
Random sequence generation (selection bias)	Unclear risk	Comment: Insufficient information
Allocation concealment (selection bias)	Unclear risk	Comment: Insufficient information
Blinding of participants and personnel (performance bias)	Unclear risk	Comment: Insufficient information
Blinding of outcome assessment (detection bias)	Unclear risk	Comment: Insufficient information
Incomplete outcome data (attrition bias)	Low risk	Comment: No loss to follow‐up
Selective reporting (reporting bias)	Unclear risk	Comment: Trial registration not specified. Outcomes mentioned in the methods section were reported in the results section
Other bias	Low risk	Comment: No other biases identified

Nackers et al. (2010)

**Table:** 

Methods	Design: RCT
	Unit: Individual
Participants	Location/Setting: The study was conducted in two Supplementary Feeding Centres (SFCs) in the remote and difficult‐to‐access villages of Mallawa and Bangaza (Magaria department, Zinder region, South of Niger)
	Sample size: 807 children aged 6–59 months
	Dropouts/Withdrawals: 53 children lost to follow‐up
	Sex: Both male and female children were included
	Mean age: Not specified
	Inclusion criteria: All children measuring 65 to <110 cm (used as a proxy for the age of 6–59 months), newly admitted to the Mallawa and Bangaza SFCs with MAM and good appetite were eligible for inclusion
	Exclusion criteria: Children requiring hospitalisation as well as those who had been hospitalised or admitted in a nutritional programme in the previous 2 months were excluded. Also, children with a MUAC < 135 mm and apparently healthy were not admitted to the Supplementary Feeding program
Interventions	Intervention:
	Group 1: CSB (*n *= 406)
	Corn–soy blend premix + veg oil + sugar was given weekly for 16 weeks
	Group 2: RUTF‐Nutriset (*n *= 401)
	(PlumpyNut) peanut, powder milk, veg oil, vit/min mix was given weekly for 16 weeks.
	Duration of intervention: 16 weeks
Outcomes	Primary outcomes: Weight gain and the recovery rate
	Secondary outcomes: Transfer to the I‐TFC, mortality, non‐responder and defaulter rates. Length of stay, MUAC gain and haemoglobin gain during treatment, relapse and height gain 6 months after discharge
	Timing of outcome assessment: 6 months after discharge
Notes	Study start date: August 2007
	Study end date: July 2008
	Funding source: Not specified
	Conflicts of interest: Not specified

Risk of bias table

**Table:** 

Bias	Authors’ judgement	Support for judgement
Random sequence generation (selection bias)	Low risk	Quote: “The allocation sequence (blocks of 10) was computer generated and concealed in sealed envelopes”
		Comment: Adequately done
Allocation concealment (selection bias)	Low risk	Quote: “The allocation sequence (blocks of 10) was computer generated and concealed in sealed envelopes”
		Comment: Adequately done
Blinding of participants and personnel (performance bias)	High risk	Comment: Not done
Blinding of outcome assessment (detection bias)	High risk	Comment: Not done
Incomplete outcome data (attrition bias)	Low risk	Comment:
		RUTF:27/401
		CSB: 26/406
Selective reporting (reporting bias)	Unclear risk	Comment: Trial registration not specified. Outcomes described in methodology section reported in results section
Other bias	Low risk	Comment: No other biases identified

Nikièma et al. (2014)

**Table:** 

Methods	Design: RCT
	Unit: Cluster
Participants	Location/Setting: Study was carried out in a rural setting in Hounde, Burkino Faso
	Sample size: 1,974 children aged 6–24 months of age
	Dropouts/Withdrawals: 83 lost to follow‐up
	Sex: Both male and female children were included
	Mean age: 13.4 (±4.6) months
	Inclusion criteria: Children aged 6–24 months, with uncomplicated MAM (WHZ < −2 and >−3 based on the 2006 WHO growth reference) and living in the catchment area of a health centre were cumulatively included in the trial
	Exclusion criteria: Children with a diagnosis of SAM (presence of pitting edema or WHZ < −3, without complications) were excluded from the trial
Interventions	Intervention:
	Group 1: Child centred counselling (CCC) (*n *= 605)
	Only Education Counselling was given weekly for 12 weeks. No supplementation was provided
	Group 2: Corn–soy blend (CSB++) (*n *= 675)
	Maize, soybean, milk soyoil, vit/min mix‐based diet was provided weekly for 12 weeks
	Group 3: Ready‐to‐use supplementary food (RUSF) (*n *= 694)
	Peanut butter, veg oil, whole soybean, shea butter, micronutrient based diet was provided weekly for 12 weeks
	Duration of intervention: 12 weeks
Outcomes	Primary outcomes: The primary outcome of the study was clinical status, defined as recovered, failed, died or dropped out
	Secondary outcomes: Attendance, time to recovery, weight, length, and daily MUAC gains
	Timing of outcome assessment: At 12 weeks
Notes	Study start date: July 2010
	Study end date: November 2011
	Funding source: Supported by Global Alliance for Improved Nutrition, the World Food Program and Nutrition Third World. Michiels Fabrieken (Belgium) donated the CSB++ for the pilot phase
	Conflicts of interest: None declared

Risk of bias table

**Table:** 

Bias	Authors’ judgement	Support for judgement
Random sequence generation (selection bias)	Low risk	Quote: “Random allocation was performed in public by the heads of each health centre who were invited to draw 1 paper from a basket containing 18 pieces of paper (6 papers for each of the study arms). This was done under the supervision of the principal investigator during the launch meeting”
		Comment: Adequately done
Allocation concealment (selection bias)	Unclear risk	Quote: “Random allocation was performed in public by the heads of each health centre who were invited to draw 1 paper from a basket containing 18 pieces of paper (6 papers for each of the study arms). This was done under the supervision of the principal investigator during the launch meeting”
		Comment: Insufficient information
Blinding of participants and personnel (performance bias)	High risk	Quote: “In the first arm (CCC), trained health workers provided weekly personalized counselling to caretakers. In the 2 other arms, children received weekly either 455 g CSB++ or 350 g locally produced soy‐based RUSF”
		Comment: Not done
Blinding of outcome assessment (detection bias)	High risk	Quote: “In the first arm (CCC), trained health workers provided weekly personalized counselling to caretakers. In the 2 other arms, children received weekly either 455 g CSB++ or 350 g locally produced soy‐based RUSF”
		Comment: Not done
Incomplete outcome data (attrition bias)	Low risk	Comment:
		CCC: 50/605
		CSB: 18/675
		RUSF: 15/694
Selective reporting (reporting bias)	Low risk	Comment: This trial was registered at clinicaltrials.gov as NCT01115647 and all pre‐specified outcomes were reported
Other bias	Low risk	Comment: No other biases identified

Oakley et al. (2010)

**Table:** 

Methods	Design: RCT
	Unit: Individual
Participants	Location/Setting: Study was carried out in a rural setting in southern Region of Malawi
	Sample size: 1,874 children aged 6–59 months
	Dropouts/Withdrawals: 51 children lost to follow‐up
	Sex: Both male and female children were included
	Mean age: 19.3 months
	Inclusion criteria: Eligible children were those with SAM and a good appetite. SAM was defined as having a weight‐for‐height *z*‐score (WHZ) <−3 and/or having bipedal pitting edema
	Exclusion criteria: Children known to have chronic illness, including HIV, cardiac disease, congenital abnormalities, cerebral palsy or cancer, or those who had participated in a treatment program for SAM within the past 12 weeks were not eligible for the study
Interventions	Intervention:
	Group 1: RUTF‐10% (*n *= 929)
	RUTF‐10%: Skimmed milk(10%), whole soy flour (15%), peanut paste, min/vit mix was provided every 2 weeks for 8 weeks
	Group 2: RUTF‐25% (*n *= 945)
	RUTF‐25%: Skim milk (25%), peanut paste, min/vit mix was given weekly for 8 weeks
	Duration of intervention: 8 weeks
Outcomes	Primary outcomes: Recovery
	Secondary outcomes: Rates of weight and height gain
	Timing of outcome assessment: At 8 weeks
Notes	Study start date: July 2008
	Study end date: April 2009
	Funding source: Supported by the Hickey Family Foundation. This study was also supported by the Office of Health, Infectious Disease, and Nutrition, Bureau for Global Health, United States Agency for International Development (USAID), under terms of Cooperative Agreement No. GHN‐A‐00‐08‐00001‐00, through the FANTA‐2 Project, operated by the Academy for Educational Development (AED)
	Conflicts of interest: None declared

Risk of bias table

**Table:** 

Bias	Authors’ judgement	Support for judgement
Random sequence generation (selection bias)	Low risk	Quote: “Randomization was blocked for the entire study rather than at each study site. To allocate children to a food group, caretakers chose a sealed envelope that contained 1 of 6 letters: 3 of these letters corresponded to the 25% milk formulation and 3 to the 10% milk formulation”
		Comment: Adequately done
Allocation concealment (selection bias)	Low risk	Quote: “To allocate children to a food group, caretakers chose a sealed envelope that contained 1 of 6 letters: 3 of these letters corresponded to the 25% milk formulation and 3 to the 10% milk formulation”
		Comment: Adequately done
Blinding of participants and personnel (performance bias)	Low risk	Quote: “Caretakers, field workers, and investigators assessing the children remained unaware of what type of food each child received for the duration of the study”
		Comment: Adequately done
Blinding of outcome assessment (detection bias)	Low risk	Quote: “Investigators assessing the children remained unaware of what type of food each child received for the duration of the study”
		Comment: Adequately done
Incomplete outcome data (attrition bias)	Low risk	Comment:
		RUTF 25%: 28/945
		RUTF 10%: 23/929
Selective reporting (reporting bias)	Low risk	Comment: This trial was registered at Current Controlled Trials (UK) as ISRCTN54186063 and pre‐specified outcomes were reported
Other bias	Low risk	Comment: No other biases identified

Phuka et al. (2009)

**Table:** 

Methods	Design: RCT
	Unit: Individual
Participants	Location/Setting: Study was carried out in a rural setting in Lungwena, Mangochi District, Malawi
	Sample size: 176 children aged 6–18 months
	Dropouts/Withdrawals: 6 lost to follow‐up
	Sex: Both male and female children were included
	Mean age: 11.6 months
	Inclusion criteria: Inclusion criteria for the trial included age of at least 6 months but less than 15 months, low WAZ (WAZ < −2.0), assumed residence in the study area throughout the follow‐up period and signed informed consent from at least one authorized guardian
	Exclusion criteria: Exclusion criteria were severe wasting, weight‐for‐length *z*‐score (WLZ < −3.0), presence of oedema, history of peanut allergy, severe illness warranting hospitalisation on the enrolment day, concurrent participation in another clinical trial or any symptoms of food intolerance within 30 min after the ingestion of a 6‐g test dose of FS, one of the food supplements used in the trial (given to all potential participants to exclude the possibility of peanut allergy)
Interventions	Intervention:
	Group 1: LP fortified (*n *= 86)
	Fortified‐LP: Maize flour, soya flour, micronutrients was given (71 g/day) weekly for 12 weeks
	Group 2: RUFS (*n *= 90)
	RUFS (NUTRISET): Maize flour‐peanut butter, milk, veg oil, micronutrients was given (50 g/day) weekly for 12 weeks
	Duration of intervention: 12 weeks
Outcomes	Primary outcomes: Weight gain
	Secondary outcomes: Length gain, mean change in anthropometric indices WAZ, LAZ and WLZ, recovery from moderate underweight, stunting, wasting, change in MUAC and change in blood haemoglobin concentration
	Timing of outcome assessment: At 12 weeks
Notes	Study start date: December 2004
	Study end date: July 2005
	Funding source: The trial was funded by grants from the Academy of Finland (grants 200720 and 109796), Foundation for Paediatric Research in Finland and Medical Research Fund of Tampere University Hospital. The micronutrient mixture used in the production of FS was provided free of charge by Nutriset, Inc. (Malaunay, France)
	Conflicts of interest: One of the authors (André Briend) was a consultant to Nutriset until December 2003 and the company has also financially supported the planning of another research project by the same study team through Per Ashorn and the University of Tampere after the completion of this trial. Other authors declare no conflict of interest

Risk of bias table

**Table:** 

Bias	Authors’ judgement	Support for judgement
Random sequence generation (selection bias)	Low risk	Quote: “The actual randomisation was done with a tailor‐made computer program, using random number and rank functions of a Microsoft Excel spreadsheet”
		Comment: Adequately done
Allocation concealment (selection bias)	Low risk	Quote: “For group allocation, consenting guardians of eligible participants were shown and asked to pick one from a set of 10 identical opaque envelopes containing information on the group allocation of the participant. Because one set had to be finished before using the next, for each block the first guardian chose one from a total of 10, the second chose one from a total of 9 and the 10th picked the last envelope”
		Comment: Adequately done
Blinding of participants and personnel (performance bias)	High risk	Quote: “…Ingredients of LP were maize flour, soy flour and micronutrients and those of FS were peanut butter, milk, vegetable oil, sugar and micronutrients”
		Comment: Not done
Blinding of outcome assessment (detection bias)	Low risk	Quote: “All measurements were done in triplicate by one author (J. P.), whose measurement reliability was assessed at the start of the study and who was blinded of the participant study allocation from enrolment to the end of follow‐up”
		Comment: Adequately done
Incomplete outcome data (attrition bias)	Low risk	Comment:
		Group 1 (LP): 2/86
		Group 2 (FS): 4/90
Selective reporting (reporting bias)	Low risk	Comment: Key details of the protocol were published at the clinical trial registry of the National Library of Medicine, Bethesda, MD, USA (https://www.clinicaltrials.gov, trial identification is NCT00131222) and pre‐specified outcomes described in methodology section were reported in results section
Other bias	Low risk	Comment: No other biases identified

Puett et al. (2012)

**Table:** 

Methods	Design: Cost‐effectiveness study using an activity‐based cost model and a societal perspective
	Unit: Not applicable
Participants	Location/Setting: Study was carried out in a rural setting in Bhola District, Bangladesh
	Sample size: Not applicable
	Dropouts/Withdrawals: Not applicable
	Sex: Not applicable
	Mean age: Not applicable
	Inclusion criteria: Not applicable
	Exclusion criteria: Not applicable
Interventions	This study assessed the cost‐effectiveness of adding the community‐based management of severe acute malnutrition (CMAM) to a community‐based health and nutrition programme delivered by community health workers (CHWs) in southern Bangladesh. The cost‐effectiveness of this model of treatment for severe acute malnutrition (SAM) was compared with the cost‐effectiveness of the “standard of care” for SAM (i.e., inpatient treatment), augmented with community surveillance by CHWs to detect cases, in a neighbouring area
Outcomes	Primary outcomes: Cost‐effectiveness
	Secondary outcomes: Not applicable
	Timing of outcome assessment: Not applicable
Notes	Study start date: March 2010
	Study end date: April 2010
	Funding source: This research was supported by funding from GAIN, the Global Alliance for Improved Nutrition. Additional support was provided by the Feinstein International Center at Tufts University
	Conflicts of interest: None declared

Risk of bias table

**Table:** 

Bias	Authors’ judgement	Support for judgement
Random sequence generation (selection bias)	Unclear risk	
Allocation concealment (selection bias)	Unclear risk	
Blinding of participants and personnel (performance bias)	Unclear risk	
Blinding of outcome assessment (detection bias)	Unclear risk	
Incomplete outcome data (attrition bias)	Unclear risk	
Selective reporting (reporting bias)	Unclear risk	
Other bias	Unclear risk	

Sandige et al. (2004)

**Table:** 

Methods	Design: Quasi‐experimental study (allocation is not truly random)
	Unit: Not applicable
Participants	Location/Setting: Study was carried out in Blantyre, Malawi
	Sample size: 182 children aged 1–5 years
	Dropouts/Withdrawals: 4 children lost to follow‐up
	Sex: Both male and female children were included
	Mean age: 28 months
	Inclusion criteria: All children aged 1–5 years discharged from the nutritional rehabilitation unit (NRU) at Queen Elizabeth Central Hospital in Blantyre, Malawi, were eligible
	Exclusion criteria: Not specified
Interventions	Intervention:
	Group 1: RUTF‐L: (*n *= 99)
	Local RUTF composed of full fat milk powder, icing sugar, cotton seed oil, peanut butter and a mineral–vitamin mixture was given every 2 weeks for 16 weeks or until target weight was achieved. A follow‐up visit was conducted 6 months after therapy completion to assess nutritional status
	Group 2: RUTF‐I (*n *= 83)
	Imported plumyNut (Nutriset) was given every 2 weeks for 16 weeks or until target weight was achieved. A follow‐up visit was conducted 6 months after therapy completion to assess nutritional status
	Duration of intervention: 14 weeks
Outcomes	Primary outcomes: Recovery
	Secondary outcomes: Weight gain, statural growth, growth in MUAC, anthropometric status 6 months after discharge from home‐based therapy and the prevalence of fever, cough and diarrhoea during the first 2 weeks of therapy
	Timing of outcome assessment: At 14 weeks
Notes	Study start date: April 2002
	Study end date: August 2002
	Funding source: Supported by gifts from Roger and Fran Koch, the Georgia Peanut Commission, and Frank and Mary Hellwig. Nutriset donated the imported RTUF for the study
	Conflicts of interest: Not specified

Risk of bias table

**Table:** 

Bias	Authors’ judgement	Support for judgement
Random sequence generation (selection bias)	High risk	Quote: “Children were assigned to one of the RTUF groups by systematic allocation according to order of entry into the project, with even numbered entries receiving locally produced RTUF and odd numbered entries receiving imported RTUF”
		Comment: Not adequate
Allocation concealment (selection bias)	High risk	Quote: “Children were assigned to one of the RTUF groups by systematic allocation according to order of entry into the project, with even numbered entries receiving locally produced RTUF and odd numbered entries receiving imported RTUF”
		Comment: Not adequate
Blinding of participants and personnel (performance bias)	High risk	Comment: “Because the packaging of the food was not identical, the trial was not blinded. The imported RTUF was given in 92‐g disposable foil sachets, whereas the locally produced RTUF was given in 275‐g clear plastic jars with screw lids”
		Quote: Not adequate
Blinding of outcome assessment (detection bias)	High risk	Comment: “Because the packaging of the food was not identical, the trial was not blinded. The imported RTUF was given in 92‐g disposable foil sachets, whereas the locally produced RTUF was given in 275‐g clear plastic jars with screw lids”
		Quote: Not adequate
Incomplete outcome data (attrition bias)	Low risk	Comments:
		RUTF‐L: 2/99
		RUTF‐I: 2/83
Selective reporting (reporting bias)	Low risk	Comment: Trial not registered; however outcomes described in methodology section were reported in results section
Other bias	Low risk	Comment: No other biases identified

Sattar et al. (2012)

**Table:** 

Methods	Design: RCT
	Unit: Individual
Participants	Location/Setting: Study was carried out in an urban/peri‐urban setting in Dhaka, Bangladesh
	Sample size: 260 children aged 6–59 months of age
	Dropouts/Withdrawals: 53 lost to follow‐up
	Sex: Both male and female children were included
	Mean age: 16 (±10) months
	Inclusion criteria: WHZ < −3 and/or bi‐pedal nutritional edema, that is, they were suffering from SAM, and consent obtained from the guardian or parents
	Exclusion criteria: Children were excluded if they had clinically apparent congenital disorders that might affect growth, other acute or chronic diseases requiring continued hospitalisation, active sign of vitamin A deficiency or history of night blindness, active measles or history of measles with in the previous 8 weeks, received high‐dose vitamin A supplementation in the previous 3 months, and lack of a fixed address (to avoid difficulties in tracing for follow‐up examinations)
Interventions	Intervention:
	Group 1 (*n *= 130): High dose Vitamin A, 200,000 IU or 100,000 IU if age < 12 months, on day of admission followed by low dose (5,000 IU) on each subsequent day for 15 days
	Group 2: (*n *= 130) Placebo administered on day of admission followed by low dose vitamin A (5,000 IU) each day for 15 days
	Duration of intervention: 15 days
Outcomes	Primary outcomes: Clinical success within 48 hr of study drug administration
	Secondary outcomes: Adverse event; clinical features of vitamin A toxicity, changes serum retinol and RBP levels, duration of resolution of diarrhoea, ALRI, edema, dermatosis and other illness (if any), changes in weight and length/height, nosocomial morbidities and mortality. Diarrhea was defined as passage of 3 or more watery or semi‐liquid stools in a 24‐hr period
	Timing of outcome assessment: At day 15
Notes	Study start date: June 2005
	Study end date: May 2007
	Funding source: The study was funded by Improved Health for the Poor, Government of the People's Republic of Bangladesh, and supported by ICDDR,B and its donors which provide unrestricted support to the Centre for its operations and research. Current donors providing unrestricted support include Australian Agency for International Development (AusAID), Government of the People's Republic of Bangladesh, Canadian International Development Agency (CIDA), Embassy of the Kingdom of the Netherlands (EKN), Swedish International Development Cooperation Agency (Sida), Swiss Agency for Development and Cooperation (SDC) and Department for International Development (DFID), UK. The study was also supported by Drug International Ltd., Bangladesh
	Conflicts of interest: None declared

Risk of bias table

**Table:** 

Bias	Authors’ judgement	Support for judgement
Random sequence generation (selection bias)	Low risk	Quote: “Computer generated randomisation sequence with block length of 10”
		Comment: Adequately done
Allocation concealment (selection bias)	Low risk	Quote: “…identification codes known to unaffiliated individual, allocation information present in opaque envelope until start of study”
		Comment: Adequately done
Blinding of participants and personnel (performance bias)	Low risk	Quote: “preparations identical in consistency, appearance; identification codes known to unaffiliated individual, allocation information present in opaque envelope until start of study”
		Comment: Adequately done
Blinding of outcome assessment (detection bias)	Low risk	Quote: “preparations identical in consistency, appearance; identification codes known to unaffiliated individual, allocation information present in opaque envelope until start of study”
		Comment: Adequately done
Incomplete outcome data (attrition bias)	Low risk	Comment:
		Group 1: High dose: 27/130
		Group 2: Low dose 26/130
Selective reporting (reporting bias)	Low risk	Comment: Trial registration: ClinicalTrials.gov NCT00388921; outcomes described in methodology section reported in results section
Other bias	Low risk	Comment: No other biases identified

Scherbaum et al. (2015)

**Table:** 

Methods	Design: Quasi‐experimental study (natural experiment)
	Unit: Not applicable
Participants	Location/Setting: Study was carried out in Nias Island, Indonesia
	Sample size: 129 children under 5 years of age
	Dropouts/Withdrawals: 18 children lost to follow‐up
	Sex: Both male and female children included
	Mean age: 34.4 months
	Inclusion criteria: Moderately and mildly wasted children with a weight‐for‐height *z*‐score (WHZ) −3 to <−1.5 *SD*, aged 6 to <60 months, and with no sign of birth defects or disease which would limit the ad libitum food intake, were admitted on the basis of parents’ informed consent and were individually discharged after they reached WHZ >−1.5 *SD*
	Exclusion criteria: Not specified
Interventions	Intervention:
	Group 1: PM‐S: Peanut/milk‐based spreads programme (*n *= 44)
	Peanut/milk‐based spread was given for 4–6 weeks or till recovered
	Group 2: CNL‐B: Cereal/nut/legume‐based biscuits programme (*n *= 47)
	Cereal/nut/legume‐based biscuits was given for 4–6 weeks or till recovered
	Group 3: CNL‐B and intensive nutrition education (INE) (*n *= 38)
	Cereal/nut/legume‐based biscuits + intensive nutrition education was given for 4–6 weeks or till recovered
	Duration of intervention: 4–6 weeks or till recovery
Outcomes	Primary outcomes: Weight gain
	Secondary outcomes: Weight, height, WHZ, recovery, compliance
	Timing of outcome assessment: 4–6 weeks or recovery
Notes	Study start date: October 2007
	Study end date: June 2008
	Funding source: DAAD, DSM Nutritional Product Ltd.‐Basel, Eiselen Foundation Ulm, Neys‐van Hoogstraten Foundation and CWS Indonesia
	Conflicts of interest: None declared

Risk of bias table

**Table:** 

Bias	Authors’ judgement	Support for judgement
Random sequence generation (selection bias)	High risk	Quote: “Due to clear differences in the appearance and consistency of the cereal/nut/legume‐based biscuits (CNL‐B) and peanut/milk‐based spread (PM‐S), a randomised, a double‐blind study design could not be performed”
		Comment: Not done
Allocation concealment (selection bias)	High risk	Quote: “Due to clear differences in the appearance and consistency of the cereal/nut/legume‐based biscuits (CNL‐B) and peanut/milk‐based spread (PM‐S), a randomised, a double‐blind study design could not be performed”
		Comment: Not done
Blinding of participants and personnel (performance bias)	High risk	Quote: “Due to clear differences in the appearance and consistency of the cereal/nut/legume‐based biscuits (CNL‐B) and peanut/milk‐based spread (PM‐S), a randomised, a double‐blind study design could not be performed”
		Comment: Not done
Blinding of outcome assessment (detection bias)	High risk	Quote: “Due to clear differences in the appearance and consistency of the cereal/nut/legume‐based biscuits (CNL‐B) and peanut/milk‐based spread (PM‐S), a randomised, a double‐blind study design could not be performed”
		Comment: Not done
Incomplete outcome data (attrition bias)	High risk	Comment:
		Group PM‐S: 15/44
		Group CNL‐B: 3/47
		Group CNL‐B + INE: 0/38
Selective reporting (reporting bias)	Unclear risk	Comment: No information on trial registration. Outcome outlined in methodology were reported in the results
Other bias	Low risk	Comment: No other biases identified

Shewade et al. (2013)

**Table:** 

Methods	Design: RCT
	Unit: Individual
Participants	Location/Setting: Study was carried out in an urban setting in Chandigarh, India
	Sample size: 26 children aged 6 months to 5 years
	Dropouts/Withdrawals: No loss to follow‐up
	Sex: Both male and female children included
	Mean age: 29 months
	Inclusion criteria: SAM child with all of the following: good appetite, alert and clinically well. Child had to be resident of the area for at least 6 months. SAM children without complications who passed the “appetite test” were accepted for outpatient care
	Exclusion criteria: Complicated SAM— SAM with any one of the following: anorexia, not alert, high fever (>104 F), severe pallor, severe dehydration, lower respiratory tract infection, bipedal edema, visible severe wasting formed the exclusion criteria
Interventions	Intervention:
	Intervention group (*n *= 13)
	RUTF groundnut based prepared by OTS staff was provided on weekly basis for 12 weeks. Diet supplied 200 kcal·kg^−1^·day^−1^
	Control group: (*n *= 13)
	Supplementary nutrition from the Anganwadi as per guidelines for management for malnutrition under the Integrated Child Development Scheme (ICDS)
	Duration of intervention: 12 weeks
Outcomes	Primary outcomes: Weight gain and WHZ
	Secondary outcomes: Average weekly consumption of food intake, HAZ and WAZ
	Timing of outcome assessment: At 12 weeks
Notes	Study start date: July 2011
	Study end date: Not specified
	Funding source: Funding by Indian Association of Preventive and Social Medicine (IAPSM) Ford Foundation Epidemiological Research grant, 2011–2012
	Conflicts of interest: Not specified

Risk of bias table

**Table:** 

Bias	Authors’ judgement	Support for judgement
Random sequence generation (selection bias)	Low risk	Quote: “…computer generated randomised sequence. Independent statistician did block randomisation using block sequence of 4”
		Comment: Adequately done
Allocation concealment (selection bias)	Low risk	Quote: “Allocation concealment was done using numbered, opaque, sealed envelopes”
		Comment: Adequately done
Blinding of participants and personnel (performance bias)	High risk	Quote: “Blinding of study and control group could not be done for obvious reasons”
		Comment: Not done
Blinding of outcome assessment (detection bias)	High risk	Quote: “Blinding of study and control group could not be done for obvious reasons”
		Comment: Not done
Incomplete outcome data (attrition bias)	Low risk	Comment: No loss to follow‐up
Selective reporting (reporting bias)	Low risk	Comment: The trial was registered with Clinical Trials Registry of India (CTRI/2011/12/002259) and pre‐specified outcomes were reported
Other bias	Low risk	Comment: No other biases identified

Sigh et al. (2018)

**Table:** 

Methods	Design: RCT
	Unit: Individual
Participants	Location/Setting: Study was carried out at the National Pediatric Hospital in Phnom Penh, Cambodia
	Sample Size: 121 children 6–59 months of age
	Dropouts/Withdrawals: 49 children lost to follow‐up
	Sex: Both male and female children included
	Mean age: 21.2 (±13.8) months
	Inclusion criteria: All patients diagnosed with SAM without complications aged 6–59 months were eligible for the trial. Patients who have been treated as inpatient prior to enrolment of the trial were also eligible; the diagnostic criteria for SAM to be enrolled in the present trial was set at WHZ ≤ −2.8 and/or MUAC ≤ 115 mm, and/or presence of nutritional edema. The patients must pass an appetite test and the caregivers have to sign an informed consent. Human immunodeficiency virus (HIV) and tuberculosis infections were also included
	Exclusion criteria: Exclusion criteria were uncontrolled or untreatable systemic opportunistic infection, severe cerebral palsy, obvious dysmorphic features, general mental health problems or participation in other clinical trials
Interventions	Intervention:
	Group 1: NumTrey fish‐based RUTF (*n *= 60)
	Two‐week rations of fish‐based wafers‐RUTF (160 and 180 kcal/kg) based on weight were provided at each follow‐up visit. The patients were scheduled to come for follow‐up every 2 weeks and rations were provided for 8 weeks
	Group 2: RUTF‐I (BP100) (*n *= 61)
	Two‐week rations of a standard product BP‐100™ (160 and 180 kcal/kg), produced by the company Compact in Norway were provided. Patients were scheduled to come for follow‐up every two weeks and rations were provided for 8 weeks
	Duration of intervention: 8 weeks
Outcomes	Primary outcomes: Weight gain
	Secondary outcomes: Changes in total weight (g), height (cm), MUAC (mm), WHZ, weight‐for‐age *z*‐score (WAZ) and height‐for‐age *z*‐score (HAZ)
	Timing of outcome assessment: At 8 weeks
Notes	Study start date: September 2015
	Study end date: January 2017
	Funding source: This research was funded by UNICEF's national committees (Australia, Korea, and Hong Kong), Institut de Recherché pour le Développement, University of Copenhagen, Denmark and Neys‐van Hoogstraten Foundation, The Netherlands (grant number: CA271)
	Conflicts of interest: None declared

Risk of bias table

**Table:** 

Bias	Authors’ judgement	Support for judgement
Random sequence generation (selection bias)	Low risk	Quote: “A computer‐generated randomisation list in blocks of four patients based on the product codes and patient ID number was made prior to the start of the trial”
		Comment: Adequately done
Allocation concealment (selection bias)	Low risk	Quote: “The list was provided in a closed envelope to the project manager, who enrolled participants and assigned the intervention to the participants based on the list”
		Comment: Adequately done
Blinding of participants and personnel (performance bias)	High risk	Quote: “The RUTFs and the packaging were visibly different from each other, therefore, the blinding of hospital staff, participants, and the project staff responsible for outcome measures was not possible”
		Comment: Not done
Blinding of outcome assessment (detection bias)	Low risk	Quote: “The codes were prepared to blind the researcher (S.S) who was responsible for the trial. The researcher (S.S) supervised the project staff during the trial, cleaned the data and conducted the data analysis after the trial. The primary analysis of data had been completed before the code was provided to her”
		Comment: Adequately done
Incomplete outcome data (attrition bias)	High risk	Comment:
		BP 100: 23/61
		NumTrey: 26/60
Selective reporting (reporting bias)	Low risk	Comment: The trial is registered at ClinicalTrials.gov (Trial name: “Comparison of a Locally Produced RUTF with a Commercial RUTF in the Treatment of SAM (FLNS_SAM)”, trial registration; NCT02907424) and pre‐specified outcomes were reported
Other bias	Low risk	Comment: No other biases identified

Singh et al. (2010)

**Table:** 

Methods	Design: RCT
	Unit: Individual
Participants	Location/Setting: Study was carried out in a rural setting in Vellore, India
	Sample size: 118 children aged 18–60 months
	Dropouts/Withdrawals: 22 lost to follow‐up
	Sex: Both make and female children were included
	Mean age: 3.54 months
	Inclusion criteria: Children aged 18–60 months, −2 *SD* weight‐for‐age and below but not requiring hospitalisation for malnutrition, were considered eligible
	Exclusion criteria: Children younger than 18 months were excluded as several of them were receiving a predominantly milk diet, as chosen by their parents
Interventions	Intervention:
	Group 1: RUTF (*n *= 61)
	Daily administration of 50 g of RUTF composed of ground roasted peanut powder, milk powder, and sugar in a ratio of 30:28:25 (g), along with 15 g of gingili oil. The RUTF was prepared at a local bakery under supervision and a weekly bag of 250 g of supplementation was provided
	Group 2: HCCM (*n *= 57)
	Mothers were taught to prepare High Caloric Cereal Milk (HCCM) supplement. HCCM consisted of 100 ml milk fortified with 15 g flour of mother's choice, 5 ml oil and 2 teaspoons of sugar, cooked to a porridge‐like consistency. Two servings of HCCM made with 100 ml of milk each were given at home
	Duration of intervention: 3 months
Outcomes	Primary outcomes: Recovery
	Secondary outcomes: Changes in the vitamin B12, plasma Zinc, serum albumin levels and iron status of the children
	Timing of outcome assessment: At 30, 60 and 90 days
Notes	Study start date: Jan 2008
	Study end date: May 2008
	Funding source: The Fogarty International Clinical Research Scholars Program and the Department of Gastrointestinal Sciences, Christian Medical College, Vellore
	Conflicts of interest: None declared

Risk of bias table

**Table:** 

Bias	Authors’ judgement	Support for judgement
Random sequence generation (selection bias)	Low risk	Quote: “Block randomisation was done in blocks of ten using a computer‐generated sequence, generated by the statistician”
		Comment: Adequately done
Allocation concealment (selection bias)	High risk	Quote: “The children were allocated to either group by one of the investigators”
		Comment: Probably not done
Blinding of participants and personnel (performance bias)	High risk	Quote: “The study is not ideal in that it was not blinded, but blinding would have been difficult for two very different but acceptable interventions”
		Comment: Not done
Blinding of outcome assessment (detection bias)	High risk	Quote: “The study is not ideal in that it was not blinded, but blinding would have been difficult for two very different but acceptable interventions”
		Comment: Not done
Incomplete outcome data (attrition bias)	Low risk	Comment:
		RUTF: 10/61
		HCCM: 12/57
Selective reporting (reporting bias)	Low risk	Comment: Registered at the Clinical Trials Registry of India; Registration number: CTRI/2009/09/000007 and pre‐specified outcomes reported
Other bias	Low risk	Comment: No other biases identified

Stobaugh et al. (2016)

**Table:** 

Methods	Design: RCT
	Unit: Individual
Participants	Location/Setting: Study was carried out in a rural setting in South Malawi/Mozambique border residents
	Sample size: 2,259 children aged 6–59 months of age
	Dropouts/Withdrawals: 29 lost to follow‐up
	Sex: Both male and female children were included
	Mean age: 16.5 months
	Inclusion criteria: Children aged 6–59 months with MAM, as defined by a mid‐upper arm circumference (MUAC) of 11.5–12.4 cm without bipedal edema (20, 21), were recruited
	Exclusion criteria: Children with chronic illnesses (not including HIV or tuberculosis) or a known allergy to milk, soy or peanuts; those who had received treatment for acute malnutrition in the previous 3 months; and those who were not permanent residents of the vicinity near the clinic site were excluded
Interventions	Intervention:
	Group 1: Whey–protein RUSF (*n *= 1,144)
	A dairy‐based, whey protein, whey permeate concentrate (75 kcal·kg^−1^·day^−1^) was given every 2 weeks for 12 weeks
	Group 2: soy‐flour RUSF (*n *= 2,086)
	Extruded soy flour (75 kcal·kg^−1^·day^−1^) was given every 2 weeks for 12 weeks
	Nutrition counselling and instruction for feeding were given to both groups
	Duration of intervention: 12 weeks
Outcomes	Primary outcomes: Recovery
	Secondary outcomes: Changes in MUAC, weight, and length; time to recovery; and any adverse events
	Timing of outcome assessment: At 12 weeks
Notes	Study start date: February 2013
	Study end date: November 2014
	Funding source: Funding for this project was provided by the Danish Dairy Research Foundation, Arla Foods Ingredients Group P/S, and the U.S. Dairy Export Council. IT was supported by the Children's Discovery Institute of Washington University in St. Louis and St. Louis Children's Hospital
	Conflicts of interest: None of the authors reported a conflict of interest related to the study.

Risk of bias table

**Table:** 

Bias	Authors’ judgement	Support for judgement
Random sequence generation (selection bias)	Low risk	Quote: “Random allocation was performed by caregivers drawing opaque envelopes that contained 1 of 2 coded papers corresponding to either whey RUSF or soy RUSF. This code was accessible only to the food distribution personnel, who did not assess participant outcomes, determine eligibility, or analyse data”
		Comment: Adequately done
Allocation concealment (selection bias)	Low risk	Quote: “Random allocation was performed by caregivers drawing opaque envelopes that contained 1 of 2 coded papers corresponding to either whey RUSF or soy RUSF. This code was accessible only to the food distribution personnel, who did not assess participant outcomes, determine eligibility, or analyse data”
		Comment: Adequately done
Blinding of participants and personnel (performance bias)	Low risk	Quote: “The 2 RUSF formulations had similar colour, taste, smell, and packaging”
		Comment: Adequately done
Blinding of outcome assessment (detection bias)	Low risk	Quote: “This code was accessible only to the food distribution personnel, who did not assess participant outcomes, determine eligibility, or analyse data”
		Comment: Adequately done
Incomplete outcome data (attrition bias)	Low risk	Comment:
		Soy RUSF: 17/1103
		Whey RUSF: 12/1156
Selective reporting (reporting bias)	Low risk	Comment: This study was registered at clinicaltrials.gov as NCT01790048 and pre‐specified outcomes were reported
Other bias	Low risk	Comment: No other biases identified

Thakur et al. (2013)

**Table:** 

Methods	Design: Quasi‐experimental study (allocation is not truly random)
	Unit: Individual
Participants	Location/Setting: Study was carried out in an urban setting in Maharashtra, India
	Sample size: 98 children ages 6–60 months
	Dropouts/Withdrawals: 6 children lost to follow‐up
	Sex: Both male and female children were included
	Mean age: Not specified
	Inclusion criteria: All patients aged 6–60 months, diagnosed as severe acute malnutrition hospitalised in the institution during the study period (1 October 2009 to 30 May 2010) were included in study
	Exclusion criteria: Patients were excluded from study if they refused to get hospitalised, refused for consent, left against medical advice before discharge or died during stabilization phase. All children below age of 6 months with severe acute malnutrition were considered complicated and hospitalised, but they were excluded from study
Interventions	Intervention:
	Group 1: L‐RUTF(*n *= 50)
	Groundnut, milk powder, vegetable oil was given as 4 meals/day (12 g·kg^−1^·day^−1^) along with 4 meals from family pot. Frequency of intervention was every 2 weeks
	Group 2: F100‐L (*n *= 54)
	F100 locally produced was given as 60 ml·kg^−1^·day^−1^ in 4 quarters + 4 meals from family food (total 120 kcal·kg^−1^·day^−1^). Diet was supplied every 2 weeks
	Duration of intervention: 2 weeks
Outcomes	Primary outcomes: Weight gain
	Secondary outcomes: Duration of hospital stay
	Timing of outcome assessment: At 2 weeks
Notes	Study start date: October 2009
	Study end date: May 2010
	Funding Source: None
	Conflicts of interest: None declared

Risk of bias table

**Table:** 

Bias	Authors’ judgement	Support for judgement
Random sequence generation (selection bias)	High risk	Quote: “The study was non‐randomised controlled trial. Patients were divided into two groups depending on the dates of hospitalisation”
		Comment: Not adequate
Allocation concealment (selection bias)	High risk	Quote: “The study was non‐randomised controlled trial. Patients were divided into two groups depending on the dates of hospitalisation”
		Comment: Not adequate
Blinding of participants and personnel (performance bias)	High risk	Quote: “There was a practical difficulty in blinding because of different appearance of the two therapeutic regimens”
		Comment: Not done
Blinding of outcome assessment (detection bias)	High risk	Quote: “There was a practical difficulty in blinding because of different appearance of the two therapeutic regimens”
		Comment: Not done
Incomplete outcome data (attrition bias)	Low risk	Comment:
		F100: 5/54
		LRUTF: 1/50
Selective reporting (reporting bias)	Unclear risk	Comment: Trial registration information not provided. Outcomes in the methods section were reported in the results section
Other bias	Low risk	Comment: No other biases identified

Thakwalakwa et al. (2010)

**Table:** 

Methods	Design: RCT
	Unit: Individual
Participants	Location/Setting: Study was carried out in a rural setting of Lungwena, Mangochi District of Malawi
	Sample size: 189 children aged 6–15 months
	Dropouts/Withdrawals: 4 children lost to follow‐up
	Sex: Both male and female children were included
	Mean age: 11.3 months
	Inclusion criteria: Moderately underweight infants and children who met the following inclusion criteria: a signed, informed consent from at least 1 guardian, aged between 6 and 15 months, WAZ < −2 based on the National Centre for Health Statistics/Centers for Disease Control and Prevention (NCHS/CDC) growth reference, availability during the study period, and permanent residence in the study catchment area were included
	Exclusion criteria: Exclusion criteria included WLZ < −3 or presence of edema, history of peanut allergy, history of any serious allergic reaction to any substance requiring emergency medical care, history of anaphylaxis, severe illness warranting hospital referral, and concurrent participation in another clinical trial with nutrition intervention for the child
Interventions	Intervention:
	Group 1: CSB (*n *= 67)
	Corn–soy blend given weekly for 12 weeks
	Group 2: LNS (*n *= 66)
	Peanut paste, dry skim milk, veg oil, sugar, min–vit mix given weekly for 12 weeks
	Group 3: No supplement‐NS (*n *= 59)
	Infants breast fed only
	Duration of intervention: 12 weeks
Outcomes	Primary outcomes: Weight change
	Secondary outcomes: Mean changes in length (mm), haemoglobin (Hb) concentration (g/L), weight‐for‐length *z*‐score (WLZ), length‐for‐age *z*‐score (LAZ), mid‐upper arm circumference (MUAC), head circumference, and incidence of adverse events (AE) or serious AE (SAE)
	Timing of outcome assessment: At 12 weeks
Notes	Study start date: December 2007
	Study end date: February 2007
	Funding source: Supported by the Academy of Finland (grant no. 109796)
	Conflicts of interest: One author (A. B.) worked as a consultant to Nutriset until December 2003. Others had no conflicts of interest

Risk of bias table

**Table:** 

Bias	Authors’ judgement	Support for judgement
Random sequence generation (selection bias)	Low risk	Quote: “The envelopes were marked with the trial code and stored in a locked cabinet until use. A consenting guardian of an eligible individual was asked to choose 1 randomisation envelope from the remaining unused envelopes at a time. The identification number found in the envelope was recorded in a logbook and on the participant's picture identification card. The identification card was given to the guardian and used for participant identification during the trial”
		Comment: Not adequately done
Allocation concealment (selection bias)	Low risk	Quote: “The allocation for each consecutive consented participant was sealed in an individual opaque randomisation envelope”
		Comment: Adequately done
Blinding of participants and personnel (performance bias)	High risk	Quote: “This was a single‐centre, randomised, controlled, investigator‐blinded clinical trial”
		Comment: Not done
Blinding of outcome assessment (detection bias)	Low risk	Quote: “A trial physician (J. P.) who was unaware of the participants’ group allocations reviewed the data on suspected AE and determined and classified the severity of AE and the likelihood of their association to the trial interventions”
		Comment: Adequately done
Incomplete outcome data (attrition bias)	Low risk	Comment:
		CSB: 1/67
		LNS: 1/66
		No food group: 2/59
Selective reporting (reporting bias)	Low risk	Comment: This trial was registered at clinicaltrials.gov as NCT00420368 and all pre‐specified outcomes were reported
Other bias	Low risk	Comment: No other biases found

Vanelli et al. (2014)

**Table:** 

Methods	Design: RCT
	Unit: Individual
Participants	Location/Setting: Study was carried out in Makeni, Northern region, Sierra Leonne
	Sample size: 332 children.aged 6–60 months
	Dropouts/Withdrawals: 45 children loss to follow‐up
	Sex: Both male and female children were included
	Mean age: 14 (±6.3) months
	Inclusion criteria: Children aged 6–60 months with moderate malnutrition degree were considered eligible for this study
	Exclusion criteria: Children affected of an acquired chronic disease were excluded from the study
Interventions	Intervention:
	Group 1: (*n *= 177)
	Feeding Program Supplementations (FPS) only. FPS contained corn flour, palm oil, dried fish, milk powder (UN Food Prog Sup) given weekly for 12 weeks
	Group 2: (*n *= 159)
	FPS + Parma‐pap; Group 2 children were given a number of 100‐g servings of “Parma pap” equal to the weekly requirement containing peanut, palm oil, milk, mineral–vit mix given weekly for 12 weeks
	Duration of intervention: 12 weeks
Outcomes	Primary outcomes: Weight and length
	Secondary outcomes: Weight for‐height *z*‐score (WHZ)
	Timing of outcome assessment: After 12 weeks
Notes	Study start date: July 2009
	Study end date: July 2012
	Funding source: The study has been supported by a grant from the “Fondazione Cassa di Risparmio di Parma”, Parma, Italy
	Conflicts of interest: None declared

Risk of bias table

**Table:** 

Bias	Authors’ judgement	Support for judgement
Random sequence generation (selection bias)	Unclear risk	Quote: “At the time of being admitted to the study, children were randomly distributed into two groups”
		Comment: Insufficient information
Allocation concealment (selection bias)	Unclear risk	Quote: “At the time of being admitted to the study, children were randomly distributed into two groups”
		Comment: Insufficient information
Blinding of participants and personnel (performance bias)	High risk	Comment: Probably not done
Blinding of outcome assessment (detection bias)	High risk	Comment: Probably not done
Incomplete outcome data (attrition bias)	Low risk	Comment:
		Group 1: 35/177
		Group 2: 10/159
Selective reporting (reporting bias)	Unclear risk	Comment: Trial registration not specified. Outcomes listed in the methodology section were discussed in the results section
Other bias	Low risk	Comment: No other biases identified

Versloot et al. (2017)

**Table:** 

Methods	Design: RCT
	Unit: Individual
Participants	Location/Setting: Study was carried out in Blantyre, Malawi
	Sample size: 74 children aged 6–60 months
	Dropouts/Withdrawals: No loss to follow‐up
	Sex: Both male and female children were included
	Mean age: 23.7 months
	Inclusion criteria: Children aged 6–60 months, diagnosed with SAM and already admitted to the nutritional rehabilitation unit (NRU) but still in the stabilization phase were included in the TranSAM trial after written informed consent. Both HIV positive and negative children, diagnosed by rapid antibody testing upon admission were included
	Exclusion criteria: Exclusion criteria were admission to the nutritional rehabilitation unit within the past year, severe haemodynamic instability, hematocrit level ≤ 15% and severe neurological symptoms
Interventions	Intervention:
	Group 1: RUTF‐F75 (*n *= 26)
	Low protein milk‐based formula diet given daily for 7 days (energy intake of 135 kcal·kg^−1^·day^−1^)
	Group 2: F100 (*n *= 25)
	F100 milk diet given daily for 7 days (energy intake of 135 kcal·kg^−1^·day^−1^)
	Group 3: RUTF(*n *= 23)
	Ready‐use‐therapeutic food diet given daily for 7 days (energy intake of 135 kcal·kg^−1^·day^−1^)
	Duration of intervention: Till discharge
Outcomes	Primary outcomes: Fecal pH
	Secondary outcomes: Duration of stay from the first day of the transition to discharge from the ward, days with diarrhoea, duration of edema, weight at discharge, hypo‐ and hypernatraemia, reversion to F75 diet and mortality
	Timing of outcome assessment: Fecal pH was assessed 3 days after the start of the transition phase
Notes	Study start date: January 2013
	Study end date: July 2013
	Funding source: No funding received
	Conflicts of interest: None declared

Risk of bias table

**Table:** 

Bias	Authors’ judgement	Support for judgement
Random sequence generation (selection bias)	Low risk	Quote: “The allocation sequence was computer generated by an independent collaborator”
		Comment: Adequately done
Allocation concealment (selection bias)	Low risk	Quote: “Allocation concealment was achieved by using sealed, sequentially numbered opaque envelopes containing a label for 1 of the 3 transition phase feeds”
		Comment: Adequately done
Blinding of participants and personnel (performance bias)	Low risk	Quote: “The caregivers were given colour‐coded milk cards corresponding to a specific diet”
		Comment: Adequately done
Blinding of outcome assessment (detection bias)	Unclear risk	Quote: “The caregivers were given colour‐coded milk cards corresponding to a specific diet. Our research team was trained to distribute the correct milk formulae to participants”
		Comment: Insufficient information
Incomplete outcome data (attrition bias)	Low risk	Comment: No loss to follow‐up
Selective reporting (reporting bias)	Low risk	Comment; The trial was registered as ISRCTN13916953 and outcomes described in method section were reported in the results section
Other bias	Low risk	Comment: No other biases identified

Wilford et al. (2011)

**Table:** 

Methods	Design: Cost effectiveness analysis using the decision tree model
	Unit: Not applicable
Participants	Location/Setting: Study was carried out in District Dowa, Central Malawi
	Sample size: Not applicable
	Dropouts/Withdrawals: Not applicable
	Sex: Not applicable
	Mean age: Not applicable
	Inclusion criteria: Not applicable
	Exclusion criteria: Not applicable
Interventions	The study assessed the cost‐effectiveness of community‐based management of acute malnutrition (CMAM) to prevent deaths due to severe acute malnutrition among children under‐five. The analysis used a decision tree model to compare the costs and effects of two options to treat severe acute malnutrition: existing health services with CMAM versus existing health services without CMAM
Outcomes	Primary outcomes: Cost and cost‐effectiveness
Notes	Study start date: January 2007
	Study end date: December 2007
	Funding source: Concern Worldwide
	Conflicts of interest: Not specified

Risk of bias table

**Table:** 

Bias	Authors’ judgement	Support for judgement
Random sequence generation (selection bias)	Unclear risk	
Allocation concealment (selection bias)	Unclear risk	
Blinding of participants and personnel (performance bias)	Unclear risk	
Blinding of outcome assessment (detection bias)	Unclear risk	
Incomplete outcome data (attrition bias)	Unclear risk	
Selective reporting (reporting bias)	Unclear risk	
Other bias	Unclear risk	

**Characteristics of excluded studies**

**Table:** 

	Reason for exclusion
Agha 2004	This study did not have an appropriate control group.
Aguayo et al. 2018	The study design was not appropriate.
Ahmed et al. 1999	The study design was not appropriate.
Ashworth et al. 2004	The study design was not appropriate.
Bachou et al., 2008	The study design was not appropriate.
Badaloo et al. 1999	This study did not assess the intervention of interest; study compared high protein formula with low protein formula.
Baker et al. 1978	The study did not assess the intervention of interest; study compared milk diet with soy‐maize‐porridge diet.
Bhandari et al. 2001	The study did not assess the intervention of interest; study compared food supplementation with counselling with nutritional counselling alone.
Burza et al. 2016	The study design was not appropriate.
Donnen et al. 2007	This study included children up to 14 years of age.
Dubray et al. 2008	This study compared two different antibiotics (ceftriaxone vs amoxicillin) in children with SAM and did not have an appropriate control group (no antibiotic/placebo).
Gaboulaud et al. 2006	The study does not have an appropriate control group.
Javan et al., 2017	This study was conducted in Upper Middle Income Country.
Linneman et al. 2007	This study did not have an appropriate control group.
Nagar et al. 2016	This study did not have an appropriate control group.
Roy et al. 2005	The study did not assess the intervention of interest; study compared supplementary feeding with education to feeding alone.
Simpore et al. 2006	This study did not have an appropriate control group.
Zongo et al., 2013	The study did not assess the intervention of interest; the study compared Moringa leaf in addition to the usual porridge diet.

*Footnotes*

**Characteristics of studies awaiting classification**

*Footnotes*

**Characteristics of ongoing studies**

*Footnotes*

**Summary of findings tables**

**1. Summary of findings**

**Table:** 

Community‐based strategies compared with standard care for moderate and severe acute malnutrition						
Patient or population: Children under 5 years of age with moderate and severe acute malnutrition						
Settings: Communities and nutrition rehabilitation centres in low‐ middle‐ income countries						
Intervention: Integrated community based management						
Comparison: Standard management						
Outcomes	Illustrative comparative risks^a^ (95% CI)		Relative effect (95% CI)	No. of participants (studies)	Quality of the evidence (GRADE)	Comments
	Assumed risk	Corresponding risk				
	Standard management	Integrated management				
Recovery	795 per 1,000	827 per 1,000	RR: 1.04 (1.00 to 1.09)	1,957 participants (one study)	⊕⊕⊕⊝	
(Assessed at 12 weeks)					
					Moderate^b^
Weight gain	The mean weight gain ranged in control group was 3.8 g·kg^−1^·day^−1^	The mean weight gain in the intervention groups was 0.8 g·kg^−1^·day^−1^ lower (0.82 g·kg^−1^·day^−1^ lower to 0.78 g·kg^−1^·day^−1^ lower) than the control group		1,957 participants (one study)	⊕⊕⊕⊝	
			Moderate^b^			
(g·kg^−1^·day^−1^, Assessed for first 4 weeks)						
Mortality	40 per 1,000	38 per 1,000	RR: 0.93 (0.60 to 1.45)	1,957 participants (one study)	⊕⊕⊕⊝	
					Moderate^b^
(Assessed at 12 weeks)					

*Note*: GRADE Working Group grades of evidence.

High quality: Further research is very unlikely to change our confidence in the estimate of effect.

Moderate quality: Further research is likely to have an important impact on our confidence in the estimate of effect and may change the estimate.

Low quality: Further research is very likely to have an important impact on our confidence in the estimate of effect and is likely to change the estimate.

Very low quality: We are very uncertain about the estimate.

Abbreviations: CI, confidence interval; RR, risk ratio.

^a^ The basis for the assumed risk (e.g., the median control group risk across studies) is provided in footnotes. The corresponding risk (and its 95% CI) is based on the assumed risk in the comparison group and the relative effect of the intervention (and its 95% CI).

^b^ Downgraded due to study limitations.

**2. Summary of findings**

**Table:** 

Inpatient management compared with outpatient/community‐based management for severe acute malnutrition (SAM)						
Patient or population: Children under 5 years of age with SAM						
Settings: Hospitals, nutrition rehabilitation centres and communities in low‐ middle‐ income countries						
Intervention: Facility or in‐patient management						
Comparison: Community or out‐patient management						
Outcomes	Illustrative comparative risks^a^ (95% CI)		Relative effect (95% CI)	No. of participants (studies)	Quality of the evidence (GRADE)	Comments
	Assumed risk	Corresponding risk				
	Outpatient/Community management	Inpatient/Facility management				
Recovery	926 per 1,000	527 per 1,000	RR: 0.57 (0.53 to 0.62)	1,000 participants (one study)	⊕⊕⊕⊝	
(Assessed at 4‐6 weeks)					
					Moderate^b^	
Mortality	48 per 1,000	167 per 1,000	RR: 2.32 (0.42 to 12.83)	1,473 participants (three studies)	⊕⊕⊝⊝	
(Assessed at the end of the study; 4–6 weeks)					
					Low^b^, ^c^	

*Note*: GRADE Working Group grades of evidence.

High quality: Further research is very unlikely to change our confidence in the estimate of effect.

Moderate quality: Further research is likely to have an important impact on our confidence in the estimate of effect and may change the estimate.

Low quality: Further research is very likely to have an important impact on our confidence in the estimate of effect and is likely to change the estimate.

Very low quality: We are very uncertain about the estimate.

Abbreviations: CI, confidence interval; RR, risk ratio; SAM, severe acute malnutrition.

^a^ The basis for the assumed risk (e.g., the median control group risk across studies) is provided in footnotes. The corresponding risk (and its 95% CI) is based on the assumed risk in the comparison group and the relative effect of the intervention (and its 95% CI).

^b^ Downgraded due to study limitations.

^c^ Downgraded due to high heterogeneity.

**3. Summary of findings**

**Table:** 

Inpatient RUTF compared with inpatient F100 for severe acute malnutrition (SAM)						
Patient or population: Children under 5 years of age with SAM						
Settings: Hospitals and nutrition rehabilitation centres in low‐ and middle‐income countries						
Intervention: RUTF						
Comparison: F100						
Outcomes	Illustrative comparative risks^a^ (95% CI)		Relative effect (95% CI)	No. of participants (studies)	Quality of the evidence (GRADE)	Comments
	Assumed risk	Corresponding risk				
	F100	RUTF				
Weight gain	The mean weight gain ranged across control groups from 6.5 to 9.59 g·kg^−1^·day^−1^	The mean weight gain in the intervention group was 2 g·kg^−1^·day^−1^ higher in the RUTF group (0.23 g·kg^−1^·day^−1^ lower to 4.23 g·kg^−1^·day^−1^ higher)		266 participants (three studies)	⊕⊝⊝⊝	
			Very low^b^, ^c^, ^d^	
(g·kg^−1^·day^−1^; weight gain assessed at the time of discharge; till recovery)					
Mortality	70 per 1,000	84 per 1,000	RR: 1.20 (0.34 to 4.22)	168 participants (two studies)	⊕⊕⊝⊝	
(Assessed at the end of the study)					
					Low^b^, ^d^	

*Note*: GRADE Working Group grades of evidence.

High quality: Further research is very unlikely to change our confidence in the estimate of effect.

Moderate quality: Further research is likely to have an important impact on our confidence in the estimate of effect and may change the estimate.

Low quality: Further research is very likely to have an important impact on our confidence in the estimate of effect and is likely to change the estimate.

Very low quality: We are very uncertain about the estimate.

Abbreviations: CI, confidence interval; RR, risk ratio; RUTF, ready‐to‐use therapeutic food; SAM, severe acute malnutrition.

^a^ The basis for the **assumed risk** (e.g., the median control group risk across studies) is provided in footnotes. The **corresponding risk** (and its 95% CI) is based on the assumed risk in the comparison group and the **relative effect** of the intervention (and its 95% CI).

^b^ Downgraded due to study limitations.

^c^ Downgraded due to high heterogeneity.

^d^ Downgraded due to imprecision and small sample size.

**4. Summary of findings**

**Table:** 

Community‐based management with standard RUTF compared with other food for severe acute malnutrition (SAM)							
Patient or population: Children under 5 years of age with SAM							
Settings: Communities and nutrition rehabilitation centres in low‐ and middle‐income settings							
Intervention: Community‐based management with RUTF							
Comparison: Community‐based management with other food (including non‐standard RUTF; home prepared food; F100)							
Outcomes		Illustrative comparative risks^a^ (95% CI)		Relative effect (95% CI)	No of Participants (studies)	Quality of the evidence (GRADE)	Comments
		Assumed risk	Corresponding risk				
		Other food	Standard RUTF				
Recovery	Standard dairy/peanut butter RUTF versus non/reduced dairy/peanut butter	733 per 1,000	752 per 1,000	RR: 1.03 (0.99 to 1.08)	5,743 participants (5 studies)	⊕⊕⊕⊝	
						Moderate^b^
(Assessed at either 8, 12, 14 or 16 weeks or at discharge)							
	Standard RUTF versus energy dense home‐prepared food	571 per 1,000	622 per 1,000	RR: 1.14 (0.95 to 1.36)	959 participants (4 studies)	⊕⊕⊝⊝	
						Low^b^, ^c^	
	Standard RUTF versus high‐oleic RUTF	676 per 1,000	714 per 1,000	RR: 1.06 (0.85 to 1.31)	141 participants (1 study)	⊕⊕⊕⊝	
						Moderate^d^	
Weight gain	Standard dairy/peanut butter RUTF versus non/reduced dairy/peanut butter	The mean weight gain ranged across control group from 1.08 to 2.2 g·kg^−1^·day^−1^	The mean weight gain in the intervention groups was 0.5 g·kg^−1^·day^−1^ higher than the control group (0.02 g·kg^−1^·day^−1^ higher to 0.99 g·kg^−1^·day^−1^ higher)		3,069 participants (3 studies)	⊕⊕⊝⊝	
(g·kg^−1^·day^−1^; Assessed at either 8, 12, 14 or 16 weeks or at discharge)				Low^b^, ^c^			
	RUTF versus F100	The mean weight gain in the control group was 10.1 g·kg^−1^·day^−1^	The mean weight gain in the intervention group was 5.5 g·kg^−1^·day^−1^ higher than the control group (2.92 g·kg^−1^·day^−1^ higher to 8.08 g·kg^−1^·day^−1^ higher)		70 participants (1 study)	⊕⊕⊝⊝	
				Low^d^, ^b^			
	Standard RUTF versus energy dense home‐prepared food	The mean weight gain ranged across the control from 2.64 to 5.6 g·kg^−1^·day^−1^	The mean weight gain in the intervention group was 0.35 g·kg^−1^·day^−1^ lower than the control group (1.52 g·kg^−1^·day^−1^ lower to 0.82 g·kg^−1^·day^−1^ higher)		1,925 participants (3 studies)	⊕⊕⊝⊝	
				Low^b^, ^c^			
		Standard RUTF versus high‐oleic RUTF	The mean weight gain in the control group was 2.8 g·kg^−1^·day^−1^	The mean weight gain in the intervention group was 0.82 g·kg^−1^·day^−1^ lower than the control group (1.74 g·kg^−1^·day^−1^ lower to 0.14 g·kg^−1^·day^−1^ higher)	141 participants (1 study)	⊕⊕⊕⊝	
Moderate^d^	
Mortaity	Standard dairy/peanut butter RUTF versus non/reduced dairy/peanut butter	59 per 1,000	59 per 1,000	RR: 0.90 (0.72 to 1.12)	5,743 participants (5 studies)	⊕⊕⊕⊝	
						Moderate^b^	
(Assessed at either 8, 12, 14 or 16 weeks or at discharge)							
	Standard RUTF versus energy dense home‐prepared food	23 per 1,000	25 per 1,000	RR: 1.87 (0.95 to 3.70)	1,743 participants (2 studies)	⊕⊕⊕⊝	
						Moderate^b^	
	Standard RUTF versus high‐oleic RUTF	14 per 1,000	71 per 1,000	RR: 5.07 (0.61 to 42.31)	141 participants (1 study)	⊕⊕⊕⊝	
						Low^d^	
	Standard RUTF versus *n*3 PUFA RUTF	150 per 1,000	50 per 1,000	RR: 0.33 (0.04 to 2.94)	40 participants (1 study)	⊕⊕⊝⊝	
						Low^b^, ^d^	

*Note*: GRADE Working Group grades of evidence.

High quality: Further research is very unlikely to change our confidence in the estimate of effect.

Moderate quality: Further research is likely to have an important impact on our confidence in the estimate of effect and may change the estimate.

Low quality: Further research is very likely to have an important impact on our confidence in the estimate of effect and is likely to change the estimate.

Very low quality: We are very uncertain about the estimate.

Abbreviations: CI, confidence interval; RR, risk ratio; RUTF, ready‐to‐use therapeutic food; SAM, severe acute malnutrition.

^a^ The basis for the assumed risk (e.g., the median control group risk across studies) is provided in footnotes. The corresponding risk (and its 95% CI) is based on the assumed risk in the comparison group and the relative effect of the intervention (and its 95% CI).

^b^ Downgraded due to study limitations.

^c^ Downgarded due to high heterogeneity.

^d^ Downgraded due to imprecision and small sample size.

**5. Summary of findings**

**Table:** 

RUSF compared with other foods for moderate acute malnutrition (MAM)							
Patient or population: Children under 5 years of age with MAM							
Settings: Communities and nutrition rehabilitation centres							
Intervention: RUSF							
Comparison: Other foods including local/home made food, whey RUSF, CSB, supplementary food							
Outcomes		Illustrative comparative risks^a^ (95% CI)		Relative effect (95% CI)	No. of participants (studies)	Quality of the evidence (GRADE)	Comments
		Assumed risk	Corresponding risk				
		Other foods	RUSF				
Recovery	RUSF versus local/home made food	676 per 1,000	735 per 1,000	RR: 0.92 (0.64 to 1.33)	435 participants (3 studies)	⊕⊕⊝⊝	
(Assessed at 6–12 weeks)						Low^b^, ^c^	
	Standard RUSF versus whey RUSF	839 per 1,000	804 per 1,000	RR: 0.96 (0.92 to 1.00)	2,230 participants (1 study)	⊕⊕⊕⊕	
						High	
	RUSF versus CSB	741 per 1,000	797 per 1,000	RR: 1.07 (1.02 to 1.13)	5,744 participants (6 studies)	⊕⊕⊝⊝	
						Low^b^, ^c^	
Weight gain	RUSF versus local/home made food	The mean weight gain in control group was 1.76 g·kg^−1^·day^−1^	The mean weight gain in the intervention groups was 0.27 g·kg^−1^·day^−1^ lower (0.74 g·kg^−1^·day^−1^ lower to 0.2 g·kg^−1^·day^−1^ higher) than the control group.		73 participants (1 study)	⊕⊕⊝⊝	
(g·kg^−1^·day^−1^; Assessed at 6–12 weeks)				Low^b^, ^d^			
	Standard RUSF versus whey RUSF	The mean weight gain in control group was 2.95 g·kg^−1^·day^−1^	The mean weight gain in the intervention groups was 0.08 g·kg^−1^·day^−1^ lower (0.16 g·kg^−1^·day^−1^ lower to 0.01 g·kg^−1^·day^−1^ higher) than the control group.		2,230 participants (1 study)	⊕⊕⊕⊕	
				High			
	RUSF versus CSB	The mean weight gain in control group was 2.36 g·kg^−1^·day^−1^	The mean weight gain in the intervention groups was 0.21 g·kg^−1^·day^−1^ higher (0.06 g·kg^−1^·day^−1^ higher to 0.37 g·kg^−1^·day^−1^ higher) than the control group.		4,354 participants (5 studies)	⊕⊕⊝⊝	
				Low^b^, ^c^			
Mortality	Standard RUSF versus whey RUSF	1 per 1,000	3 per 1,000	RR: 2.11 (0.39 to 11.48)	2,230 participants (1 study)	⊕⊕⊕⊕	
						High
(Assessed at 6–12 weeks)							
	RUSF versus CSB	7 per 1,000	7 per 1,000	RR: 0.92 (0.51 to 1.67)	5,744 participants (6 studies)	⊕⊕⊕⊝	
						Moderate^b^	
	RUSF versus food supplement	11 per 1,000	6 per 1,000	RR: 0.56 (0.05 to 6.08)	336 participants (1 study)	⊕⊕⊝⊝	
						Low^b^, ^d^	

*Note*: GRADE Working Group grades of evidence.

High quality: Further research is very unlikely to change our confidence in the estimate of effect.

Moderate quality: Further research is likely to have an important impact on our confidence in the estimate of effect and may change the estimate.

Low quality: Further research is very likely to have an important impact on our confidence in the estimate of effect and is likely to change the estimate.

Very low quality: We are very uncertain about the estimate.

Abbreviations: CI, confidence interval; CSB, corn–soy blend; MAM, moderate acute malnutrition; RR, risk ratio; RUSF, ready‐to‐use supplementary food .

^a^ The basis for the assumed risk (e.g., the median control group risk across studies) is provided in footnotes. The corresponding risk (and its 95% CI) is based on the assumed risk in the comparison group and the relative effect of the intervention (and its 95% CI).

^b^ Downgraded due to study limitations.

^c^ Downgarded due to high heterogeneity.

^d^ Downgraded due to imprecision and small sample size.

**6. Summary of findings**

**Table:** 

Prophylactic antibiotic compared with no antibiotic for severe acute malnutrition (SAM)						
Patient or population: Children under 5 years of age with SAM						
Settings: Nutrition rehabilitation centre in low‐ and middle‐income countries						
Intervention: Prophylactic antibiotic						
Comparison: No antibiotic						
Outcomes	Illustrative comparative risks^a^ (95% CI)		Relative effect (95% CI)	No. of participants (studies)	Quality of the evidence (GRADE)	Comments
	Assumed risk	Corresponding risk				
	No antibiotic	Prophylactic antibiotic				
Recovery	762 per 1,000	804 per 1,000	RR: 1.06 (1.03 to 1.08)	5,166 participants (2 studies)	⊕⊕⊕⊕	One study Manary et al. (2012) contributed to two comparisons
					High	
(Assessed at 12 weeks)						
Weight gain	The mean weight gain ranged across control group from 3.1 g·kg^−1^·day^−1^ to 4 g·kg^−1^·day^−1^.	The mean weight gain in the intervention group was 0.67 g·kg^−1^·day^−1^ higher (0.28 g·kg^−1^·day^−1^ higher to 1.06 g·kg^−1^·day^−1^ higher) compared with the control group		5,052 participants (2 studies)	⊕⊕⊕⊝	One study Manary et al. (2012) contributed to two comparisons
(g·kg^−1^·day^−1^; assessed at 12 weeks)			Moderate^b^			
Mortality	70 per 1,000	53 per 1,000	RR: 0.74 (0.55 to 0.98)	6,944 participants (3 studies)	⊕⊕⊕⊝	One study Manary et al. (2012) contributed to two comparisons
					Moderate^b^	
(Assessed at 12 weeks)						

*Note*: GRADE Working Group grades of evidence.

High quality: Further research is very unlikely to change our confidence in the estimate of effect.

Moderate quality: Further research is likely to have an important impact on our confidence in the estimate of effect and may change the estimate.

Low quality: Further research is very likely to have an important impact on our confidence in the estimate of effect and is likely to change the estimate.

Very low quality: We are very uncertain about the estimate.

Abbreviations: CI, confidence interval, RR, risk ratio; SAM, severe acute malnutrition.

^a^ The basis for the assumed risk (e.g., the median control group risk across studies) is provided in footnotes. The corresponding risk (and its 95% CI) is based on the assumed risk in the comparison group and the relative effect of the intervention (and its 95% CI).

^b^ Downgraded due to high heterogeneity.

**7. Summary of findings**

**Table:** 

High dose vitamin A compared with low dose vitamin A for children with severe acute malnutrition (SAM)						
Patient or population: Children under 5 years of age with SAM						
Settings: Nutritional rehabilitation centre in low‐ and middle‐income country						
Intervention: High dose vitamin A						
Comparison: Low dose vitamin A						
Outcomes	Illustrative comparative risks^a^ (95% CI)		Relative effect (95% CI)	No. of participants (studies)	Quality of the evidence (GRADE)	Comments
	Assumed risk	Corresponding risk				
	Low dose vitamin A	High dose vitamin A				
Weight change	The change in weight across control group was 0.69 kg	The change in weight in the intervention groups was 0.05 kg higher in the intervention group (0.08 kg lower to 0.18 kg higher) compared with the control group.		207 participants (1 study)	⊕⊕⊕⊝	
			Moderate^b^	
(kg; change over 2 weeks)				
Mortality	0 per 1,000	29 per 1,000	RR: 7.07 (0.37 to 135.13)	207 participants (1 study)	⊕⊕⊕⊝	
					Moderate^b^
(At day 15)						

*Note*: GRADE Working Group grades of evidence.

High quality: Further research is very unlikely to change our confidence in the estimate of effect.

Moderate quality: Further research is likely to have an important impact on our confidence in the estimate of effect and may change the estimate.

Low quality: Further research is very likely to have an important impact on our confidence in the estimate of effect and is likely to change the estimate.

Very low quality: We are very uncertain about the estimate.

Abbreviations: CI, confidence interval; RR, risk ratio; SAM, severe acute malnutrition.

^a^ The basis for the assumed risk (e.g. the median control group risk across studies) is provided in footnotes. The corresponding risk (and its 95% confidence interval) is based on the assumed risk in the comparison group and the relative effect of the intervention (and its 95% CI).

^b^ Downgraded due to imprecision and small sample size.

## DATA AND ANALYSES

1. **Community‐based strategies to screen, identify and manage SAM and MAM compared with standard care**

**Table:** 

Outcome or subgroup	Studies	Participants	Statistical method	Effect estimate
1.1 Recovery	1	1,957	Risk ratio (M‐H, Random, 95% CI)	1.04 [1.00, 1.09]
1.2 Weight gain	1	1,957	Mean difference (IV, Random, 95% CI)	−0.80 [−0.82, −0.78]
1.3 Mortality	1	1,957	Risk ratio (M‐H, Random, 95% CI)	0.93 [0.60, 1.45]
1.4 Length gain	1	1,957	Mean difference (IV, Random, 95% CI)	−0.10 [−0.10, −0.10]
1.5 MUAC gain	1	1,957	Mean difference (IV, Random, 95% CI)	0.27 [0.27, 0.27]
1.6 Adverse events	1	3,914	Risk ratio (M‐H, Random, 95% CI)	0.79 [0.67, 0.93]
1.6.1 Diarrhoea	1	1,957	Risk ratio (M‐H, Random, 95% CI)	0.71 [0.60, 0.85]
1.6.2 Fever	1	1,957	Risk ratio (M‐H, Random, 95% CI)	0.85 [0.77, 0.93]
2. **Facility‐based strategies to screen and manage uncomplicated SAM according to the WHO protocol compared with other standards of care**

**Table:** 

Outcome or subgroup	Studies	Participants	Statistical method	Effect estimate
2.1 Recovery	1	60	Risk ratio (M‐H, Random, 95% CI)	1.00 [0.80, 1.25]
2.2 Mortality	2	473	Risk ratio (M‐H, Random, 95% CI)	1.21 [0.75, 1.94]
3. **RUTF versus F100 for SAM (facility based)**

**Table:** 

Outcome or subgroup	Studies	Participants	Statistical method	Effect estimate
3.1 Weight gain	3	266	Mean difference (IV, Random, 95% CI)	2.00 [−0.23, 4.23]
3.2 Weight gain (sensitivity analysis)	2	168	Mean difference (IV, Random, 95% CI)	0.91 [−2.15, 3.97]
3.3 Mortality	2	168	Risk ratio (M‐H, Random, 95% CI)	1.20 [0.34, 4.22]
3.4 Height	1	120	Mean difference (IV, Random, 95% CI)	−0.59 [−3.91, 2.73]
3.5 MUAC	1	120	Mean difference (IV, Random, 95% CI)	−0.66 [−4.78, 3.46]
3.6 Wasting	1	120	Risk ratio (M‐H, Random, 95% CI)	1.47 [0.85, 2.54]
4. **Community‐based management of children with uncomplicated SAM as outpatients with RUTF compared with standard diet, fortified blended flours (FBFs) or other locally produced foods**

**Table:** 

Outcome or subgroup	Studies	Participants	Statistical method	Effect estimate
4.1 Recovery	10	6,843	Risk ratio (IV, Random, 95% CI)	1.05 [1.00, 1.09]
4.1.1 Milk/peanut based RUTF versus non/reduced‐milk/peanut based RUTF	5	5,743	Risk ratio (IV, Random, 95% CI)	1.03 [0.99, 1.08]
4.1.2 RUTF versus energy dense home prepared food	4	959	Risk ratio (IV, Random, 95% CI)	1.14 [0.95, 1.36]
4.1.3 RUTF versus high oleic RUTF	1	141	Risk ratio (IV, Random, 95% CI)	1.06 [0.85, 1.31]
4.2 Recovery (sensitivity analysis)	9	6,661	Risk ratio (IV, Random, 95% CI)	1.06 [1.01, 1.11]
4.2.1 Milk/peanut based RUTF versus non/reduced‐milk/peanut based RUTF	5	5,743	Risk ratio (IV, Random, 95% CI)	1.03 [0.99, 1.08]
4.2.2 RUTF versus energy dense home prepared food	3	777	Risk ratio (IV, Random, 95% CI)	1.23 [0.99, 1.52]
4.2.3 RUTF versus high oleic RUTF	1	141	Risk ratio (IV, Random, 95% CI)	1.06 [0.85, 1.31]
4.3 Weight gain	8	5,205	Mean difference (IV, Random, 95% CI)	0.24 [−0.32, 0.79]
4.3.1 Milk/peanut based RUTF versus non/reduced‐milk/peanut based RUTF	3	3,069	Mean difference (IV, Random, 95% CI)	0.50 [0.02, 0.99]
4.3.2 RUTF versus F100	1	70	Mean difference (IV, Random, 95% CI)	5.50 [2.92, 8.08]
4.3.3 RUTF versus energy dense home prepared food	3	1,925	Mean difference (IV, Random, 95% CI)	−0.35 [−1.52, 0.82]
4.3.4 RUTF versus high oleic RUTF	1	141	Mean difference (IV, Random, 95% CI)	−0.80 [−1.74, 0.14]
4.4 Weight gain (sensitivity analysis)	6	3,845	Mean difference (IV, Random, 95% CI)	0.50 [−0.06, 1.06]
4.4.1 Milk/peanut‐based RUTF versus non/reduced‐milk/peanut‐based RUTF	3	3,069	Mean difference (IV, Random, 95% CI)	0.50 [0.02, 0.99]
4.4.2 RUTF versus F100	1	70	Mean difference (IV, Random, 95% CI)	5.50 [2.92, 8.08]
4.4.3 RUTF versus energy dense home prepared food	1	565	Mean difference (IV, Random, 95% CI)	0.41 [−0.16, 0.98]
4.4.4 RUTF versus high oleic RUTF	1	141	Mean difference (IV, Random, 95% CI)	−0.80 [−1.74, 0.14]
4.5 Mortaity	9	7,667	Risk ratio (IV, Random, 95% CI)	0.99 [0.69, 1.41]
4.5.1 Milk/peanut based RUTF versus non/reduced‐milk/peanut‐based RUTF	5	5,743	Risk ratio (IV, Random, 95% CI)	0.90 [0.72, 1.12]
4.5.2 RUTF versus energy dense home prepared food	2	1,743	Risk ratio (IV, Random, 95% CI)	1.87 [0.95, 3.70]
4.5.3 RUTF versus high oleic RUTF	1	141	Risk ratio (IV, Random, 95% CI)	5.07 [0.61, 42.31]
4.5.4 RUTF versus elevated *n*3 PUFA RUTF	1	40	Risk ratio (IV, Random, 95% CI)	0.33 [0.04, 2.94]
4.6 Mortaity (sensitivity analysis)	8	6,489	Risk ratio (IV, Random, 95% CI)	0.90 [0.64, 1.25]
4.6.1 Milk/peanut‐based RUTF versus non/reduced‐milk/peanut‐based RUTF	5	5,743	Risk ratio (IV, Random, 95% CI)	0.90 [0.72, 1.12]
4.6.2 RUTF versus energy dense home prepared food	1	565	Risk ratio (IV, Random, 95% CI)	5.09 [0.25, 105.53]
4.6.3 RUTF versus high oleic RUTF	1	141	Risk ratio (IV, Random, 95% CI)	5.07 [0.61, 42.31]
4.6.4 RUTF versus elevated *n*3 PUFA RUTF	1	40	Risk ratio (IV, Random, 95% CI)	0.33 [0.04, 2.94]
4.7 Height/Length gain	5	3,538	Mean difference (IV, Random, 95% CI)	−0.04 [−0.12, 0.04]
4.7.1 Milk/peanut‐based RUTF versus non/reduced‐milk/peanut‐based RUTF	2	2,037	Mean difference (IV, Random, 95% CI)	−0.56 [−2.29, 1.17]
4.7.2 RUTF versus energy dense home prepared food	2	1,360	Mean difference (IV, Random, 95% CI)	−0.07 [−0.11, −0.02]
4.7.3 RUTF versus high oleic RUTF	1	141	Mean difference (IV, Random, 95% CI)	−0.09 [−0.21, 0.03]
4.8 MUAC gain	6	3,612	Mean difference (IV, Random, 95% CI)	0.11 [−0.02, 0.24]
4.8.1 Milk/peanut‐based RUTF versus non/reduced‐ milk/peanut‐based RUTF	3	2,111	Mean difference (IV, Random, 95% CI)	0.68 [0.00, 1.36]
4.8.2 RUTF versus energy dense home prepared food	2	1,360	Mean difference (IV, Random, 95% CI)	−0.03 [−0.15, 0.08]
4.8.3 RUTF versus high oleic RUTF	1	141	Mean difference (IV, Random, 95% CI)	−0.07 [−0.17, 0.03]
4.9 Time to recovery	2	635	Mean difference (IV, Random, 95% CI)	−2.36 [−4.97, 0.25]
4.9.1 RUTF versus F100	1	70	Mean difference (IV, Random, 95% CI)	−3.90 [−6.04, −1.76]
4.9.2 RUTF versus energy dense home prepared food	1	565	Mean difference (IV, Random, 95% CI)	−1.21 [−1.92, −0.50]
4.10 Adverse events	3	3,343	Risk ratio (M‐H, Random, 95% CI)	1.06 [0.85, 1.32]
4.10.1 Cough/ALRI	2	1,093	Risk ratio (M‐H, Random, 95% CI)	0.97 [0.44, 2.16]
4.10.2 Diarrhoea	3	1,154	Risk ratio (M‐H, Random, 95% CI)	1.01 [0.83, 1.22]
4.10.3 Fever	2	1,096	Risk ratio (M‐H, Random, 95% CI)	1.21 [0.61, 2.39]
4.11 Hospitalisation	3	2,479	Risk ratio (M‐H, Random, 95% CI)	0.80 [0.46, 1.39]
5. **RUSF for MAM compared with standard diet, or FBF or other locally produced foods**

**Table:** 

Outcome or subgroup	Studies	Participants	Statistical method	Effect estimate
5.1 Recovery	10	8,409	Risk ratio (IV, Random, 95% CI)	1.05 [0.99, 1.11]
5.1.1 RUSF versus local/home made food	3	435	Risk ratio (IV, Random, 95% CI)	0.92 [0.64, 1.33]
5.1.2 RUSF versus whey RUSF	1	2,230	Risk ratio (IV, Random, 95% CI)	0.96 [0.92, 1.00]
5.1.3 RUSF versus CSB	6	5,744	Risk ratio (IV, Random, 95% CI)	1.07 [1.02, 1.13]
5.2 Recovery (sensitivity analysis)	9	8,336	Risk ratio (IV, Random, 95% CI)	1.06 [1.01, 1.12]
5.2.1 RUSF versus local/home made food	2	362	Risk ratio (IV, Random, 95% CI)	1.03 [0.69, 1.54]
5.2.2 RUSF versus whey RUSF	1	2,230	Risk ratio (IV, Random, 95% CI)	0.96 [0.92, 1.00]
5.2.3 RUSF versus CSB	6	5744	Risk ratio (IV, Random, 95% CI)	1.07 [1.02, 1.13]
5.3 Weight gain	7	6,657	Mean difference (IV, Random, 95% CI)	0.29 [−0.02, 0.59]
5.3.1 RUSF versus local/home made food	1	73	Mean difference (IV, Random, 95% CI)	−0.75 [−2.03, 0.53]
5.3.2 RUSF versus whey RUSF	1	2,230	Mean difference (IV, Random, 95% CI)	−0.16 [−0.33, 0.01]
5.3.3 RUSF versus CSB	5	4,354	Mean difference (IV, Random, 95% CI)	0.49 [0.10, 0.87]
5.4 Mortality	8	8,310	Risk ratio (IV, Random, 95% CI)	0.98 [0.57, 1.68]
5.4.1 RUSF versus whey RUSF	1	2,230	Risk ratio (IV, Random, 95% CI)	2.11 [0.39, 11.48]
5.4.2 RUSF versus CSB	6	5,744	Risk ratio (IV, Random, 95% CI)	0.92 [0.51, 1.67]
5.4.3 RUSF versus food supplement	1	336	Risk ratio (IV, Random, 95% CI)	0.56 [0.05, 6.08]
5.5 Length/Height gain	9	8,914	Std. mean difference (IV, Random, 95% CI)	−0.00 [−0.02, 0.01]
5.5.1 RUSF versus local/home made food	3	890	Std. mean difference (IV, Random, 95% CI)	−0.11 [−0.50, 0.28]
5.5.2 RUSF versus whey RUSF	1	2,230	Std. mean difference (IV, Random, 95% CI)	−0.01 [−0.03, 0.01]
5.5.3 RUSF versus CSB	6	5,794	Std. mean difference (IV, Random, 95% CI)	−0.00 [−0.02, 0.01]
5.6 Length/Height gain (sensitivity analysis)	8	8,841	Std. mean difference (IV, Random, 95% CI)	−0.01 [−0.02, 0.01]
5.6.1 RUSF versus local/home made food	2	817	Std. mean difference (IV, Random, 95% CI)	0.05 [−0.29, 0.39]
5.6.2 RUSF versus whey RUSF	1	2,230	Std. mean difference (IV, Random, 95% CI)	−0.01 [−0.03, 0.01]
5.6.3 RUSF versus CSB	6	5,794	Std. mean difference (IV, Random, 95% CI)	−0.00 [−0.02, 0.01]
5.7 MUAC gain	8	7,425	Std. mean difference (IV, Random, 95% CI)	0.08 [0.04, 0.11]
5.7.1 RUSF versus local/home made food	2	817	Std. mean difference (IV, Random, 95% CI)	0.22 [0.03, 0.41]
5.7.2 RUSF versus whey RUSF	1	2,230	Std. mean difference (IV, Random, 95% CI)	0.04 [0.02, 0.06]
5.7.3 RUSF versus CSB	6	4,378	Std. mean difference (IV, Random, 95% CI)	0.07 [0.03, 0.10]
5.8 Time to recovery	5	5,541	Mean difference (IV, Random, 95% CI)	−3.35 [−7.68, 0.97]
5.8.1 RUSF versus local/home made food	1	55	Mean difference (IV, Random, 95% CI)	−14.20 [−26.08, −2.32]
5.8.2 RUSF versus whey RUSF	1	2,230	Mean difference (IV, Random, 95% CI)	−1.10 [−2.73, 0.53]
5.8.3 RUSF versus CSB	3	3,256	Mean difference (IV, Random, 95% CI)	−2.77 [−8.39, 2.86]
5.9 Moderate stunting	1	170	Risk ratio (M‐H, Random, 95% CI)	0.85 [0.69, 1.05]
5.9.1 RUSF versus local/home made food	1	170	Risk ratio (M‐H, Random, 95% CI)	0.85 [0.69, 1.05]
5.10 Moderate wasting	2	1,539	Risk ratio (IV, Random, 95% CI)	0.95 [0.70, 1.28]
5.10.1 RUSF versus local/home made food	1	170	Risk ratio (IV, Random, 95% CI)	1.22 [0.34, 4.39]
5.10.2 RUSF versus CSB	1	1,369	Risk ratio (IV, Random, 95% CI)	0.93 [0.69, 1.27]
5.11 Severe wasting	3	3,256	Risk ratio (IV, Random, 95% CI)	0.74 [0.57, 0.95]
5.11.1 RUSF versus CSB	3	3,256	Risk ratio (IV, Random, 95% CI)	0.74 [0.57, 0.95]
5.12 Moderate underweight	1	170	Risk ratio (M‐H, Random, 95% CI)	1.06 [0.93, 1.22]
5.12.1 RUSF versus local/home made food	1	170	Risk ratio (M‐H, Random, 95% CI)	1.06 [0.93, 1.22]
5.13 Adverse events	3	12,476	Risk ratio (M‐H, Random, 95% CI)	1.09 [0.96, 1.25]
5.13.1 Fever	1	2,083	Risk ratio (M‐H, Random, 95% CI)	1.44 [0.95, 2.18]
5.13.2 Diarrhoea	3	4,022	Risk ratio (M‐H, Random, 95% CI)	1.08 [0.96, 1.22]
5.13.3 ALRI	1	2,083	Risk ratio (M‐H, Random, 95% CI)	0.98 [0.75, 1.29]
5.13.4 Vomiting	2	1,939	Risk ratio (M‐H, Random, 95% CI)	1.39 [1.03, 1.86]
5.13.5 Other illnesses	1	2,083	Risk ratio (M‐H, Random, 95% CI)	0.78 [0.56, 1.07]
5.13.6 Any adverse events	1	133	Risk ratio (M‐H, Random, 95% CI)	1.17 [0.61, 2.27]
5.13.7 Serious adverse events	1	133	Risk ratio (M‐H, Random, 95% CI)	2.03 [0.53, 7.78]
5.14 Hospitalisation	5	4140	Risk ratio (M‐H, Random, 95% CI)	0.76 [0.34, 1.70]
6. **Prophylactic use of antibiotics in children with uncomplicated SAM compared with no antibiotics**

**Table:** 

Outcome or subgroup	Studies	Participants	Statistical method	Effect estimate
6.1 Recovery	2	5,166	Risk ratio (IV, Random, 95% CI)	1.05 [1.03, 1.08]
6.2 Weight gain	2	5,052	Mean difference (IV, Random, 95% CI)	0.74 [0.40, 1.08]
6.3 Mortality	3	6,944	Risk ratio (IV, Random, 95% CI)	0.78 [0.55, 1.11]
6.4 MUAC gain	2	5,031	Mean difference (IV, Random, 95% CI)	0.06 [0.04, 0.08]
6.5 Length gain	2	5,052	Mean difference (IV, Random, 95% CI)	0.01 [‐0.02, 0.04]
6.6 Time to recovery	1	2,442	Mean difference (IV, Random, 95% CI)	−0.26 [−1.86, 1.34]
6.7 Adverse events	3	18,336	Risk ratio (IV, Random, 95% CI)	0.95 [0.87, 1.03]
6.7.1 Diarrhoea	3	6,707	Risk ratio (IV, Random, 95% CI)	1.01 [0.81, 1.27]
6.7.2 Respiratory symptoms	3	6,703	Risk ratio (IV, Random, 95% CI)	0.91 [0.86, 0.96]
6.7.3 Fever	2	4,926	Risk ratio (IV, Random, 95% CI)	0.96 [0.86, 1.06]
6.8 Hospitalisation	3	6,944	Risk ratio (M‐H, Random, 95% CI)	0.89 [0.82, 0.98]
7. **High‐dose vitamin A versus low‐dose vitamin A**

**Table:** 

Outcome or subgroup	Studies	Participants	Statistical method	Effect estimate
7.1 Weight change	1	207	Mean difference (IV, Random, 95% CI)	0.05 [−0.08, 0.18]
7.2 Mortality		1	207	Risk ratio (M‐H, Random, 95% CI)	7.07 [0.37, 135.13]
7.3 Height change	1	207	Mean difference (IV, Random, 95% CI)	0.10 [0.02, 0.18]
7.4 MUAC change	1	207	Mean difference (IV, Random, 95% CI)	0.80 [−0.46, 2.06]	
7.5 Adverse events	1	142	Risk ratio (M‐H, Random, 95% CI)	1.40 [0.46, 4.21]
7.5.1 Fever	1	122	Risk ratio (M‐H, Random, 95% CI)	1.50 [0.45, 5.05]	
7.5.2 ALRI	1	20	Risk ratio (M‐H, Random, 95% CI)	1.00 [0.07, 13.87]

## SOURCES OF SUPPORT

### Internal sources

- Aga Khan University, Other

### External sources

- BMGF, USA

Funding for this review came from a grant from the Bill & Melinda Gates Foundation to the Centre for Global Child Health at The Hospital for Sick Children (Grant No. OPP1137750).

## APPENDIX A SEARCH STRATEGY

**Table:**

PubMed Search Strategy (searched in title, abstract and/or keyword searches)
#1. "Infant"[Mesh]
#2. "Child, Preschool"[Mesh]
#3. Infant*
#4. Toddler*
#5. Baby OR babies
#6. Newborn* OR Neonat*
s#7. Preschool* OR Kindergarten* OR Under‐5s OR "Under 5 s" OR "Under 5"
#8. #1 OR #2 OR #3 OR #4 OR #5 OR #6 OR #7
#9. "Severe Acute Malnutrition"[Mesh]
#10. "Infant Nutrition Disorders"[Mesh]
#11. "Nutrition Disorders"[Mesh]
#12. "Severe Acute Malnutrition" OR SAM
#13. "Moderate Acute Malnutrition" OR MAM
#14. "Protein‐Energy Malnutrition"[Mesh]
#15. Undernutrition OR under‐nutrition
#16. Malnourish*
#17. Malnutrition
#18. Stunted OR wasted OR wasting OR "Wasting Syndrome"[Mesh]
#19. Starve* OR Starvat* OR "Starvation"[Mesh]
#20. "Vitamin A" OR "Vitamin A Deficiency" "Vitamin A"[Mesh]
#21. "Iron"[Mesh] OR "Iron deficiency" OR "Fe deficiency" OR "Anemia"[Mesh]
#22. Zinc OR "Zinc deficiency OR "Zn deficiency" OR "Zinc"[Mesh]
#23. #9 OR #10 OR #11 OR #12 OR #13 OR #14 OR #15 OR #16 OR #17 OR #18 OR #19 OR #20 OR #21 OR #22
#24. "Food"[Mesh]
#25. "Infant Food"[Mesh]
#26. "Food, Fortified"[Mesh]
#27. "Food, Formulated"[Mesh]
#28. "Dietary Supplements"[Mesh]
#29. "Fortified Food*"
#30. "Diet* Supplement*"
#31. "Ready to use therapeutic food" OR RUTF
#32. "Ready to use supplementary food" OR RUSF
#33. "Ready to use food*" OR RUF
#34. F100 OR F75
#35. CTC
#36. "Vitamin A Supplement*"
#37. "Micronutrient* Supplement*"
#38. "Dietary Fats"[Mesh]
#39. "Dietary Proteins"[Mesh]
#40. FBF
#41. "Corn soy*"
#42. "Wheat soy* blend*"
#43. "Rice mild blend*"
#44. "Milk rice blend*"
#45. "Pea wheat blend*"
#46. "Cereal pulse blend*"
#47. "Lipid‐based nutrient supplement*"
#48. Nutributter
#49. "Milk Proteins"[Mesh]
#50. "Community based management of malnutrition" OR CMAM
#51. "Amoxicillin"[Mesh]
#52. "Cotrimoxazole"[Mesh]
#53. Bacteraemia*
#54. Gentamicin
#55. "Penicillin G"[Mesh]
#56. "Chloramphenicol"[Mesh]
#57. "Ceftriaxone"[Mesh]
#58. "Ciprofloxacin"[Mesh]
#59. "Inpatient management" OR "In‐patient management" OR IMCI OR IMNCI
#60. "Community based management"
#61. "Facility based management"
#62. Prophyla* AND antibiotic*
#63. #24 OR #25 OR #26 OR #27 OR #28 OR #29 OR #30 OR #31 OR #32 OR #33 OR #34 OR #35 OR #36 OR #37 OR #38 OR #39 OR #40 OR #41 OR #42 OR #43 OR #44 OR #45 OR #46 OR #47 OR #48 OR #49 OR #50 OR #51 OR #52 OR #53 OR #54 OR #55 OR #56 OR #57 OR #58 OR #59 OR #60 OR #61 OR #62
#64. "Morbidity"[Mesh]
#65. "Mortality"[Mesh]
#66. Death*
#67. Relapse*
#68. Recovery
#69. #64 OR #65 OR #66 OR #67 OR #68
#70. #8 AND #23 AND (#63 OR #69)
#71. Age Filters Applied: Infants 1‐23 months; birth‐23 months; Preschool child 2‐5 years
